# Projected Climate Change Impacts on Deep‐Sea Corals of the Red Sea

**DOI:** 10.1111/gcb.71169

**Published:** 2026-11-08

**Authors:** Megan K. B. Nolan, Jorge Assis, Fabio Marchese, Natalie Dunn, Marta Ezeta Watts, Pauline Falkenberg, Nico Augustin, Thomas Lüdmann, Froukje M. van der Zwan, Hildegard Westphal, Mohammed Qurban, Vincent Pieribone, Carlos M. Duarte, Francesca Benzoni

**Affiliations:** ^1^ Division of Biological and Environmental Science and Engineering King Abdullah University of Science and Technology Thuwal Saudi Arabia; ^2^ Marine Science Program King Abdullah University of Science and Technology Thuwal Saudi Arabia; ^3^ Centro de Ciências do Mar do Algarve (CCMAR/CIMAR LA) Campus de Gambelas, Universidade do Algarve Faro Portugal; ^4^ Faculty of Biosciences and Aquaculture Nord University Bodo Norway; ^5^ GEOMAR Helmholtz Centre for Ocean Research Kiel Kiel Germany; ^6^ OceanQuest King Abdullah University of Science and Technology Thuwal Saudi Arabia; ^7^ Department of Earth System Sciences University of Hamburg Hamburg Germany; ^8^ Earth Science and Engineering Program, Physical Science and Engineering Division King Abdullah University of Science and Technology Thuwal Saudi Arabia; ^9^ Leibniz Centre for Tropical Marine Research (ZMT) Bremen Germany; ^10^ Department of Geosciences University of Bremen Bremen Germany; ^11^ National Center for Wildlife Development Riyadh Saudi Arabia; ^12^ OceanX New York New York USA

**Keywords:** climate change, deep‐sea coral, ecological niche models, Red Sea, shared socioeconomic pathways, species distribution

## Abstract

Ocean warming, acidification, and deoxygenation, among other co‐occurring factors, are shifting species ranges throughout the marine environment. Mesophotic and deep corals are understudied in their response to climate changes, particularly in the Red Sea, which has relatively high temperatures and salinities and is projected to undergo major changes in the future, with little assessment of the impacts to date. Ecological niche modelling can be used to explain the known distribution of a species, predict its current range, and estimate future range shift under changing ocean scenarios. Here, we estimate the area of suitable habitat for four mesophotic and deep‐sea corals (*Acanthogorgia* spp., *Stylaster tritoni, Bathypathes thermophila*, and *Madracis interjecta* ) across the whole Red Sea under present conditions and under different scenarios of future environmental changes (years 2090–2100). We found variations in the responses of these corals, but two of the four taxa are projected to lose significant amounts of their current habitats, while the other two lose a smaller portion. In particular, the scleractinian coral *M. interjecta* is likely to lose the largest proportion of its habitat, while the black coral *B. thermophila* may experience the least change. Deeper areas may become suitable for these corals, but colonisation will depend on their ability to disperse. We also assessed the potential biodiversity hotspots both now and in the future, finding the northern Red Sea to be one ecologically important area. These results enable marine spatial planning in support of an effective long‐term conservation action to protect these important habitats.

## Graphical abstract

Through modeling the distribution of four taxonomically diverse deep‐sea coral taxa under present and future climate conditions, we identify potential biodiversity hotspots of deep‐sea corals in the Red Sea, providing important considerations for international, long‐term management of these natural marine resources.

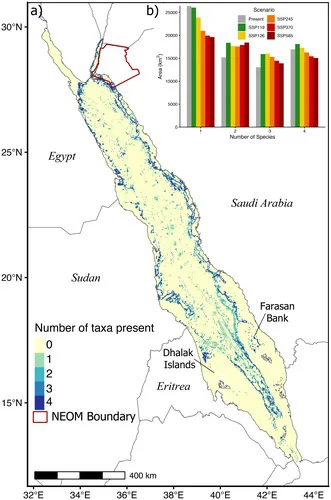

## 1 Introduction

Unprecedented and anthropogenically‐driven climate change is affecting biodiversity worldwide (Boonman et al. 2022; Habibullah et al. 2022). Corals are vulnerable to climate change, and have received attention due to their global importance (Hoegh‐Guldberg et al. 2017). Shallow coral reefs harbour high biodiversity (Sobha et al. 2023), support fishery (Teh et al. 2013) and tourism industries (Brander et al. 2007), and provide coastal protection (Burke and Spalding 2022). Similarly, in deeper waters, corals support biodiversity that encourages ecosystem functioning (Thurber et al. 2014), and increase resource retention and recycling in the deep sea (Maier et al. 2021). One of the more visible responses to climate change is the bleaching of zooxanthellate corals, recorded frequently since 1998 in shallow waters (van Woesik and Kratochwill 2022), and more recently, in the mesophotic (30–150 m depth; Diaz et al. 2023). Although the deep sea (> 200 m depth) is often considered far removed from the same pressures, the global deep ocean holds considerable heat, and is warming at a rate of 0.1°C per decade (Levin and Le Bris 2015). This could have dramatic effects for environments that are typically stable, where the organisms do not experience daily or seasonal fluctuations in environmental conditions (Levin et al. 2023). Even small temperature increases can result in changes in biodiversity (Danovaro et al. 2004), yet the physiological responses of deep‐sea corals to these threats remains relatively unknown.

Many of the environmental variables that are predicted to change under future climate conditions have been reported to influence the distribution of marine organisms. For example, temperature is often a strong predictor of distribution (e.g., Fredston et al. 2021) and is known to influence marine species. Although responses are variable among taxa, many motile species are shifting their range poleward (Hastings et al. 2020; Poloczanska et al. 2013; Simpson et al. 2011). Although long‐term monitoring of deep‐sea physical conditions is uncommon at the scales required for climate change studies (Levin and Le Bris 2015), some examples of range shifts in the deep (below 200 m depth) in response to warming oceans have been recorded. These include the invasion of the crab *Neolithodes yaldwyni* Ahyong & Dawson, 2006 in the Palmer Deep of Antarctica, where rapid warming is occurring (Smith et al. 2012). The predatory behaviour of these crabs has caused significant ecological impacts on native populations (Smith et al. 2012). Native populations are adapted to the very stable conditions of deep‐sea environments, which is likely to result in high vulnerabilities, and rates of environmental changes may ultimately decide the fates of deep‐sea organisms (Yasuhara and Danovaro 2016), particularly as deep‐sea benthic fauna exhibit slow life histories (Waller et al. 2023).

In addition to increasing temperatures, oceans are also experiencing acidification due to increased absorption of atmospheric CO_2_. In particular, this affects organisms that form calcium carbonate skeletons, including deep‐sea corals (Anderson et al. 2022). In shallow water, corals have been found to either reduce their growth rates (Guinotte et al. 2003), or to grow at the same rate, or even faster (Guillermic et al. 2021), preserving linear extension at the cost of skeletal density, resulting in a weaker skeleton (Mollica et al. 2018). The same issues are present in the deep sea, and deep‐sea corals grow in high saturation levels of aragonite, a crystal form of calcium carbonate which they use for skeletal growth (Guinotte et al. 2006). Both the aragonite saturation horizon and the calcite saturation horizon, which affects octocorals and some stylasterids, are shoaling in many ocean basins, potentially reducing the suitable habitat for deep‐sea corals (Guinotte et al. 2006; Turley et al. 2007).

Increasing seawater temperatures and acidification interact with dissolved oxygen concentrations. The capacity of the ocean to hold oxygen reduces with warming, resulting in increased deoxygenation and stratification of the water column (Brewer and Peltzer 2009; Levin and Le Bris 2015). Consequently, the oxygen minimum zone (OMZ) that is present in most oceans is expanding (Keeling et al. 2010). This is constricting the range of many species into shallower waters (Stramma et al. 2011), while expanding that of species tolerant to low oxygen (Gilly et al. 2013). Deep‐sea corals have also shown to be sensitive to low oxygen conditions (Lunden et al. 2014), and coral‐dominated ecosystems are at risk (Roch et al., Forthcoming; Ross et al. 2020). As shown, the tolerances of species are variable depending on their physiologies and life‐histories (e.g., Gilly et al. 2013; Simpson et al. 2011). Regardless, the biodiversity of these ecosystems is likely to decline, which in turn will impact ecosystem functioning (Thurber et al. 2014).

An understanding of the current spatial distribution of corals can provide us with fundamental knowledge of the conditions required for their growth and functioning, and consequently can aid in understanding the potential responses of these species to future climate change (Helaouët and Beaugrand 2009). Additionally, the areas of range overlap under current and future conditions provide vital information for management, as this is where species are most likely to be able to survive in the long term (Schwartz 2012). This has practical applications for marine spatial planning, as it ensures the long‐term efficacy and relevancy of marine protected areas and fishery management (Guisan et al. 2013). The high cost associated with visual surveys below SCUBA diving limits creates challenges in comprehensively understanding the distribution of deep‐sea species. Technologies like remotely operated vehicles (ROVs) or submersibles have improved this knowledge, but are expensive and therefore not always available (Amon et al. 2022). Even with low‐cost technologies like the Azor drift cam (Dominguez‐Carrió et al. 2021), low visibility and low‐light conditions in the deep sea mean that the field of view is narrow and the actual observed area is small (Bell et al. 2025). This results in relatively few in situ observations of many species, and for example, has yielded a large proportion of deep‐sea species remaining data deficient or unassessed by the International Union for the Conservation of Nature (IUCN) Red List (Polidoro et al. 2011).

Ecological niche models (ENMs), which follow a similar fundamental methodology as habitat suitability models (HSMs), can use these observations in presence‐pseudo‐absence modelling to provide a greater picture of the potentially suitable habitat of these species of interest, which can then be related to their niche (Guinan et al. 2009). These methods rely on accurate presence records to correlate occurrences to environmental and geomorphometric data to predict across unknown spatial or temporal scales (Elith and Leathwick 2009). There are several potential methods for ENMs; one of the most effective and commonly used methods is Boosted Regression Tree models (BRTs; Elith et al. 2008), which combine multiple models to improve model performance (Elith et al. 2008). Additionally, MaxEnt has been shown to perform well for presence‐only modelling (Elith and Graham 2009).

The impacts of climate change on the distribution of deep‐sea corals and coral‐dominated ecosystems have been previously studied through predictive modelling. For example, the distribution of 12 scleractinian corals was predicted in New Zealand under two future climate scenarios, finding a general decline in suitable habitat (Anderson et al. 2022) that informed spatial planning analyses (Stephenson et al. 2023). Similar studies have also been carried out in the North Atlantic, comparably finding dramatic habitat loss for deep‐sea corals (Morato et al. 2020). In the Red Sea, habitat suitability models have recently been used to understand the distribution of deep‐sea corals under current conditions (Nolan et al. 2024, 2025), but no attempt has been made to map how the distributions of Red Sea corals may shift under future climate change conditions, precluding proper biodiversity conservation and management.

In shallow coral reef studies, the Red Sea has been used as a model to understand the potential tolerances of corals under climate change (Fine et al. 2013; Savary et al. 2021), due to high seawater temperatures and salinities (Berumen et al. 2019). The deep Red Sea is similarly unique, with temperatures that remain above 21°C throughout the water column, and salinities that stabilise close to 40 PSU (Roder et al. 2013). These conditions provide a unique setting for deep‐sea corals and could be valuable for understanding the tolerances of corals outside the Red Sea under future climate conditions. However, the Red Sea itself is not immune to the effects of climate change, and research has shown that the basin is undergoing rapid warming (Agulles et al. 2021; Raitsos et al. 2011), influenced by patterns of upwellings (El Kenawy et al. 2025). Through the sixth phase of the Coupled Model Intercomparison Project (CMIP6), models have been generated on global projected climate scenarios, which, in combination with differing scenarios of future global development, have informed the Shared Socioeconomic Pathways (SSPs; van Vuuren et al. 2013). This provides a comprehensive framework under which to study future global environmental conditions and their impacts on regional biodiversity changes.

Here, we focus on understanding the current spatial distribution of the suitable habitat of four cnidarians from the Red Sea, selected to represent the biodiversity of the mesophotic and deep zones. We also aim to predict the distribution of these taxa under five future SSPs. The focal taxa are *Acanthogorgia* spp. Gray, 1857 (Anthozoa: Octocorallia: Malacalcyonacea), *Bathypathes thermophila* Chimienti, 2022 (Anthozoa: Hexacorallia: Antipatharia), *Stylaster tritoni* Maggioni, Cairns, Pica & Benzoni, 2022 (Medusozoa: Hydrozoa: Anthoathecata) and *Madracis interjecta* Marenzeller, 1907 (Anthozoa: Hexacorallia: Scleractinia). Representing four orders, they include two recently described Red Sea endemic species (*S. tritoni* Maggioni et al. (2022) and *B. thermophila* (Chimienti et al. 2022)), an octocoral genus with a broad depth range (Macrina et al. 2024, 2025) and a Scleractinian species that has been shown to form bioherm structures in the mesophotic zone (Fricke and Hottinger 1983; Nolan et al. 2025).

## 2 Materials and Methods

### 2.1 Expeditions

Six oceanographic expeditions took place in the Red Sea between 2020 and 2023. Here, we have compiled data from three expeditions on board R/V OceanXplorer‐ the DeepBlue expedition (October–November 2020), the Red Sea Decade Expedition (February–June 2022) and the Relationship Cultivation Mission (June–July 2022). We combined this with data from the KAUST Red Sea 5 (KRSE5) expedition onboard R/V Aegaeo (February 2023) and two expeditions onboard R/V Meteor: M193 REPLENISH (September 2023) (Lüdmann et al. 2023) and M194 HEXPLORES (October 2023) (Augustin et al. 2024). Equipment and instrumentation differed between expeditions and vessels (Table 1), but bathymetric data was acquired by four of the expeditions and was combined. The differences in calibration of the Conductivity, Temperature, Depth (CTD) sensors meant that water column parameters were only used from one expedition (OceanX RSDE). Finally, all six expeditions provided benthic video transects using Remotely Operated Vehicles (ROVs) or submersibles (Supplementary Figure 1). All relevant instrumentation information is provided in Table 1, and described in papers elsewhere (e.g., Nolan et al. 2024; Vimercati et al. 2024).

**TABLE 1:** Metadata and quantity of video, acoustic and oceanographic data gathered for each of the expeditions included in this study.

Expedition	Dates	Area of interest	ROV	Submersible	Number of video transects	Multibeam	Area of bathymetry collected	CTD casts	Number of CTD casts
OceanX Deep Blue	07/10/2020–17/11/2020	Northern Red Sea/Gulf of Aqaba	ARGUS Mariner 108 XL ROV	2× Triton 3300/3	113	Kongsberg EM304	33,970 km^2^	—	—
OceanX RSDE	04/02/2022–18/06/2022	Saudi Arabian Red Sea waters	ARGUS Mariner 108 XL ROV	2× Triton 3300/3	147	Kongsberg EM304	49,740 km^2^	RBR *maestro*3	146
OceanX RC	20/06/2022–03/07/2022	Northern Red Sea/Gulf of Aqaba	ARGUS Mariner 108 XL ROV	2× Triton 3300/3	36	—	—	—	—
Aegaeo KRSE5	02/02/2023–17/02/2023	Central/North Red Sea, central axis	ROV MaxRover	—	6	—	—	—	—
Meteor M193 REPLENISH	08/09/2023–06/10/2023	Outside Al Wajh Lagoon	MARUM ROV Squid	—	8	Kongsberg EM710 + EM122	9662 km^2^	—	—
Meteor M194 HEXPLORES	10/10/2023–06/11/2023	Red Sea central axis, central/south	ROV Kiel 6000	—	15	—	—	—	—

*Note:* This does not detail all of the activities on board the expeditions, only those that were used in subsequent analysis for this paper. CTD casts were only included from one expedition onboard R/V OceanX (RSDE) due to potential small differences between instrumentation that could not be accounted for. Probes were Marine CT for temperature, Marine CT 2000 m for conductivity and RBRcoda T.ODO|fast for dissolved oxygen concentration. All ROV dives are counted here even if they are absent for all taxa. Some areas of collected bathymetry overlap and therefore the sum of the areas provided here is higher than that reported in text.

Abbreviations: CTD, Conductivity, Temperature and Depth; KRSE, KAUST Red Sea Expedition; RC, Relationship Cultivation Mission; ROV, remotely operated vehicle; RSDE, Red Sea Decade of Exploration Expedition.

### 2.2 Acoustic Data

Bathymetric data was acquired during the OceanX DeepBlue, the OceanX RSDE, and the Meteor M193 expeditions and processed to a resolution of 30 m using QPS (Table 1) (Marchese et al. 2026). Additional high‐resolution data was downloaded from Pangaea (Augustin et al. 2016, 2020). This data was combined in ArcGIS Pro (v. 3.2.0) and resampled using averaging to 400 m resolution. An 800 m buffer was then applied to generate a shapefile around the high‐resolution bathymetry. Bathymetric data for the Red Sea was downloaded from the General Bathymetric Chart of the Oceans (GEBCO) at a 400 m resolution (GEBCO Bathymetric Compilation Group 2023), and clipped to cover the areas without high‐resolution bathymetry. We combined the bathymetric datasets from both sources, using a kriging method in ArcGIS Pro to interpolate across the 800 m buffer, removing artefacts in the data where adjacent bathymetry is not in agreement.

With this combined 400 m dataset across the Red Sea, we were able to extract geomorphometric variables in ArcGIS Pro, following the methods of Bargain et al. (2017). The extracted variables were eastness and northness (Horn 1981), planar curve and profile curve (Zevenbergen and Thorne 1987), slope (Zevenbergen and Thorne 1987), and Vector Ruggedness Measure (VRM; Sappington et al. 2007). VRM explains bathymetric ruggedness as the variation in the orientation of neighbouring cells, and increased values may indicate an increase in habitat variability and therefore the presence of a suitable substrate. VRM was extracted with a small window size of three cells due to the coarse resolution of the final bathymetry dataset.

### 2.3 Water Column Data

CTD casts were conducted at 146 stations during the OceanX RSDE expedition, collecting temperature, salinity, and oxygen concentration data throughout the water column. All CTD data were cleaned to remove the top 5 m of the water column, where conditions are variable, as well as measurements from anomalous areas such as brine pools and low oxygen sinkholes (Klein et al. 2025). This final step was included to avoid any highly localised or anomalous readings from skewing the modeled values in the surrounding areas.

### 2.4 Environmental Rasters

Water column and geomorphometric data collected during the expeditions, in combination with Copernicus marine data, allowed us to generate environmental rasters using the Bio‐Oracle pipeline (Assis et al. 2017, 2024; Tyberghein et al. 2012). By utilising depth data extracted from GEBCO (GEBCO Bathymetric Compilation Group 2023), we were able to increase the spatial resolution of the gridded layers from 5 km to 400 m across the Red Sea. To achieve this uniform spatial extent, statistics were interpolated using a kriging algorithm that performed trilinear interpolation based on the longitude, latitude, and depth of each cell across multiple vertical levels (Assis et al. 2017; Boavida et al. 2016). The incorporation of CTD temperature, salinity, and oxygen concentrations resulted in more accurate data overall, although there is a bias towards the eastern side of the Red Sea. The remaining environmental variables (phosphate concentration, nitrate concentration, silicate concentration, pH, productivity, velocity X and velocity Y) were generated using existing data points in Bio‐Oracle, in combination with the higher resolution bathymetry. For all water column rasters, mean values over the decade 2010–2020 were used, calculated based on the mean seafloor depth within the cell. All rasters were used at a final resolution of 400 m.

Rasters were generated in Bio‐Oracle for the current climate, including minimum and maximum values, as well as five projected scenarios for the years 2090–2100, following the Shared Socioeconomic Pathways (SSPs; O'Neill et al. 2014). These scenarios combine predictions of socioeconomic changes over the next few decades with Representative Concentration Pathways (RCPs) of projected atmospheric greenhouse gas concentrations, to represent scenarios that range from positive to severe. The pathways are SSP1‐1.9, SSP1‐2.6, SSP2‐4.5, SSP3‐7.0 and SSP5‐8.5. The first value (1, 2, 3 or 5) is the socioeconomic approach considered in the pathway, from ‘sustainability’ to ‘fossil‐fuelled development’, and the second value (1.9, 2.6, 4.5, 7.0 or 8.5) refers to the RCP pathway, related to emissions of greenhouse gases and aerosols as well as patterns of land use over time (Moss et al. 2010). More details and estimates of the global environmental conditions under each of these pathways can be found in Chen et al. (2021).

### 2.5 Presence Data for Coral Taxa

During each of the expeditions, cameras mounted onto an ROV recorded georeferenced benthic video transects. On three expeditions (OceanX DeepBlue, RSDE, and RC), manned submersibles were also used (Table 1). From a total of 325 benthic transects (Table 1), we extracted presence location data for four taxa: two recently described Red Sea endemic species, *B. thermophila* and *S. tritoni*, the Indo‐Pacific Scleractinian coral *Madracis interjecta* , as well as the globally distributed octocoral genus *Acanthogorgia*. This process was aided by presences extracted for previous studies, including *S. tritoni* and *B. thermophila* from the DeepBlue expedition (Chimienti et al. 2022; Maggioni et al. 2022), and *M. interjecta* from all OceanX expeditions (Nolan et al. 2025). Observations of *Acanthogorgia* spp. are also published in Macrina et al. (2025). Additional occurrence records for *Acanthogorgia* spp. were downloaded from the Global Biodiversity Information Facility (GBIF) database, based on human observations in Egypt (GBIF.org 2024). No observations were found in online databases for any other taxa. Presence data were spatially thinned, resulting in only one observation per 400 × 400 m cell, and reducing any errors that may arise from spatial autocorrelation. We compared two methods to generate two types of absences: inferred absences were taken from surveys where the taxon was not identified, while pseudo‐absences were generated to be dispersed across space and environmental conditions. Due to the bias among survey sites, particularly towards the eastern Red Sea (Supplementary Figure 2), the pseudo‐absences were determined to be more representative of the area, and we developed the models with a presence‐pseudo‐absence method.

### 2.6 Ecological Niche Model Development

Models were developed in R (R Core Development Team 2020). As in Gouvêa et al. (2024), minimum and maximum variables were used for current velocity and temperature as they can identify anomalous conditions that may reduce species survival (e.g., Selig et al. 2010). Pearson's correlation and Variance Inflation Factor (VIF) were used to identify correlation and multicollinearity between variables. No strict cut off was used, and instead the results of the two analyses were considered, together with the importance of variables, following the methods of Taranto et al. (2023). This resulted in the removal of some variables with a high correlation. For example, initial trial models used mean salinity and minimum oxygen as predictor variables, but their lack of contribution to any of the models, and high correlation with depth and temperature variables led to their removal. Due to extremely low VIF scores, and an importance in explaining coral distribution (Supplementary Figure 9b,c), maximum temperature and depth were both retained in three of the models (for *Acanthogorgia* spp., *M. interjecta* and *S. tritoni*), despite a Pearson's correlation of 0.8. All other variables included had a Pearson's correlation ≤ 0.7. VIF scores for all remaining variables were below 5, well below the common threshold of 10 (Naimi and Araújo 2016). The set of predictor variables ultimately used in the models (Supplementary Table 1) was determined based on current understanding of the species and the relevance of geomorphometric parameters at 400 m resolution. Where studies on our species of interest were not available (e.g., due to restricted ranges, relatively new descriptions or remote distributions), we refer to literature on the genus or family. Variables included those which are known to influence the settlement of corals (depth, slope and VRM), the feeding of corals (velocities) and their growth and metabolism (temperature and pH; Supplementary Table 1).

*S. tritoni* and *M. interjecta* are found in a relatively narrow depth range (Maggioni et al. 2022; Nolan et al. 2025), resulting in a large area of the Red Sea with unsuitable conditions. To avoid the overrepresentation of unsuitable areas among pseudo‐absence points, we selected pseudo‐absences using a clustering method. We scaled all variables to be used in the model using zero mean standardisation, and then carried out k‐means clustering, where k was equal to 10 times the number of presences. This ensured pseudo‐absence points were well distributed across different physical and environmental conditions. Each time the model was run, a single, unique pseudo‐absence point was selected from each of the clusters, resulting in a pseudo‐absence data set that was 10 times greater than the number of presences (Barbet‐Massin et al. 2012).

To generate the final models, an ensemble method was used, averaging the results of two approaches: boosted regression trees (BRTs) were used due to their high performance with presence‐only data, the ability to force monotonicity, and the potential for hyperparameter tuning (Elith et al. 2008). The BRT models were complemented with MaxEnt models, which have also been shown to perform well with presence‐only data (Elith and Graham 2009). Development was carried out in R using the packages ‘ENMeval’ (Kass et al. 2021), ‘sdmpredictors’ (Bosch and Fernandez 2023), ‘gbm’ (Ridgeway and Developers 2024), and ‘SDMtune’ (Vignali et al. 2020). The BRT models were trained with a Bernoulli distribution, and to reduce overfitting and introduce stochasticity, a bag fraction of 0.5 was used. Gridsearch trials were run for each species in order to determine the best balance of the shrinkage parameter (also known as the learning rate, lr), the depth interaction (also called tree complexity), and the number of trees, aiming to maximise the evaluation metrics of the test dataset. Maxent was trained with a regularisation parameter of 2.5 to avoid overfitting, with hinge features enabled and 500 iterations. For either model, any variables with a contribution less than 5% to any run were removed, and the model was rerun without those variables; this resulted in the most parsimonious models which reduced the potential for overfitting to the training data. This process was only conducted once per run, and since the contributions changed when the model was rerun with different variables, this may have resulted in variables with a low contribution to the final models. To increase generality and improve the transferability of the models, monotonicity was forced for all relevant variables in BRT models, applying a positive forcing on ‘minimum’ predictors, and a negative forcing on ‘maximum’ predictors (e.g., Hofner et al. 2011). This was based on current available literature and hypothesised responses.

Ten model runs were carried out with different pseudo‐absence data, and all data was averaged across these runs. Within each of these runs, presence and pseudo‐absences were split into four spatial blocks for cross‐validation that ensured a minimal effect of spatial auto‐correlation and allowed the assessment of model performance in new areas. Model accuracy was evaluated with the threshold independent Area Under the Receiver Operating Characteristics (ROC) Curve (AUC), as well as the threshold dependent True Skills Statistic (TSS; Allouche et al. 2006). The threshold for the TSS score was calculated by maximising the sum of sensitivity and specificity (MaxSSS; Liu et al. 2016). We calculated sensitivity and Boyce's index to indicate the ability of the models to identify true presences. Agreement between the two model algorithms (BRT and Maxent) was also assessed. The absolute difference between the habitat suitability index for each model was calculated, a difference of < 0.1 was considered high agreement, 0.1–0.3 was considered medium agreement, while a difference > 0.3 was considered low agreement.

### 2.7 Prediction of Suitable Habitat for Study Corals in Mesophotic and Deep Red Sea

Each of the 10 models per taxa was used to estimate the fundamental niches, which were projected onto geographic space in order to generate maps of the suitable habitat of the species. This area was assessed across the whole Red Sea, and the models were combined by averaging. Standard deviation between the runs was also calculated. The MaxSSS threshold was used to convert the results to binary presence or absence (Yesson et al. 2017). This process was repeated for each of the five SSPs, using the same variables under future estimated conditions. The suitable area (presence) was calculated as a function of the total area of the Red Sea, and future scenarios were compared to present‐day predictions. Due to the non‐parametric nature of most of this data (Shapiro–Wilk test, *p* < 0.05), we chose to test significance using a Wilcoxon rank‐sum test to allow comparisons between groups.

## 3 Results

### 3.1 Geomorphometric and Environmental Data

High‐resolution bathymetry, obtained across a total area of 79,654 km^2^ across the Red Sea, primarily the eastern Red Sea was combined with 275,179 km^2^ of low‐resolution bathymetry from GEBCO (Supplementary Figure 3), covering a depth range from 0 to 2800 m. The raster generated for mean seafloor temperature ranges from 14.9°C to 30.5°C. While the lower limit is around 5°C–6°C cooler than reported for the Red Sea, these anomalous areas are restricted to a few small and deep regions (70% of the area was deeper than 1500 m), where our study taxa are not expected. Hence, these discrepancies refer to the depth domains not relevant for our study. Mean oxygen concentration ranged from 1.0 to 219.5 μmol O_2_ l^−1^, and mean salinity ranged from 35.0 to 42.0 PSU. This improved bathymetric dataset, and the in situ CTD measurements allowed us to reprocess the Bio‐Oracle data from a nascent resolution of 0.05° (approximately 5 km) to 400 m (Supplementary Figure 4).

### 3.2 Presence Data for Coral Taxa

A total of 653 observed presences were recorded for the four coral taxa (Supplementary Figure 1). Records for *Acanthogorgia* came mostly from Saudi Arabian waters, aside from two records from GBIF, recorded in Egyptian waters. The total number of records for *Acanthogorgia* spp. was 105, between 77 and 1453 m depth (average 295.71 m) and with a latitudinal range from 16.862° to 29.303° N (average 22.622° N). *B. thermophila* was observed 96 times between 101 and 720 m depth (average 362.74 m), and from 16.866°–29.265° N (average 24.117° N). *M. interjecta* was observed 392 times between 83 and 280 m (average 160.72 m) and from 17.197° to 29.304° N (average 27.971° N). Observations of *S. tritoni* totalled 84, between 59 and 603 m (average 292.93 m) and from 16.931° to 28.456° N (average 21.154° N) (Supplementary Table 2). After data was thinned for the modelling step, there were 67 unique records of *Acanthogorgia* spp., 62 of *B. thermophila*, 39 of *M. interjecta* and 38 of *S. tritoni*.

### 3.3 Final Ecological Niche Models (ENM)

A separate ENM was generated for each of the four coral taxa. Depth was used as a variable in each of them, along with two derived geomorphometric variables: slope and VRM. For the oceanographic variables, the same combination was used for the models for *Acanthogorgia* spp., *M. interjecta* and *S. tritoni*, they were: mean pH, maximum temperature, minimum temperature, minimum velocities (x and y), and maximum velocities (x and y). For the model for *B. thermophila*, the same variables were used, with the exception of mean temperature in place of maximum and minimum temperatures. This was determined through initial competitive models and evaluated with the AUC and TSS. In all cases, models performed well, with an average AUC across both model algorithms between 0.87 and 0.95, and an average TSS between 0.66 and 0.86 (Table 2). The MaxSSS that served as the threshold for calculating both the TSS and the presence or absence of species was between 0.13 and 0.27 (Table 2). Mean sensitivity and Boyce's index indicated a good ability of the models to determine true presences of the species, averaging between 0.86 and 0.97, and 0.41 and 0.93 respectively. The BRT model for *S. tritoni* returned a Boyce's index score of 0.417, representing the lowest ability of model distinction. The interaction depth and shrinkage parameters were tuned for each individual model run, and the averages of these are reported in Supplementary Table 3.

**TABLE 2:** Accuracy metrics and thresholds for each modelling approach, and the overall means, including standard deviation and standard errors.

		*Acanthogorgia* spp.	*Bathypathes thermophila*	*Madracis interjecta*	*Stylaster tritoni*
Overall	**Mean AUC**	**0.911**	**0.935**	**0.914**	**0.888**
	AUC Standard Deviation	0.016	0.013	0.058	0.081
	AUC Standard Error	0.004	0.003	0.013	0.018
	**Mean TSS**	**0.712**	**0.786**	**0.790**	**0.730**
	TSS Standard Deviation	0.063	0.034	0.094	0.150
	TSS Standard Error	0.014	0.008	0.021	0.033
	**Mean Threshold**	**0.181**	**0.200**	**0.185**	**0.215**
	Threshold Standard Deviation	0.079	0.075	0.167	0.176
	Threshold Standard Error	0.018	0.017	0.037	0.039
	**Mean Sensitivity**	**0.922**	**0.929**	**0.916**	**0.931**
	Sensitivity Standard Deviation	0.041	0.029	0.079	0.049
	Sensitivity Standard Error	0.009	0.007	0.018	0.011
	**Mean Boyce's Index**	**0.883**	**0.884**	**0.679**	**0.645**
	Boyce's Index Standard Deviation	0.078	0.064	0.242	0.420
	Boyce's Index Standard Error	0.017	0.014	0.054	0.094
Boosted Regression Trees	**Mean AUC**	**0.905**	**0.935**	**0.950**	**0.894**
	AUC Standard Deviation	0.021	0.017	0.047	0.112
	AUC Standard Error	0.007	0.005	0.006	0.035
	**Mean TSS**	**0.660**	**0.765**	**0.856**	**0.732**
	TSS Standard Deviation	0.046	0.035	0.059	0.216
	TSS Standard Error	0.015	0.011	0.018	0.068
	**Mean Threshold**	**0.134**	**0.139**	**0.194**	**0.161**
	Threshold Standard Deviation	0.045	0.029	0.175	0.229
	Threshold Standard Error	0.014	0.009	0.055	0.072
	**Mean Sensitivity**	**0.908**	**0.925**	**0.964**	**0.960**
	Sensitivity Standard Deviation	0.047	0.038	0.031	0.045
	Sensitivity Standard Error	0.015	0.012	0.010	0.014
	**Mean Boyce's Index**	**0.845**	**0.859**	**0.676**	**0.417**
	Boyce's Index Standard Deviation	0.088	0.076	0.308	0.505
	Boyce's Index Standard Error	0.028	0.024	0.097	0.160
MaxEnt	**Mean AUC**	**0.917**	**0.934**	**0.877**	**0.882**
	AUC Standard Deviation	0.006	0.008	0.042	0.033
	AUC Standard Error	0.002	0.002	0.013	0.010
	**Mean TSS**	**0.764**	**0.807**	**0.725**	**0.728**
	TSS Standard Deviation	0.016	0.014	0.070	0.030
	TSS Standard Error	0.005	0.004	0.021	0.009
	**Mean Threshold**	**0.229**	**0.261**	**0.176**	**0.269**
	Threshold Standard Deviation	0.074	0.048	0.159	0.077
	Threshold Standard Error	0.022	0.015	0.048	0.023
	**Mean Sensitivity**	**0.937**	**0.933**	**0.869**	**0.903**
	Sensitivity Standard Deviation	0.028	0.017	0.080	0.034
	Sensitivity Standard Error	0.009	0.005	0.024	0.010
	**Mean Boyce's Index**	**0.922**	**0.910**	**0.682**	**0.873**
	Boyce's Index Standard Deviation	0.039	0.037	0.161	0.050
	Boyce's Index Standard Error	0.012	0.011	0.049	0.015

*Note:* AUC is the Area Under the Receiver Operating Curve (ROC) Characteristic, and TSS is the True Skills Statistic. The threshold ensures the Maximum Sum of Sensitivity and Specificity (MaxSSS). Mean values are provided in bold.

Model results were similar between BRT and Maxent algorithms (Supplementary Figures 18–25), and agreement was high for > 88% of the area for three of the four taxa under all scenarios (Supplementary Figures 26–28). The exception was *S. tritoni*, for which model agreement dropped to between 57.9% and 68.5% (Supplementary Figure 29). For these models, a larger proportion of the area was classified as having medium agreement, and areas of low agreement remained below 5% under all scenarios, although some of those areas fell in spatially important regions such as the Farasan Bank and Dhalak Islands.

Between four and nine variables were selected for the final models, always including depth, VRM, and usually a measure of temperature, with the exception of the MaxEnt model for *B. thermophila* (Figure 1a). VRM was the most informative variable for both *Acanthogorgia* spp. (72.3% and 64.3% for BRT and MaxEnt respectively) and *B. thermophila* (61.5% and 57.8%), as well as the MaxEnt model for *S. tritoni* (42.8%). Additionally, VRM still contributed substantially to the models for *M. interjecta* (29.3% and 28.5%) and the BRT for *S. tritoni* (18.3%). The most informative variable for both modelling approaches for *M. interjecta* was depth (32.3% and 46.8%), while maximum temperature was most important for the BRT for *S. tritoni* (38.8%) (Figure 1).

**FIGURE 1:**
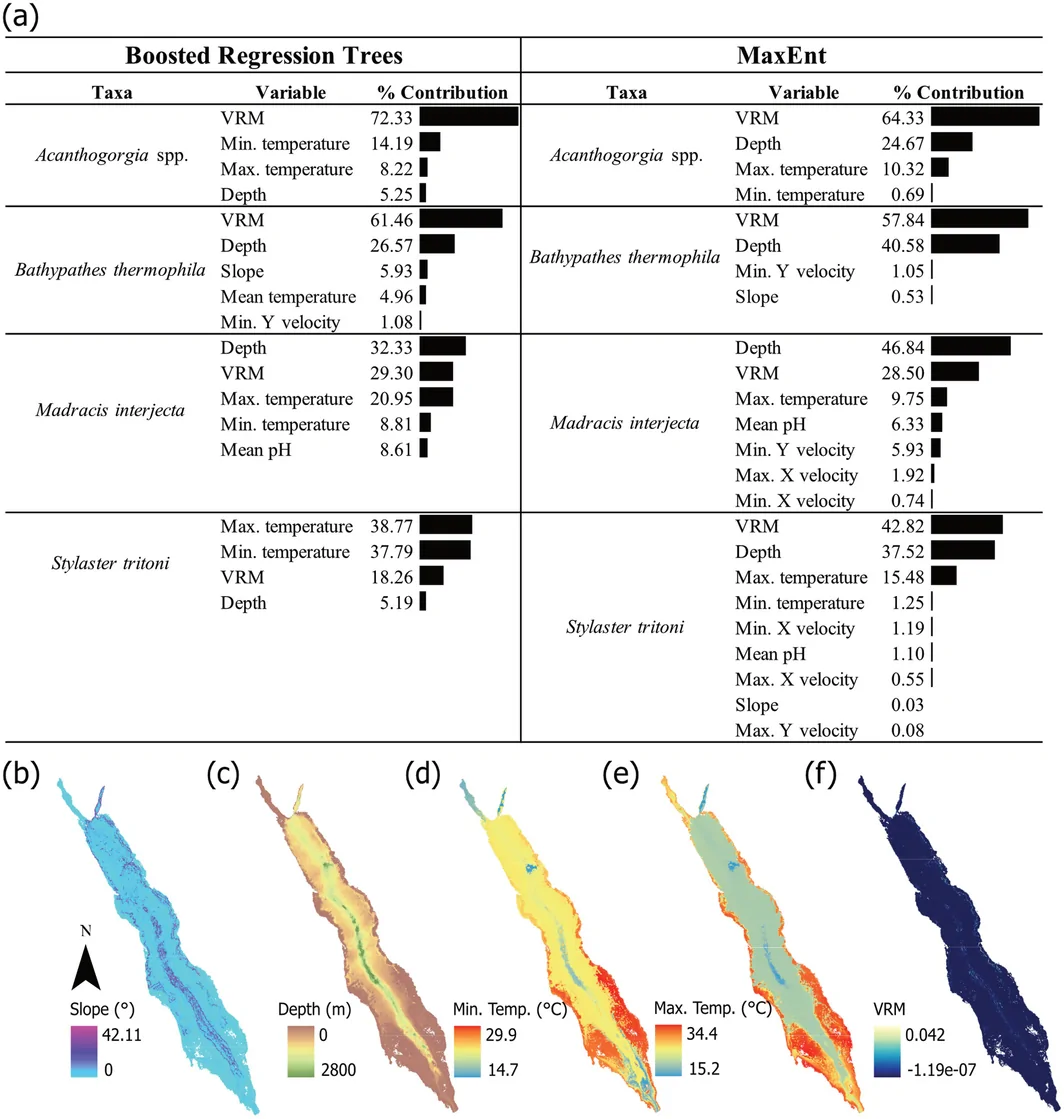
Relative contributions of the retained variables in the two modelling approaches for the four Red Sea coral taxa in this study (a), and rasters for each of the variables with a contribution over 10%, including slope (degrees) (b), depth (meters) (c), minimum temperature (degrees Celsius) (d), maximum temperature (degrees Celsius) (e) and Vector Ruggedness Measure (VRM; f). Map lines delineate study areas and do not necessarily depict accepted national boundaries.

### 3.4 Ecological Niche Model (ENM) Predictions Under Present Conditions

The suitable habitat of *Acanthogorgia* spp. were estimated to cover the greatest area across the Red Sea: 52,397 km^2^, equating to 14.8% of the total study area. This area covered a depth range from the surface to around 2500 m depth (Supplementary Figures 6 and 7), and was present in some parts of the central axis, particularly in the south (Figure 2). *B. thermophila* was estimated to cover around 10.0% of the area of the Red Sea, at 35,384 km^2^. This area was focused along the coast, with particular hotspots in the northern Red Sea, close to the Gulf of Aqaba and Suez, and in the southern Red Sea, north of the Dhalak Islands of Eritrea (Figure 2). The area suitable for *S. tritoni* was estimated at around 48,731 km^2^, equating to 13.7%. This species extended into a greater area in the Gulf of Aqaba, but also extended along the whole latitudinal extent of both coastlines, with slightly more coverage around the Farasan Bank on the Saudi Arabian coast (Figure 2). Finally, *M. interjecta* was estimated to cover the lowest area, around 29,317 km^2^ and 8.3% of the Red Sea. This taxon is restricted to the narrowest depth range, between 100 and 625 m depth, and the highest concentration of suitable areas was found in the northern Red Sea and Gulf of Aqaba (Figure 2). The suitable habitat for *Acanthogorgia* spp. occupied the most unique area, that is, area which was not estimated suitable for any other species (15,446 km^2^), while *B. thermophila* occupied the least (1244 km^2^) (Supplementary Figure 5). The environmental conditions for the suitable habitat for each coral taxa, and for the most important variables (Figure 1) are shown in Supplementary Figure 6.

**FIGURE 2:**
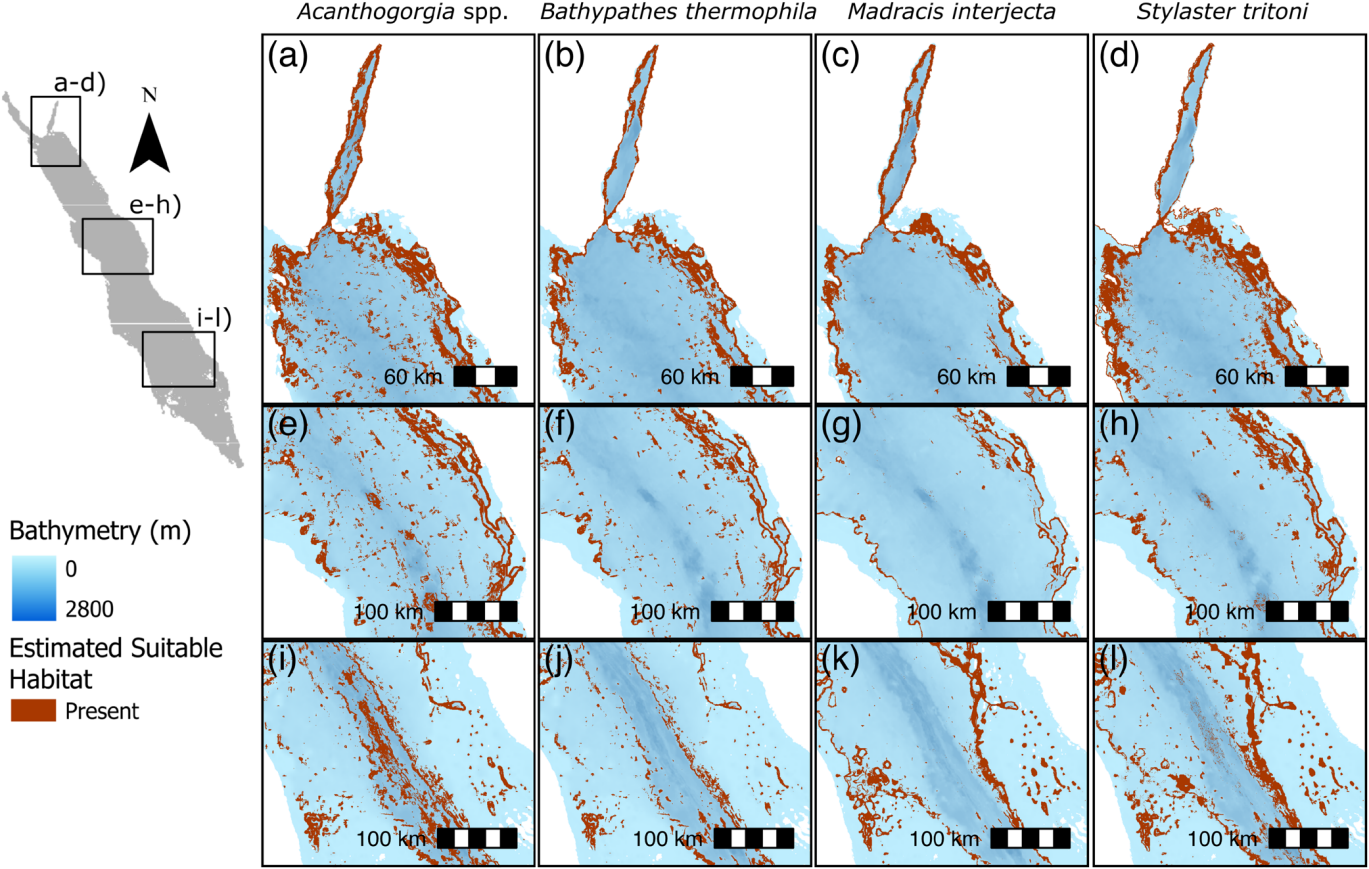
Estimated suitable habitat for each taxa based on ecological niche models. Areas over the threshold (selected to maximise the sum of specificity and sensitivity) are shown as estimated presences in dark red, with the depth (meters) shown in blue, for areas in the northern Red Sea (a–d), the central Red Sea (e–h) and the southern Red Sea (i–l). (a, e, and i) *Acanthogorgia* spp.; (b, f, and j) *Bathypathes thermophila*; (c, g, and k) *Madracis interjecta* ; and (d, h, and l) *Stylaster tritoni*. Map lines delineate study areas and do not necessarily depict accepted national boundaries.

### 3.5 Ecological Niche Model (ENM) Predictions Under Future Conditions

Species are not always able to shift to potentially suitable habitat due to dispersion barriers, so we analysed both the total suitable habitat available (absolute area), and the future suitable habitat that falls within the current range (the overlap). Under predicted future conditions, the amount of currently suitable area was reduced for every taxon (Figure 3), resulting in a small overlap in habitat. This loss is consistently greater under more severe SSPs. *B. thermophila* is an exception to this general trend, where the slight reduction in habitat was minimal, likely due to the small contribution of environmental variables to the models, resulting in future models that are largely built on geomorphometric variables (VRM, depth and slope) that will not significantly change under climate change. In all cases, the two most severe scenarios for 2090–2100 resulted in a potentially large area of current suitable habitat becoming unsuitable. This trend was worst in *M. interjecta* , which would lose over 45% of currently suitable habitat under SSP5‐8.5, dropping from 29,317 km^2^ to 15,893 km^2^. Under the two worst climate scenarios, this loss in suitable habitat was significant for *M. interjecta* (Wilcoxon rank‐sum test: W = 305, *p* = 0.003 for SSP3‐7.0, W = 309 *p* = 0.013 for SSP5‐8.5). Conversely, *B. thermophila* would lose the least amount of suitable habitat only around 2.3%, dropping from 35,384 km^2^ to 34,571 km^2^. Although areas that are currently suitable will become unavailable, other areas will become suitable for these corals, shown by the increase in absolute area of suitable habitat for *Acanthogorgia* spp. and *S. tritoni* (Figure 3).

**FIGURE 3:**
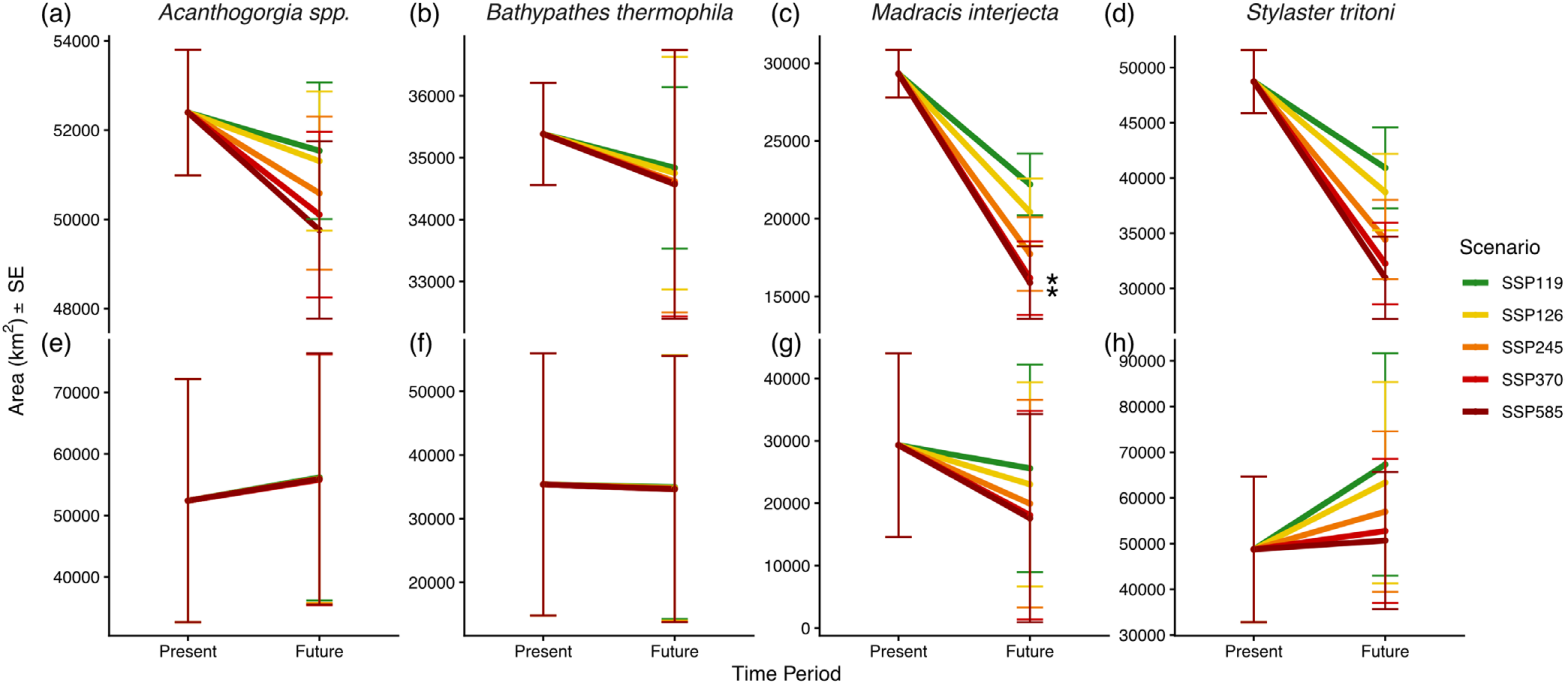
Change in area of suitable habitat for each coral taxon in Red Sea from the present to 2090–2100, under five shared socio‐economic pathways (SSPs), ranging from more optimistic (green) to more severe (red). The top row (a–d) shows the amount of area (km^2^) that is currently suitable, on the left, and the proportion of that area that will remain suitable on the right (the overlap or refuge). This does not take range expansion into account. The bottom row (e–h) shows the absolute area that is suitable now and in the future, taking into account the potential for range expansion and shifts. Error bars are standard error. Significance between time points is calculated by Wilcoxon rank‐sum tests and indicated by asterisks to the right of the future time points: * < 0.05.

Due to the similarities between all future scenarios for most species, a moderate scenario, SSP2‐4.5, was used as a focus to evaluate the amount of suitable habitat that was lost, gained, or retained between the present and 2090–2100. The patterns are visibly different between species (Figures 4, 5, 6, 7). *Acanthogorgia* spp. gains habitat in adjacent areas, but also along the central axis, large areas of suitable habitat are retained along the depth contours of the Red Sea coastline (Figure 4), while small, shallower areas appear to be lost. *B. thermophila* remains largely unchanged and is able to retain most of its suitable habitat, neither gaining nor losing large areas (Figure 5). Large areas of *M. interjecta* are lost, particularly in the northern and southern Red Sea, with some retained habitat, but very little gained (Figure 6). In contrast, *S. tritoni* will retain some important areas of habitat in the northern Red Sea and in the Southwest, close to the Dhalak Islands. Some gained habitat is seen in deeper areas, particularly the central southern Red Sea (Figure 7).

**FIGURE 4:**
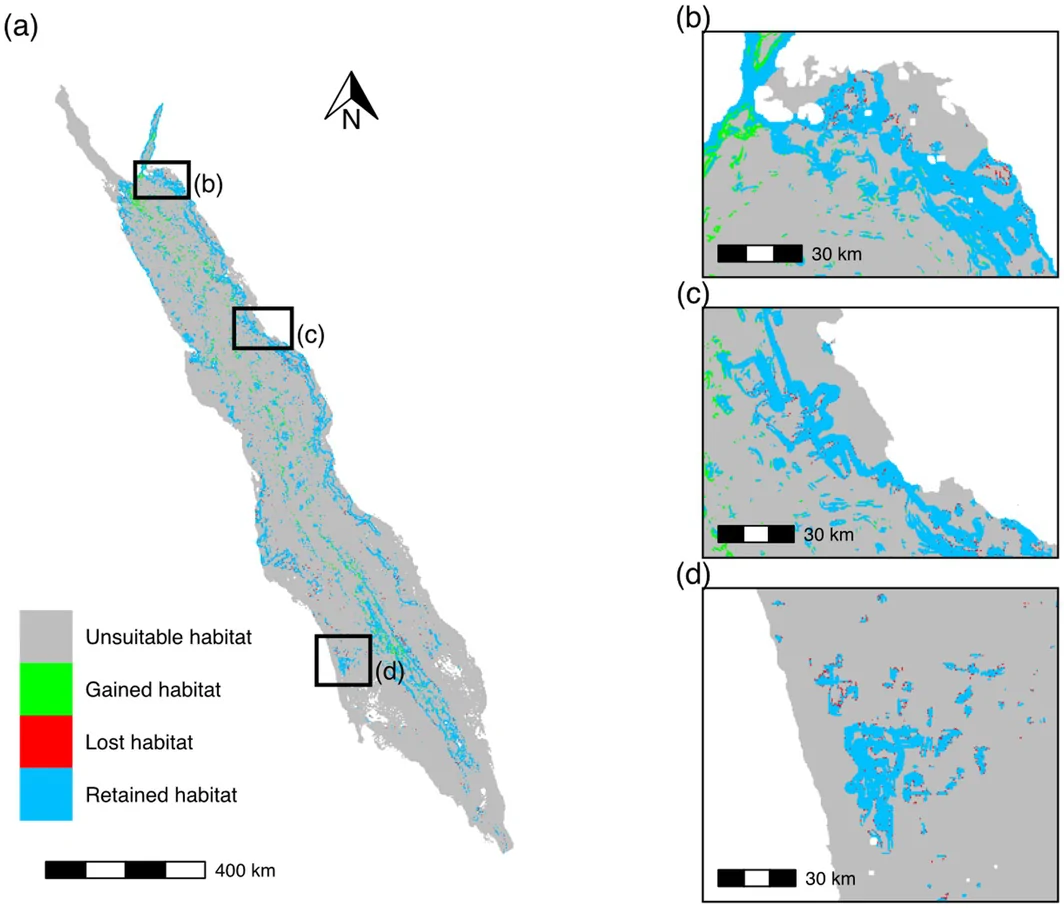
The gain, loss and retention of suitable habitat by *Acanthogorgia* spp. in the Red Sea between the present day and 2090–2100 based on shared socio‐economic pathways (SSP) 2–4.5. Newly gained potential habitat is shown in green, lost habitat in red, and habitat which is suitable both now and in the future is shown in blue. The whole Red Sea is shown in (a), with close ups for key areas in the north in (b), along the central‐north Saudi Arabian coast in (c) and along the south‐western coast in (d). Map lines delineate study areas and do not necessarily depict accepted national boundaries.

**FIGURE 5:**
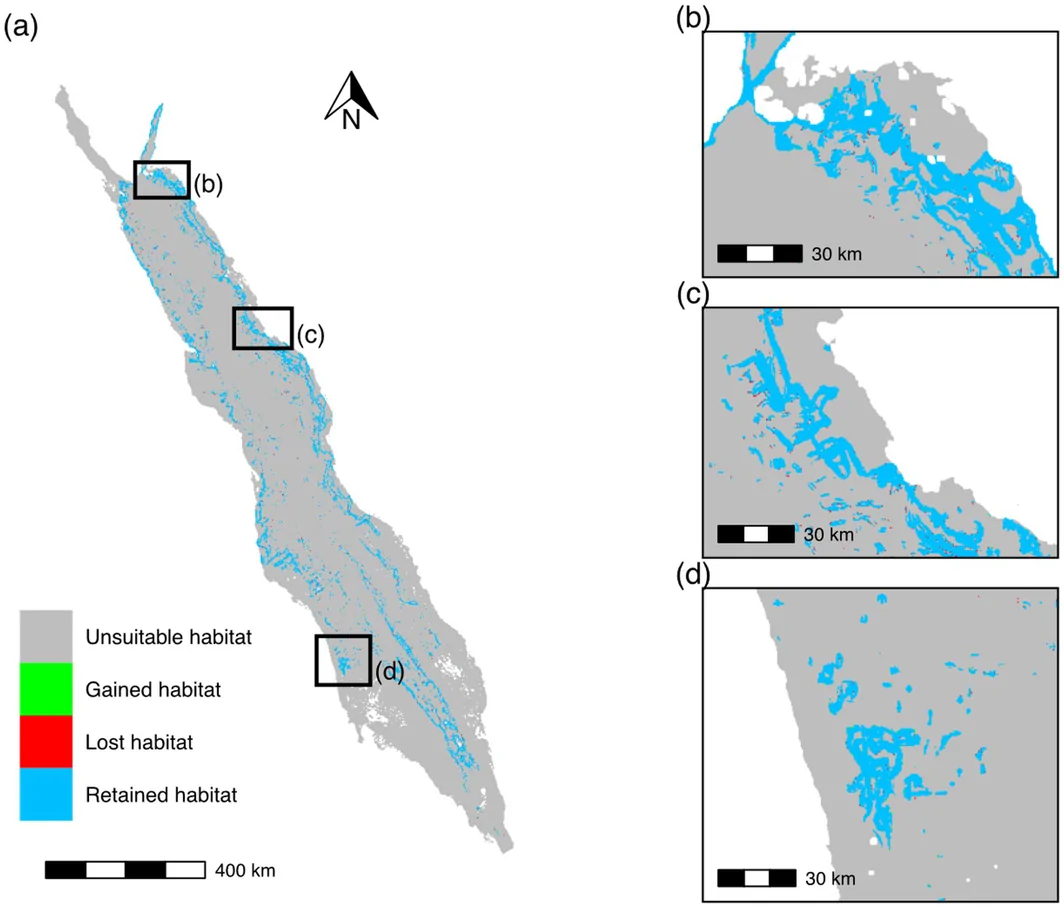
The gain, loss and retention of suitable habitat by *Bathypathes thermophila* in the Red Sea between the present day and 2090–2100 based on shared socio‐economic pathways (SSP) 2–4.5. Newly gained potential habitat is shown in green, lost habitat in red, and habitat which is suitable both now and in the future is shown in blue. The whole Red Sea is shown in (a), with close ups for key areas in the north in (b), along the central‐north Saudi Arabian coast in (c) and along the south‐western coast in (d). Map lines delineate study areas and do not necessarily depict accepted national boundaries.

**FIGURE 6:**
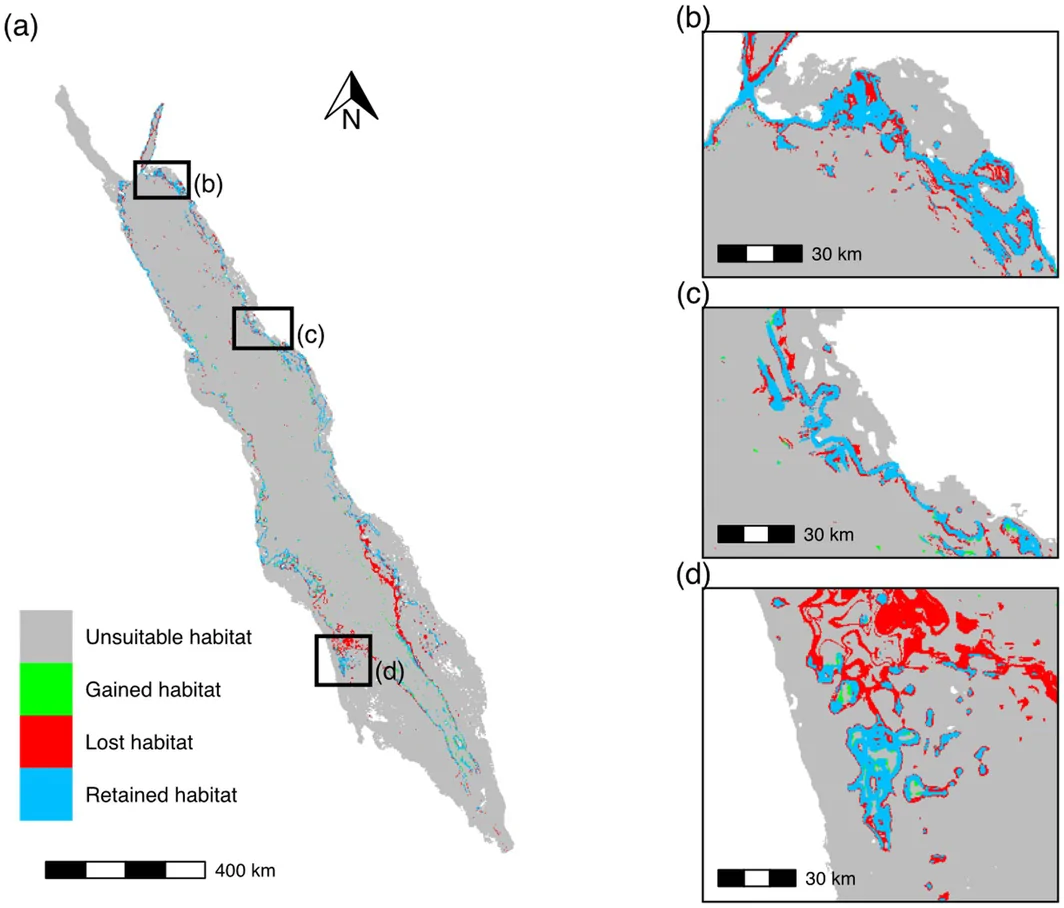
The gain, loss and retention of suitable habitat by *Madracis interjecta* in the Red Sea between the present day and 2090–2100 based on shared socio‐economic pathways (SSP) 2–4.5. Newly gained potential habitat is shown in green, lost habitat in red, and habitat which is suitable both now and in the future is shown in blue. The whole Red Sea is shown in (a), with close ups for key areas in the north in (b), along the central‐north Saudi Arabian coast in (c) and along the south‐western coast in (d). Map lines delineate study areas and do not necessarily depict accepted national boundaries.

**FIGURE 7:**
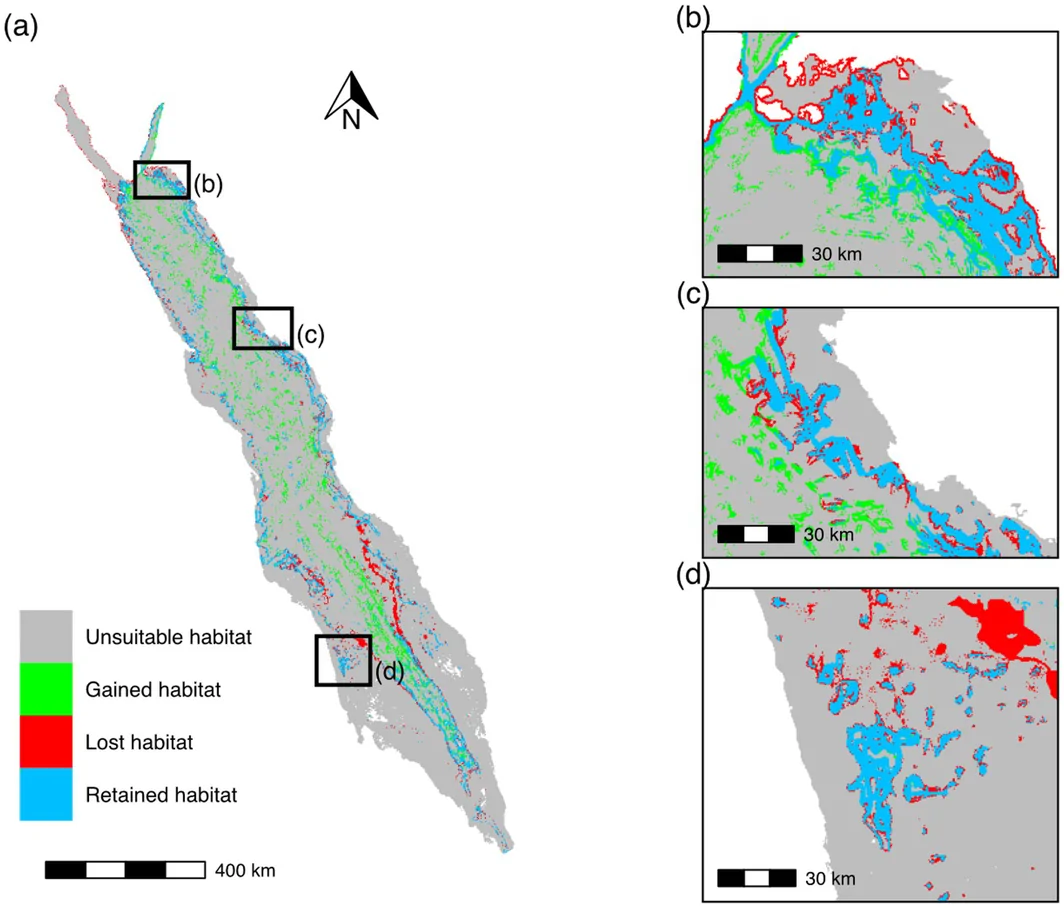
The gain, loss and retention of suitable habitat by *Stylaster tritoni* in the Red Sea between the present day and 2090–2100 based on shared socio‐economic pathways (SSP) 2–4.5. Newly gained potential habitat is shown in green, lost habitat in red, and habitat which is suitable both now and in the future is shown in blue. The whole Red Sea is shown in (a), with close ups for key areas in the north in (b), along the central‐north Saudi Arabian coast in (c) and along the south‐western coast in (d). Map lines delineate study areas and do not necessarily depict accepted national boundaries.

The average temperature in which suitable habitat is found increases for all corals, indicating a potential need for adaptation. In addition to an increase in temperature, the area of suitable habitat increases in average depth under future conditions, somewhat for *Acanthogorgia* spp. and *M. interjecta* , but most notably for *S. tritoni*, which increases from a mean depth of 424 m to 675 m under SSP5‐8.5. For both temperature and depth, there is an increasing shift, either towards warmer temperatures or deeper waters, under increasingly severe scenarios (Supplementary Figures 7 and 8). Even where species are shifting their habitat to deeper waters, they are still experiencing increasing average temperatures, highlighting the severity of changes expected to happen in the deep sea by the end of the century.

Some areas were suitable for all four taxa, covering 16,960 km^2^ across the Red Sea, which may indicate the presence of biodiversity hotspots. These hotspots were found in the northern Red Sea, on both the East and West coasts of the Red Sea, south of Umluj in Saudi Arabia, the corresponding area at the same latitude on the western coast (around Berenice Troglodytica in Egypt), and around the Farasan Bank (Saudi Arabia) and north of the Dhalak Islands (Eritrea) in the southern Red Sea (Figure 8a). The amount of area that is suitable for two or three taxa may increase slightly or remain relatively stable, while the area that is suitable for just one taxon is likely to drop under all scenarios, and the area suitable for all four taxa could increase under less severe scenarios, but is likely to drop under the more severe scenarios (Figure 8b).

**FIGURE 8:**
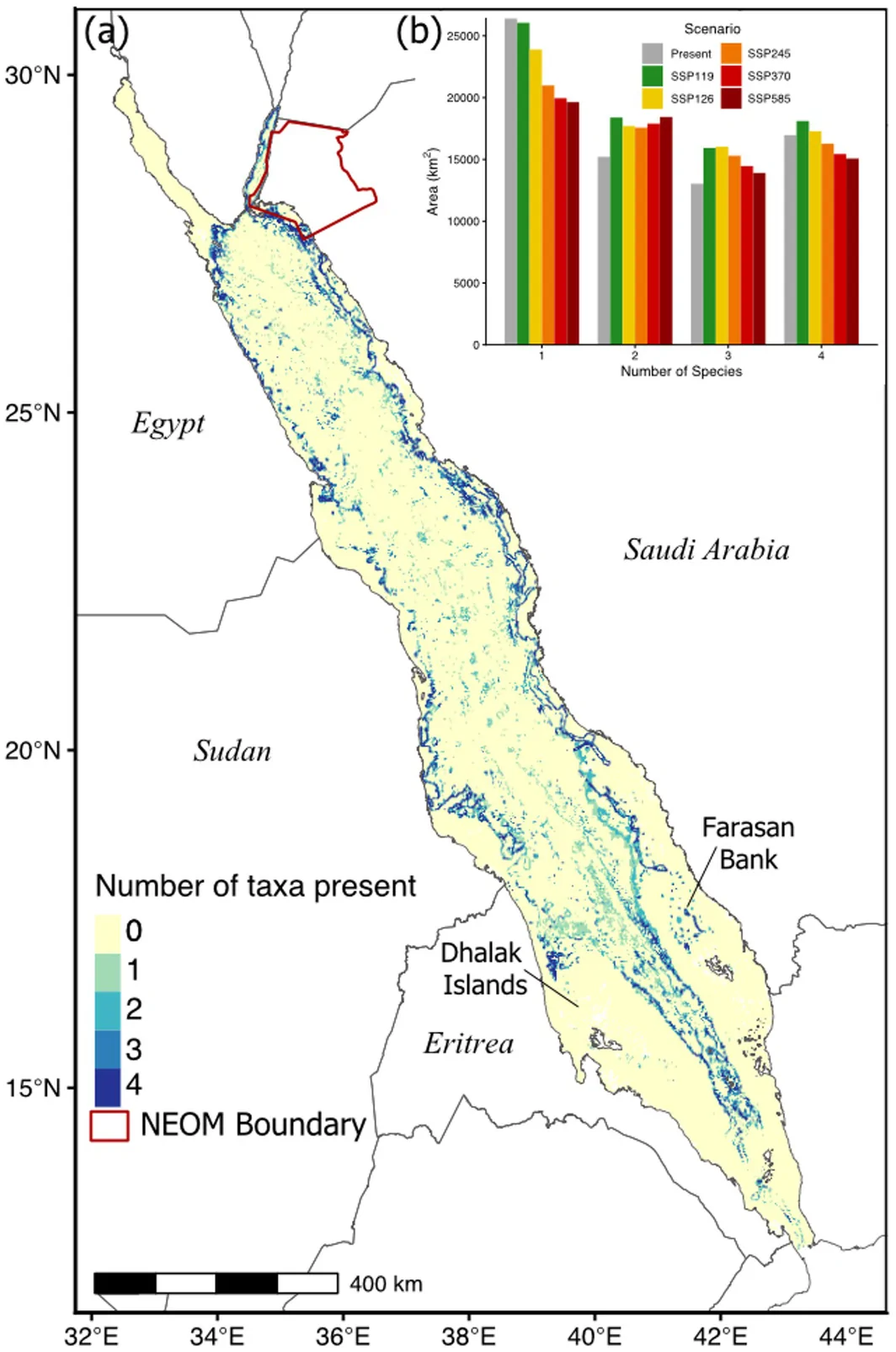
Potential biodiversity hotspots for corals across the Red Sea, based on the number of the four coral taxa in this study modelled to be present in the area (a). Darker colours indicate that the area is suitable for more coral taxa. The estimated area based on ecological niche models that is suitable for 1, 2, 3 and 4 coral taxa under present and future scenarios is shown in (b). Current area (km^2^) is shown in grey, and then shared socio‐economic pathway (SSP) scenarios are shown in a scale from green to dark red, going from most optimistic to most severe. Map lines delineate study areas and do not necessarily depict accepted national boundaries.

## 4 Discussion

Understanding the distribution of current and future habitat is essential information to effectively protect species against growing threats (Cayuela et al. 2009). Our results have demonstrated that under all estimated future emissions scenarios, significant amounts of the current habitat for two of the four studied widespread mesophotic and deep Red Sea coral taxa‐ *Acanthogorgia* spp., *Stylaster tritoni*, *Madracis interjecta* , and *Bathypathes thermophila*‐ will become unsuitable (Figures 4, 5, 6, 7). The models generated here predict that corals may lose up to 45% of their current habitat, forcing them to either expand their ranges, adapt to new conditions, or face significant reductions in their populations.

Between four and nine variables were used in the final models; of these, VRM and depth emerged as drivers of the distribution of all four coral taxa (Figure 1), while temperature was a driver in seven of the eight models. These key drivers are in agreement with several other published studies (Buhl‐Mortensen et al. 2015; Chimienti et al. 2019; Davies and Guinotte 2011; Georgian et al. 2014; Guinotte and Davies 2014; Lauria et al. 2017, 2021; Morato et al. 2020; Nolan et al. 2024; Tittensor et al. 2010). From the correlations tested here (Supplementary Figure 9a), it is evident that both of these variables are strongly correlated to other environmental factors. For example, depth is correlated not only to average temperature (Pearson's *r* = 0.7 in our dataset) but also to light levels (photosynthetically active radiation, PAR) (Aksnes et al. 2017), salinity (Emery and Dewar 1982), oxygen concentration, nutrient levels, and carbon concentrations (Koop et al. 1990). These correlations are not consistent among regions around the world and may be stronger or weaker depending on other environmental factors. Resultantly, temperature or depth may have high contributions as they are also acting as proxies for other variables.

Oxygen concentration was included in the original set of predictor variables, but following trials, it was found not to contribute to any models and was removed from further analysis. It was therefore also not used in the future models, despite predicted changes to oxygen levels in the deep Red Sea by our Bio‐Oracle results. Corals are usually absent from low oxygen zones, including the southwestern African margin, where a drop in regional oxygen concentration 5000 years ago was estimated to coincide with the loss of live coral cover in the region (Hanz et al. 2019). This is supported by laboratory studies, where the lower concentration of oxygen in which *Desmophyllum pertusum* (Linnaeus, 1758) was able to regulate respiration rates corresponded with the lower limit of natural distribution, leading the authors to conclude that the distribution of this species off the coast of Scotland was controlled by the oxygen concentration of the water (Dodds et al. 2007). In contrast, a zone of low oxygen above the benthos may be beneficial to benthic fauna, as Wishner et al. (1990) found higher levels of organic matter being deposited to the seafloor. Additionally, in the Azores, *Acanthogorgia* spp. was found to live mostly in and above the oxygen minimum zone, tolerating extreme conditions (Taranto et al. 2023). It has been suggested that corals in the low‐oxygen conditions of the Red Sea may have developed adaptations to survive, including reduced metabolic and calcification rates (Roder et al. 2013). This may be the case of the Red Sea endemic species *S. tritoni*, which was found in high abundances in extremely low oxygen conditions in the southern Red Sea (Klein et al. 2025), and perhaps the reason that oxygen was not selected as a driver of the distribution in any of our focal taxa. This discussion is speculative and further experimentation is required to accurately validate the tolerance of Red Sea species to anomalous oxygen concentrations.

### 4.1 Comparison of Suitable Habitat Now and Under Climate Change Scenarios

Differences in the estimated future distribution patterns of the four coral taxa indicate that the physiological responses of these taxa to changing climate are different. Suitable habitat for *Acanthogorgia* spp. is estimated to cover almost 12% of the Red Sea currently, across a broad depth gradient. The depth‐generalist nature of this genus has been documented in the Red Sea (Macrina et al. 2024, 2025), the Mediterranean (Angiolillo and Canese 2018), and Indo‐Pacific (Benayahu et al. 2019; Rowley et al. 2019), and supports this finding. Under climate change scenarios, the average depth of areas of suitable habitat for *Acanthogorgia* spp. shifts to deeper waters by 55 m (Supplementary Figure 7), resulting from the gain of habitat along the central axis, and small losses among the shallowest areas (Figure 4). This is due to projected temperature increases in the deep sea, increasing available habitat that may support the physiological requirements of this coral. Due to the broad depth range, this shift may be possible from both a physiological and a dispersal perspective. However, we are considering all Red Sea species of the genus *Acanthogorgia* here, which do not all share the same depth distribution. While some species may be able to shift to deeper waters, or some existing deep‐sea species may be able to expand their range, there also may be species that could experience local extinctions in shallow waters; therefore, both vulnerabilities and strengths of these corals could be masked through the genus‐level model. This response may be similar to that experienced by the hydrocoral *Millepora intricate* Milne Edwards, 1860 in the eastern Pacific (Smith et al. 2014), although here we are considering a much greater depth range (100 s m depth vs. 11 m in Smith et al. (2014)). In combination, maximum and minimum temperature contributed 22.41% and 11.01% to the BRT and MaxEnt models respectively for *Acanthogorgia* spp. (Figure 1a), highlighting why this genus is able to shift to slightly warmer temperatures, but not to dramatically different conditions. This is in agreement with manipulative studies that have found reductions in metabolic activity of shallow and mesophotic Mediterranean octocorals under increased temperatures (Previati et al. 2010), and the suggestion that high temperatures control the upper depth distribution of octocorals in the northwest Atlantic (Mortensen and Buhl‐Mortensen 2004).

Suitable habitat for *B. thermophila* is mostly distributed close to the coastline, with very little occurring in the central Red Sea. *Bathypathes* sp. has been considered a deep specialist in the Red Sea (Vicario et al. 2024), evident also in the suitable habitat predicted here, although elsewhere this genus has been found in much shallower waters (Bo et al. 2019). *B. thermophila* is able to maintain almost all of its current habitat (Figure 5), with no significant differences between present and future climate scenarios (Figure 3). Due to the high contribution of depth to the model for *B. thermophila* (Figure 1), there is little change in the average depth (502 m) under any future climate scenario (Supplementary Figure 7g–l). If true, *B. thermophila* will need to tolerate warmer temperatures, increasing by almost 2°C on average by 2090–2100 under the most severe scenario. In contrast, other studies have found black corals to have a narrow thermal tolerance; for example, Godefroid et al. (2022) suggested the black coral *Stichopathes* sp. to have low acclimatisation potential, although the thermal optimum of this mesophotic black coral is > 28°C, which is higher than the average temperature of the *B. thermophila* temperature estimated in the Red Sea (Supplementary Figure 8).

*Madracis interjecta* exhibits the smallest current distribution of all four coral taxa, with suitable habitat covering only 29,317 km^2^, equivalent to 6.7% of the area of the Red Sea. This is a larger proportion of suitable area than in the northern Red Sea, where only 0.2% of the whole study area was suitable (Nolan et al. 2025). As well as differences in methodologies, this may be a factor of resolution, as the previous study was conducted at 30 m resolution. *M. interjecta* is projected to lost huge amounts of its habitat, with only very small patches potentially becoming suitable (Figure 6), suggesting dispersal to new areas will not be possible for the species. Both depth and temperature have high contributions to the model for *M. interjecta* , and the average values of future suitable habitat changes for both‐ *M. interjecta* is estimated to move to slightly deeper waters (73 m; Supplementary Figure 7), but the shift to deeper ranges would not be sufficient to avoid an increase in thermal conditions, and average temperatures may still increase by up to 2.3°C (Supplementary Figure 8). Although there is very limited research on the thermal tolerance of deep or mesophotic Scleractinia, there are some studies that have shown that adult colonies are able to withstand acute high temperatures (Brooke et al. 2013; Naumann et al. 2013), but survival decreases with time, and such high temperatures may compromise the survival of larvae (e.g., in shallow water corals; Edmunds 2004). Other have estimated a reduction in habitat for mesophotic and deep‐sea Scleractinia, including other reef‐forming species studies from New Zealand and the North Sea (Anderson et al. 2022; Morato et al. 2020).

Finally, the potential habitat of the stylasterid *S. tritoni* is also limited across the Red Sea, but occupies larger patches around the coastline, usually on ridges and seamounts where hard substrate is available (Cairns 1992). Stylasterids are found in dense aggregations globally, but the depth distribution of these aggregations varies considerably. For example, within the stylasterid genus *Errina* Gray, 1835, aggregations may occur between 80 and 100 m in the Mediterranean (Salvati et al. 2010), around 450 m in the Azores (Wisshak et al. 2009), and in much shallower waters of 30–50 m around New Zealand (Miller et al. 2003). Under future climate change scenarios in the Red Sea, *S. tritoni* exhibits the potential for the loss of large amounts of its current habitat (Figure 3d), coupled with potential gains in habitat in deeper waters, including the central southern Red Sea where temperatures become suitable (Figure 7). *S. tritoni* exhibits a potential pattern of increased overall habitat under SSP1‐1.9 and SSP1‐2.6, which is comparable to the results of Anderson et al. (2022), where a 100% increase in suitable area was estimated for *Stylaster* spp., although the potential for dispersal capability to these areas remains. The average depth for habitat suitable for *S. tritoni* increased considerably by up to 251 m between the present day and the future, with the greatest increase in average depth occurring under the most severe SSP (Supplementary Figure 7s–x). Temperature variables in combination contributed over 75% to the BRT model for *S. tritoni*, highlighting the potentially narrow thermal niche of this species. However, for the MaxEnt model, temperature variables contributed only 16.78%, indicating the largest discrepancy between the two models for any species. *S. tritoni* has been shown to survive in other extreme environmental conditions, such as low oxygen concentrations in depressions in the southern Red Sea (Klein et al. 2025), but there is little evidence of strong adaptation potential to high temperatures, although Stylasteridae were identified from large thermal ranges in the Caribbean (Auscavitch et al. 2020). *S. tritoni* is estimated to have a broad depth range under current conditions, and may be able to disperse to the areas that will be environmentally suitable in future, for example, within the required thermal range, and with a suitable substrate.

There is a documented poleward trend of shifting species ranges under climate change (e.g., Hastings et al. 2020; Simpson et al. 2011). Strong latitudinal gradients in the Red Sea (Berumen et al. 2019) might suggest a potential for similar shifts in the Red Sea. The average latitudes of *M. interjecta* increases under more moderate future scenarios, indicating a slight shift into cooler waters in the north (Supplementary Figure 10). *B. thermophila* and *Acanthogorgia* spp. do not change in their average latitudes (Supplementary Figure 10), while *S. tritoni* shows a variable pattern, where the latitudes are on average lower in more severe future scenarios (Supplementary Figure 10).

### 4.2 Biodiversity Hotspots and Management Suggestions

Here we have identified areas where all four study taxa are present. The presence of these diverse species indicates that these areas could harbour greater biodiversity and may be considered hotspots. Although these hotspots are represented by just four taxa, they are taxonomically diverse among Cnidaria, found in different conditions in the Red Sea (Chimienti et al. 2022; Klein et al. 2025; Macrina et al. 2024; Maggioni et al. 2022; Nolan et al. 2025). The identified hotspots were focused along parts of the coastline (Figure 8), and correspond with areas that have previously been studied and found to be important ecological areas, including areas in the northern Red Sea, partially located within the jurisdiction of NEOM, where, for example, important deep coral frameworks have been identified (Nolan et al. 2024, 2025). NEOM is a major development for tourism, with ambitious environmental conservation goals (neom.org). This area was also the most intensively surveyed area in our dataset, covered by all three of the OceanX expeditions, and is of particular interest for management due to its sustainability goals. Knowledge presented here regarding the persistence of these hotspots in future, despite some habitat loss, is crucial to effective spatial planning (Supplementary Figure 11), as management needs to include areas that are able to accommodate both the present and future ranges of species under climate change (Brito‐Morales et al. 2020). The regions in the southern Red Sea, around the Farasan Bank and north of the Dhalak Islands are also key areas that are able to support and maintain habitat for all four species that should be targeted for conservation (Figure 8). While the Farasan Bank was supported by in situ ground truth points, the identification of the area north of the Dhalak Islands as a potential biodiversity hotspot is based solely on results of the models. The identification of these areas as potential habitat for four diverse coral taxa suggests their potential importance for future research and ground truthing. These three hotspots (NEOM, Farasan Bank, and Dhalak Islands) have been identified from visual and quantitative data, but a full conservation prioritisation analysis would be beneficial to identify whether these areas are cost effective for management (Howell 2010). Additionally, these areas represent regions where the high biodiversity remains relatively stable under changing conditions, but alternative areas across the Red Sea may be additionally important to protect as part of a dynamic Marine Protected Area (MPA) network.

### 4.3 Uncertainty and Limitations of This Study

Ecological niche modelling studies provide invaluable information on the distribution of species and assemblages now and in the future, for areas that would otherwise be data deficient (Guinan et al. 2009). However, they are not without limitations and inherent uncertainties. Previous studies have reported uncertainties in Red Sea temperatures, particularly for projected climate changes (Agulles et al. 2020, 2021). In the present study, we improved the resolution of Bio‐Oracle data from 5 km to 400 m across the Red Sea, increasing the quality through the addition of in situ data. To further improve the understanding of the distributions of these corals, high‐resolution (< 200 m resolution) bathymetric data is required (Ross et al. 2015). High‐resolution data also needs to be paired with a robust and even sampling regime (Hirzel and Guisan 2002). In the present study, data for coral presences (video transects) and environmental data (CTD casts and bathymetry) were almost all collected in the Saudi Arabian Red Sea, with only two data presence points from Egypt. Additionally, limited high‐resolution bathymetry was obtained from outside Saudi Arabia waters. High‐ and low‐resolution bathymetry datasets were merged with a buffer where data was interpolated to avoid steps in the data. Although this provided a smoother raster, interpolation can introduce errors to the data. We do not believe this to be a problem here due to the small area over which data was interpolated (800 m buffer; Supplementary Figure 3a). This highlights the higher confidence in our results from the Eastern side of the Red Sea, and the need for greater exploration of the mesophotic and deep benthic areas on the western side of the Red Sea, including improved data sharing between Red Sea stakeholders. Although using inferred absences from well distributed survey points is widely considered the gold standard in habitat suitability modelling (ICES 2021), our dataset was not appropriate for this modelling (Supplementary Figure 2), and we aimed to mitigate the effects of uneven sampling through the selection of pseudo‐absences from all climate clusters.

An additional limitation was the lack of a variable to describe seafloor composition. Two of our four taxa (*Acanthogorgia* spp. and *S. tritoni*) require a hard substrate for settlement (e.g., Cairns 1991; Mortensen and Buhl‐Mortensen 2004), precluding their occurrence in the expansive deep abyssal plains of the central Red Sea. While *Madracis* spp. has been recorded on sandy bottoms in Colombia (Cedeño‐Posso et al. 2022), it is also more regularly associated with rocky substrate (e.g., Auscavitch et al. 2020; Stefanoudis et al. 2019). The incorporation of backscatter could provide some information on the seafloor composition, but was not available across our whole study area, however the geomorphometric variables VRM and slope can act as proxies for the presence of hard substrate.

Standard deviation (SD) among model runs varied both spatially and between taxa (Supplementary Figures 12 and 13), with generally higher uncertainty in areas estimated to be more suitable for the taxa. Standard deviation was higher among BRT models than MaxEnt, particularly for *S. tritoni*, which had the most widespread areas of uncertainty for the BRT models, and lower agreement between models (Supplementary Figure 29). This included some of the highest SD values in the southern Red Sea, close to the Bab‐el‐Mandeb Strait, and in the northern Red Sea, incorporating the Gulf of Suez. These are areas with limited in situ sampling points, where we have relied on remotely sensed data, showing the value of active sampling in increasing confidence in results (Wisz et al. 2008). Uncertainty is higher outside of Saudi Arabian waters, particularly for *S. tritoni*, which highlights the importance of international data sharing across the Red Sea. The inclusion of data collected on the western side of the Red Sea would likely reduce this uncertainty. This is of particular importance as this data, including the identification of biodiversity hotspots, is valuable for management, and high certainty in the results may lead to better adoption of management actions. Further uncertainty also comes from climate projections, as they are based on numerous assumptions (Zhang and Chen 2021), but here we chose to carry out models across five different well‐documented scenarios (Moss et al. 2010), selecting a moderate scenario to highlight in the main text, due to the lack of significant differences between most scenarios.

It has been shown that ecological niche models may provide a better estimation of habitat gained than lost, perhaps due to the unknown thermal tolerance of corals and their ability to adapt (Schwartz 2012). However, areas of gained habitat are based on environmental conditions and may not take into account the potential limitations of dispersal into these areas. Here, many of the gained areas are in deeper waters that are isolated from the current range, particularly for *Acanthogorgia* spp. and *S. tritoni*, and may not be accessible (e.g., along the central axis). The estimation of taxa moving into deeper waters may also depend on biological factors not considered in these models, including potential competition with existing benthic fauna at those depths (HilleRisLambers et al. 2013).

### 4.4 Areas of Future Research

This research provides an important starting point for substantial future research on mesophotic and deep‐sea corals in the Red Sea, both now and in the future. One important advancement on the current research would be the development of biomass models (e.g., Gouvêa et al. 2020, 2022). Here, we have improved the understanding of where species may be able to survive, but both *S. tritoni* and *B. thermophila* were seen in larger aggregations: *S. tritoni* in the southern Red Sea (Klein et al. 2025), and *B. thermophila* in the northern Red Sea (Chimienti et al. 2022). The presence of all four taxa in these localised areas might indicate optimal conditions, and further exploration is warranted. For example, biomass models would highlight areas where these aggregations can form, and further elucidate where environmental conditions are optimal. In turn, this would assist in our understanding of the physiological requirements of these corals.

To fully understand the potential future habitat of a species, we need to understand its physiological tolerances (Bozinovic and Naya 2014). These models assume that the taxa are living close to their environmental limits, but this is not always the case. Experimental studies can complement field surveys and statistical models to understand these limits. Additionally, a better understanding of the larval stage of deep Red Sea species would facilitate a larval dispersal model that would remove speculation on whether or not species are able to disperse to newly suitable areas.

### 4.5 Conclusions

We presented estimates of both current and future cover of four important coral taxa across a large spatial scale, the whole Red Sea basin, combining data from multiple expeditions as well as deposited online material. With additional ground‐truthing records, we improved both the accuracy and the spatial resolution of Red Sea environmental models, allowing a high‐quality dataset that can be used not only to directly model the spatial distribution of these species, but that can also be used as a starting point for a number of other studies.

ENMs are extremely valuable for ecosystem‐based management (Grumbine 1994; Slocombe 1998), and the importance of collecting and processing data from the deep sea continues to grow, as we understand more about these ecosystems. Such studies have been proposed to aid in the design of MPAs for conservation of resources in the deep sea (Howell 2010). The present study provides the fundamental information relevant to the establishment of a well‐informed, long‐term and internationally recognised network of protected areas in Red Sea waters.

## Author Contributions

**Megan K. B. Nolan:** conceptualization, data curation, formal analysis, investigation, methodology, visualization, writing – original draft. **Jorge Assis:** formal analysis, data curation, investigation, methodology, writing – review and editing. **Fabio Marchese:** conceptualization, data curation, formal analysis, methodology, supervision, writing – review and editing. **Natalie Dunn:** data curation, writing – review and editing. **Marta Ezeta Watts:** data curation, writing – review and editing. **Pauline Falkenberg:** data curation. **Nico Augustin:** funding acquisition, resources, project administration, writing – review and editing. **Thomas Lüdmann:** funding acquisition, project administration, resources, writing – review and editing. **Froukje M. van der Zwan:** funding acquisition, project administration, resources, writing – review and editing. **Hildegard Westphal:** funding acquisition, project administration, resources, writing – review and editing. **Mohammed Qurban:** funding acquisition, project administration, resources. **Vincent Pieribone:** funding acquisition, project administration, resources. **Carlos M. Duarte:** funding acquisition, project administration, resources, writing – review and editing. **Francesca Benzoni:** funding acquisition, project administration, resources, writing – review and editing, supervision, conceptualization, data curation.

## Funding

This project was supported by funding from KAUST (FCC/1/1973‐50‐01 and baseline funds BAS/1/1090‐01‐01 awarded to F.B.). This research also received Portuguese national funds awarded to J.A. from FCT—Foundation for Science and Technology through projects UIDB/04326/2025 (https://doi.org/10.54499/UIDB/04326/2025), UIDP/PRR/04326/2025 (https://doi.org/10.54499/UIDP/PRR/04326/2025), LA/P/0101/2020 (https://doi.org/10.54499/LA/P/0101/2020) and the Scientific Employment Stimulus 2022.00861.CEECIND (https://doi.org/10.54499/2022.00861.CEECIND/CP1729/CT0003). The research expeditions onboard the R/V OceanXplorer were funded by NEOM, NCW and OceanX. The research cruises Meteor M193 and M194 were funded by the Deutsche Forschungsgemeinschaft (DFG, German Research Foundation) awarded to H.W. and T.L., and to N.A. and F.M.v.d.Z. respectively. Research expedition R/V Aegaeo KRSE5 was funded by the KAUST VPR office.

## Conflicts of Interest

The authors declare no conflicts of interest.

## Supporting information


**Supplementary Figure 1.** The location of Remotely Operated Vehicle (ROV) and submersible dives from all six expeditions that contributed data to this analysis. The expedition names correspond to those in Table 1. RSDE is the Red Sea Decade of Exploration Expedition, RC is the Relationship Cultivation Mission, KRSE is the KAUST Red Sea Expedition.
**Supplementary Figure 2**. Differences in spatial and environmental representation by inferred absences from survey points and generated pseudo‐absences based on a trial with *Acanthogorgia* spp.; (a) spatial distribution in the Red Sea of inferred absences and presences for *Acanthogorgia* spp.; (b) spatial distribution in the Red Sea of pseudoabsences and presences for *Acanthogorgia* spp.; (c) a PCA comparing the environmental space which inferred absences (pink) and pseudo‐absences (blue) occupy; and (d) boxplots showing the representation of the eight environmental variables considered in the *Acanthogorgia* spp. model by inferred absences (pink) and pseudo‐absences (blue).
**Supplementary Figure 3**. The location of acoustic and oceanographic data collection across the Red Sea; (a) the areas of bathymetry collected in the Red Sea, with high resolution bathymetry (processed to 30 m) in orange and low resolution bathymetry (at 400 m) in blue; (b) the locations of CTD casts are shown in blue.
**Supplementary Figure 4**. The difference between 5 km Bio‐Oracle rasters across the Red Sea (a–c) and 400 m reprocessed rasters (d–f) for temperature (a, d), salinity (b, e) and oxygen concentration (c, f).
**Supplementary Figure 5**. The areas of overlap in estimated suitable habitat between the four coral taxa (*Acanthogorgia* spp., *Bathypathes thermophila*, *Madracis interjecta* and *Stylaster tritoni*) across the Red Sea and under present environmental conditions. Area where habitat is not predicted to be suitable for any species is not shown.
**Supplementary Figure 6**. The environmental conditions in which four coral taxa (*Acanthogorgia* spp., *Bathypathes thermophila*, *Madracis interjecta* and *Stylaster tritoni*) were observed in the Red Sea. The five variables shown here (depth, slope, minimum temperature, maximum temperature and vector ruggedness measure) are the most commonly selected and most important in the models. Minimum and maximum temperatures refer to the seawater temperature at the seafloor. The boxplots show the interquartile range as boxes, with whisker lines extending to 1.5 times the interquartile range. The central line is the median. Outliers have been removed from the plot for clarity.
**Supplementary Figure 7**. Histograms of the depth distribution of the suitable habitat for four focal taxa under present and future climate scenarios. *Acanthogorgia* spp. is shown in (a–f), *Bathypathes thermophila* is shown in (g–l), *Madracis interjecta* is shown in (m–r), and *Stylaster tritoni* is shown in (s, x). For each species, the depth distribution of the habitat is shown under present climate conditions (first column), Shared Socioeconomic Pathway (SSP) 1–1.9 (second column), SSP1‐2.6 (third column), SSP2 4.5 (fourth column), SSP3‐7.0 (fifth column) and SSP5‐8.5 (sixth column) for the year 2090–2100. The average depth is labelled on each plot.
**Supplementary Figure 8**. Histograms of the temperature ranges of the suitable habitat for four focal taxa under present and future climate scenarios. *Acanthogorgia* spp. is shown in (a–f), *Bathypathes thermophila* is shown in (g–l), *Madracis interjecta* is shown in (m–r), and *Stylaster tritoni* is shown in (s, x). For each species, the depth distribution of the habitat is shown under present climate conditions (first column), Shared Socioeconomic Pathway (SSP) 1–1.9 (second column), SSP1‐2.6 (third column), SSP2 4.5 (fourth column), SSP3‐7.0 (fifth column) and SSP5‐8.5 (sixth column) for the year 2090–2100. The average temperature is labelled on each plot.
**Supplementary Figure 9**. Correlations and multicollinearilty between variables in the Red Sea. (a) A correlation matrix between all considered variables across the Red Sea. Darker colours indicate stronger correlations, with positive correlations in red and negative correlations in blue. (b, c) Show the Variance Inflation Factor (VIF) results for variables ultimately used as input for the models, with a common threshold of 5 indicated by a red dashed line. Mean temperature (Temp.) is included in (b), as in the *Bathypathes thermophila* model, while minimum (Min.) and maximum (Max.) temperature are included in (c). Vel. refers to velocity, and VRM is Vector Ruggedness Measure.
**Supplementary Figure 10**. Average latitudes for each focal taxa (*Acanthogorgia* spp. in red, *Bathypathes thermophila* in green, *Madracis interjecta* in blue and *Stylaster tritoni* in purple). Latitude is shown under present climate conditions and five shared socioeconomic pathways (SSPs) for the years 2090–2100 (SSP1‐1.9, SSP1‐2.6, SSP2‐4.5, SSP3‐7.0 and SSP5‐8.5).
**Supplementary Figure 11**. The number of taxa for which the areas in and around NEOM in the Northern Red Sea and Gulf of Aqaba are estimated to be suitable for, where yellow indicates that the habitat is unsuitable for all four study taxa (*Acanthogorgia* spp., *Bathypathes thermophila*, *Madracis interjecta* and *Stylaster tritoni*), and dark blue indicating habitat that is suitable for all four taxa. Results under current climate conditions are shown in (a), and (b–f) show results for the years 2090–2100 under different Shared Socioeconomic Pathways (SSPs); (b) SSP1‐1.9, (c) SSP1‐2.6, (d) SSP2‐4.5, (e) SSP3‐7.0 and (f) SSP5‐8.5. The NEOM boundary is shown as a red line.
**Supplementary Figure 12**. Standard deviation between the 10 runs of the Boosted Regression Tree models. The scale goes from blue (lower standard deviation) to purple (higher standard deviation) for areas in the northern Red Sea (a–d), the central Red Sea (e–h) and the southern Red Sea (i–l). *Acanthogorgia* spp. is shown in (a, e, and i); *Bathypathes thermophila* is shown in (b, f, and j); *Madracis interjecta* is shown in c), f), and j); and *Stylaster tritoni* is shown in (d, h, and l).
**Supplementary Figure 13**. Standard deviation between the 10 runs of the MaxEnt models. The scale goes from blue (lower standard deviation) to purple (higher standard deviation) for areas in the northern Red Sea (a–d), the central Red Sea (e–h) and the southern Red Sea (i–l). *Acanthogorgia* spp. is shown in (a, e, and i); *Bathypathes thermophila* is shown in (b, f, and j); *Madracis interjecta* is shown in (c, f, and j); and *Stylaster tritoni* is shown in (d, h, and l).
**Supplementary Figure 14**. Response curves for *Acanthogorgia* spp. for both Boosted Regression Tree (BRT) and Maxent models. Plots are shown for all variables appearing in Figure 1.
**Supplementary Figure 15**. Response curves for *Bathypathes thermophila* for both Boosted Regression Tree (BRT) and Maxent models. Plots are shown for all variables appearing in Figure 1.
**Supplementary Figure 16**. Response curves for *Madracis interjecta* for both Boosted Regression Tree (BRT) and Maxent models. Plots are shown for all variables appearing in Figure 1.
**Supplementary Figure 17**. Response curves for *Stylaster tritoni* for both Boosted Regression Tree (BRT) and Maxent models. Plots are shown for all variables appearing in Figure 1.
**Supplementary Figure 18**. Models for all scenarios for *Acanthogorgia* spp. generated with Boosted Regression Trees.
**Supplementary Figure 19**. Models for all scenarios for *Bathypathes thermophila* generated with Boosted Regression Trees.
**Supplementary Figure 20**. Models for all scenarios for *Madracis interjecta* generated with Boosted Regression Trees.
**Supplementary Figure 21**. Models for all scenarios for *Stylaster tritoni* generated with Boosted Regression Trees.
**Supplementary Figure 22**. Models for all scenarios for *Acanthogorgia* spp. generated with Maxent.
**Supplementary Figure 23**. Models for all scenarios for *Bathypathes thermophila* generated with Maxent.
**Supplementary Figure 24**. Models for all scenarios for *Madracis interjecta* generated with Maxent.
**Supplementary Figure 25**. Models for all scenarios for *Stylaster tritoni* generated with Maxent.
**Supplementary Figure 26**. Model agreement between Boosted Regression Tree and Maxent models for *Acanthogorgia* spp. The proportion of area assigned to each category is provided in brackets.
**Supplementary Figure 27**. Model agreement between Boosted Regression Tree and Maxent models for *Bathypathes thermophila*. The proportion of area assigned to each category is provided in brackets.
**Supplementary Figure 28**. Model agreement between Boosted Regression Tree and Maxent models for *Madracis interjecta* . The proportion of area assigned to each category is provided in brackets.
**Supplementary Figure 29**. Model agreement between Boosted Regression Tree and Maxent models for *Stylaster tritoni*. The proportion of area assigned to each category is provided in brackets.
**Supplementary Table 1**. Predictor variables used in the models, explanations of their importance and references. VRM is Vector Ruggedness Measure.
**Supplementary Table 3**. The average interaction depth and shrinkage parameters for each Boosted Regression Tree (BRT) model.

**Supplementary Table 2.** The locations of each observation of the four coral taxa, *Acanthogorgia* spp., *Bathypathes thermophila*, *Madracis interjecta* and *Stylaster tritoni* identified from video imagery in the mesophotic and deep eastern Red Sea. See separate electronic material for table.

## Data Availability

The R code used to generate the models and analyses has been published at https://doi.org/10.5281/zenodo.23343821. The bathymetric dataset is published elsewhere and available at https://doi.org/10.5281/zenodo.19065210, and species occurrences, all bathymetric derivatives and regenerated environmental datasets are available at https://doi.org/10.5281/zenodo.23344026.
