# Supplementary material for: Projected Climate Change Impacts on Deep‐Sea Corals of the Red Sea
Source: Glob Chang Biol. 2026 Nov 8;32(11):e71169. doi: 10.1111/gcb.71169 (PMC13746075; doi:10.1111/gcb.71169)
Supplement: Supplementary file 1 [file GCB-32-e71169-s002.pdf]

Supplementary Information

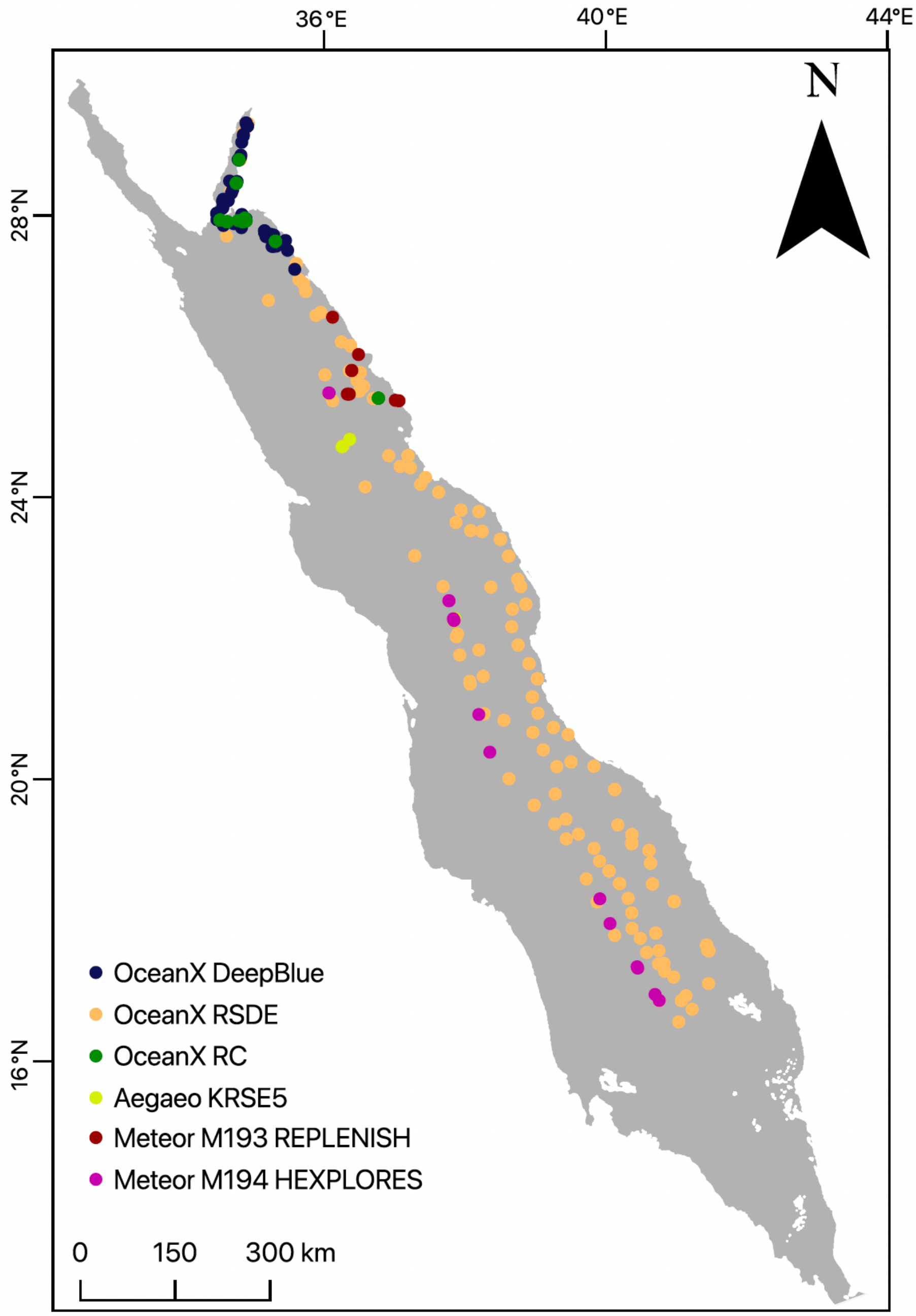

Supplementary Figure 1: The location of Remotely Operated Vehicle (ROV) and submersible dives from all six expeditions that contributed data to this analysis. The expedition names correspond to those in Table 1. RSDE is the Red Sea Decade of Exploration Expedition, RC is the Relationship Cultivation Mission, KRSE is the KAUST Red Sea Expedition.

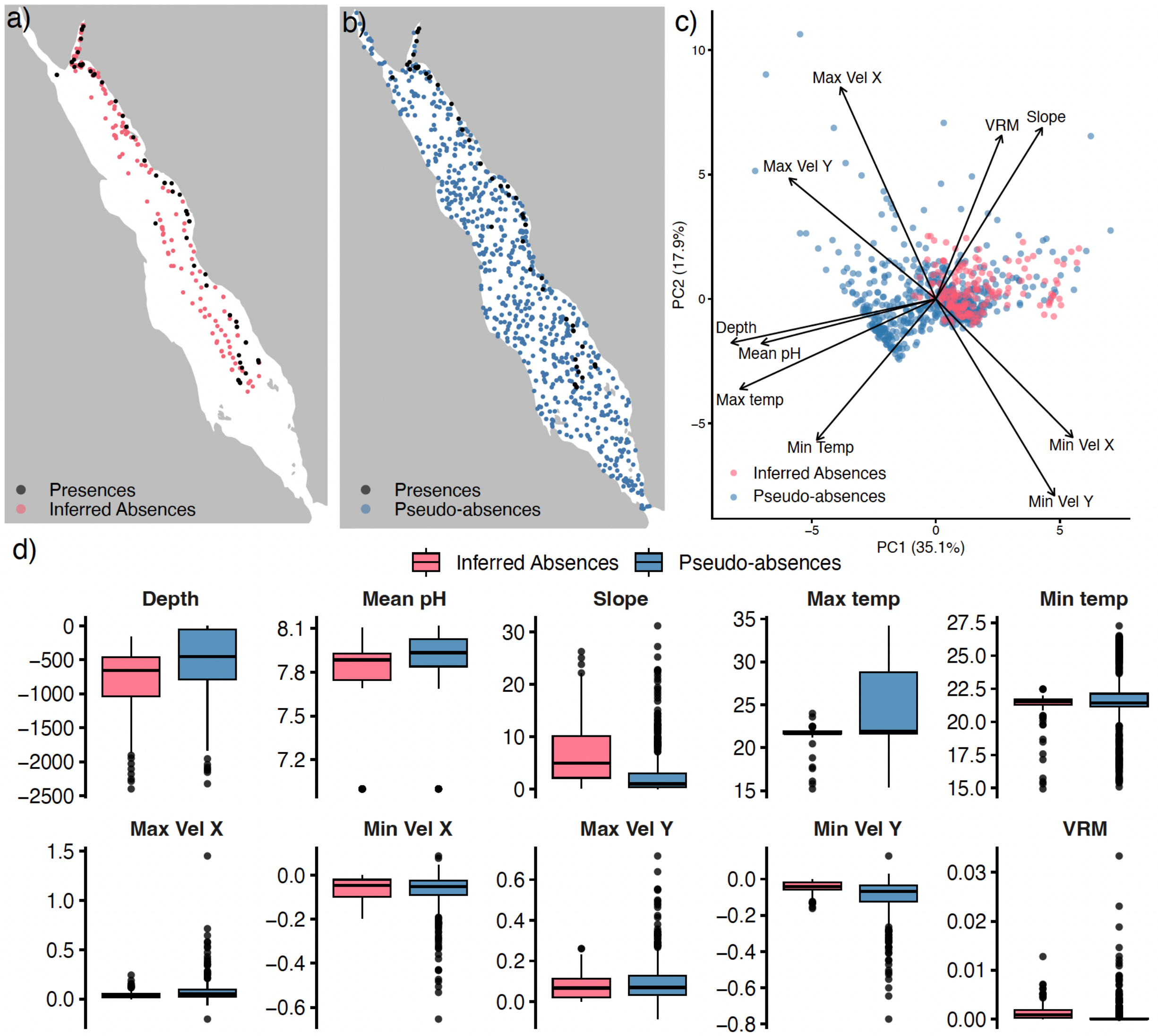

Supplementary Figure 2: Differences in spatial and environmental representation by inferred absences from survey points and generated pseudo-absences based on a trial with *Acanthogorgia* spp.; a) spatial distribution in the Red Sea of inferred absences and presences for *Acanthogorgia* spp.; b) spatial distribution in the Red Sea of pseudoabsences and presences for *Acanthogorgia* spp.; c) a PCA comparing the environmental space which inferred absences (pink) and pseudo-absences (blue) occupy; and d) boxplots showing the representation of the eight environmental variables considered in the *Acanthogorgia* spp. model by inferred absences (pink) and pseudo-absences (blue).

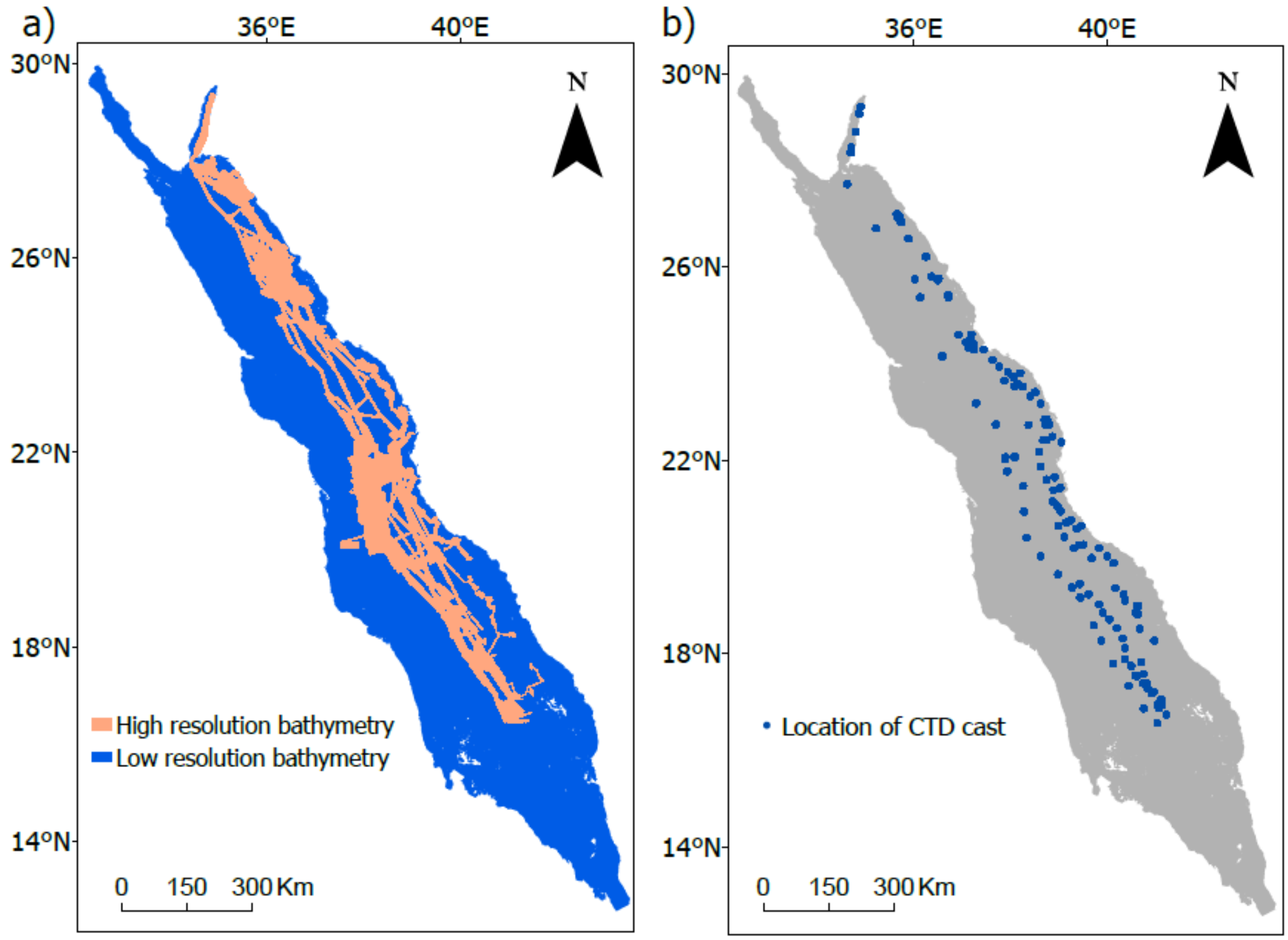

Supplementary Figure 3: The location of acoustic and oceanographic data collection across the Red Sea; a) the areas of bathymetry collected in the Red Sea, with high resolution bathymetry (processed to 30 m) in orange and low resolution bathymetry (at 400 m) in blue; b) the locations of CTD casts are shown in blue.

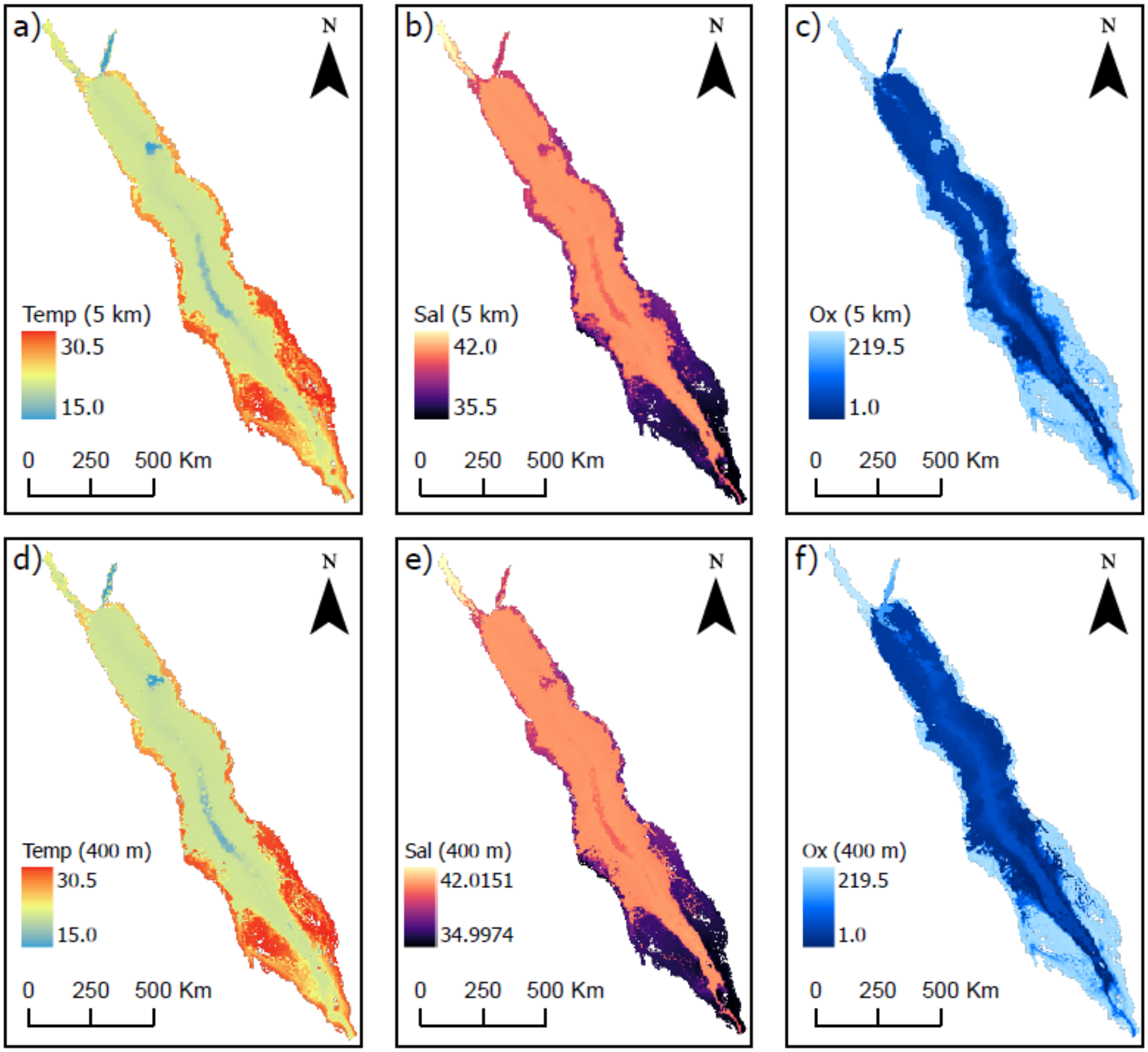

Supplementary Figure 4: The difference between 5 km Bio-Oracle rasters across the Red Sea (a-c) and 400 m reprocessed rasters (d-f) for temperature (a, d), salinity (b, e) and oxygen concentration (c, f).

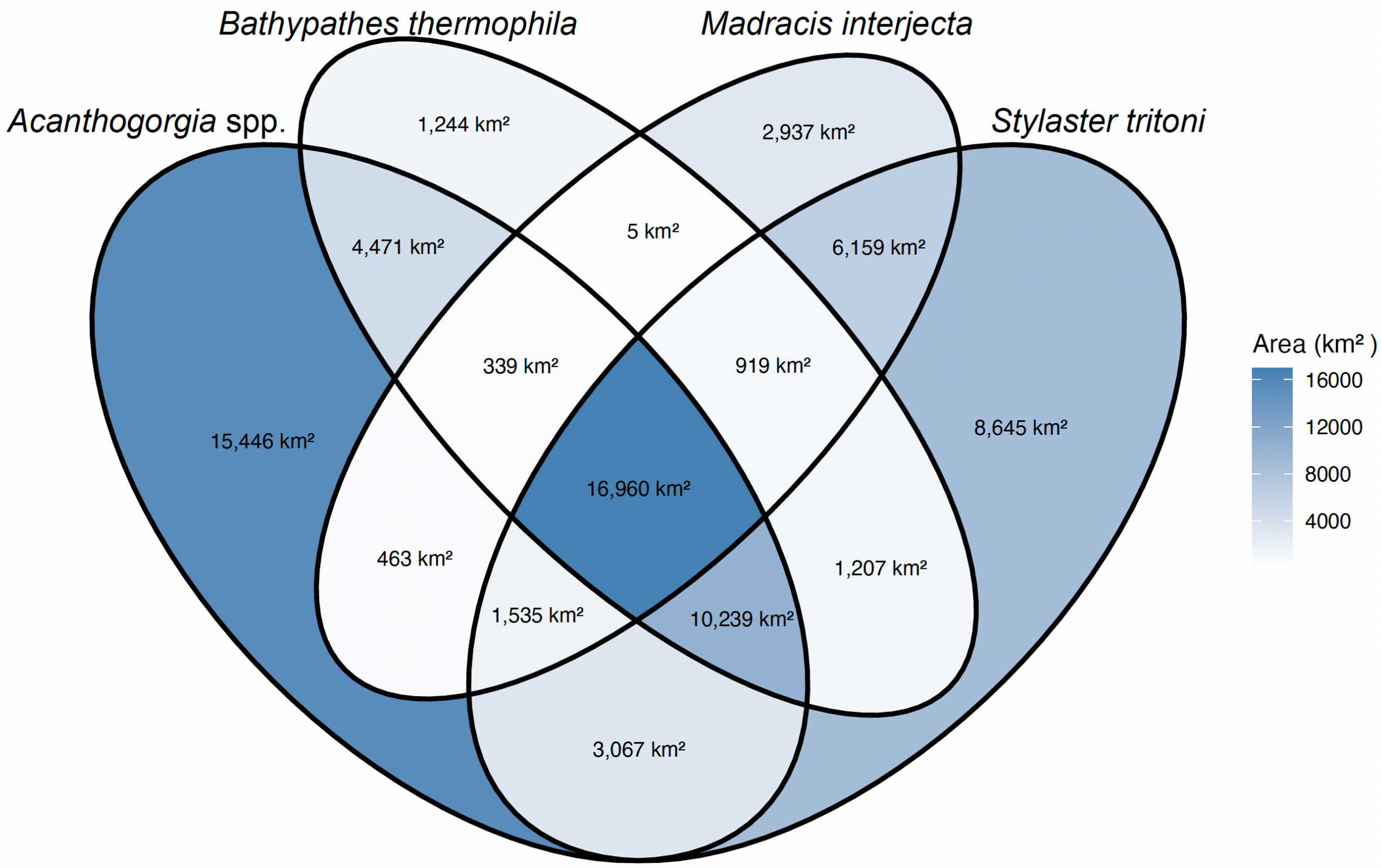

Supplementary Figure 5: The areas of overlap in estimated suitable habitat between the four coral taxa (*Acanthogorgia* spp., *Bathypathes thermophila, Madracis interjecta* and *Stylaster tritoni*) across the Red Sea and under present environmental conditions. Area where habitat is not predicted to be suitable for any species is not shown.

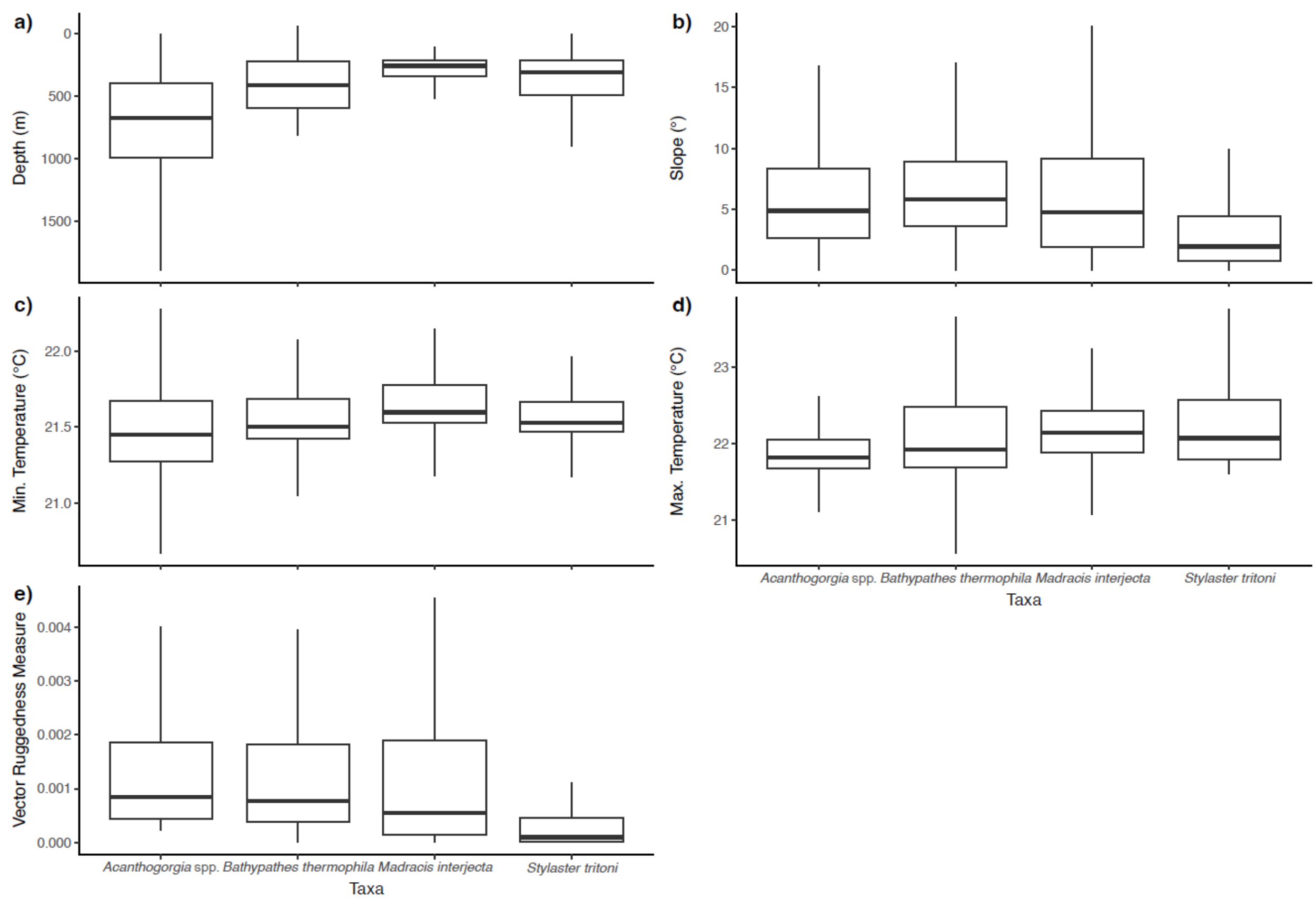

Supplementary Figure 6: The environmental conditions in which four coral taxa (*Acanthogorgia* spp., *Bathypathes thermophila, Madracis interjecta* and *Stylaster tritoni*) were observed in the Red Sea. The five variables shown here (depth, slope, minimum temperature, maximum temperature and vector ruggedness measure) are the most commonly selected and most important in the models. Minimum and maximum temperatures refer to the seawater temperature at the seafloor. The boxplots show the interquartile range as boxes, with whisker lines extending to 1.5 times the interquartile range. The central line is the median. Outliers have been removed from the plot for clarity.

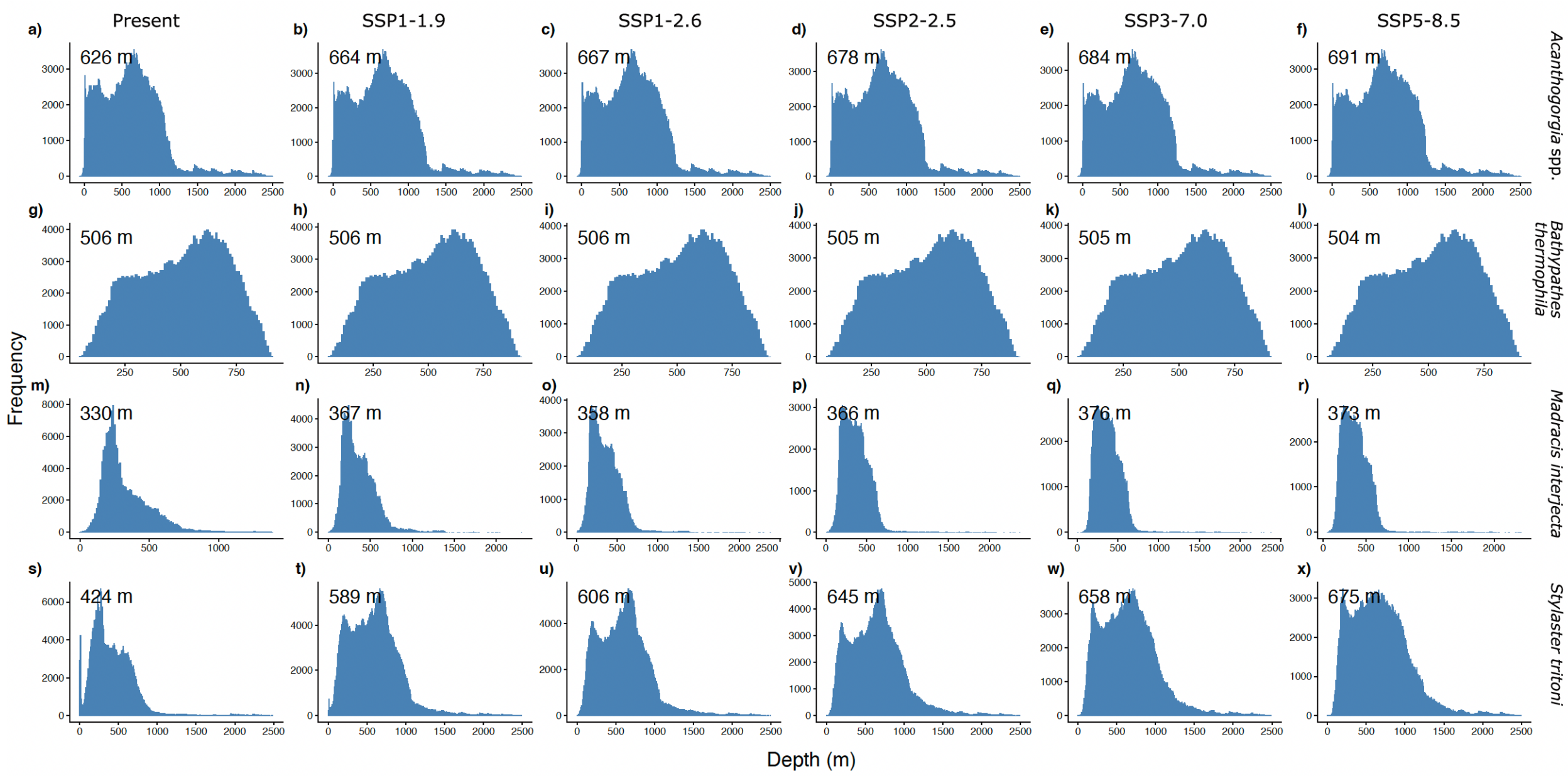

Supplementary Figure 7: Histograms of the depth distribution of the suitable habitat for four focal taxa under present and future climate scenarios. *Acanthogorgia* spp. is shown in a)-f), *Bathypathes thermophila* is shown in g)-l), *Madracis interjecta* is shown in m)-r), and *Stylaster tritoni* is shown in s)-x). For each species, the depth distribution of the habitat is shown under present climate conditions (first column), Shared Socioeconomic Pathway (SSP) 1-1.9 (second column), SSP1-2.6 (third column), SSP2-4.5 (fourth column), SSP3-7.0 (fifth column) and SSP5-8.5 (sixth column) for the year 2090-2100. The average depth is labelled on each plot.

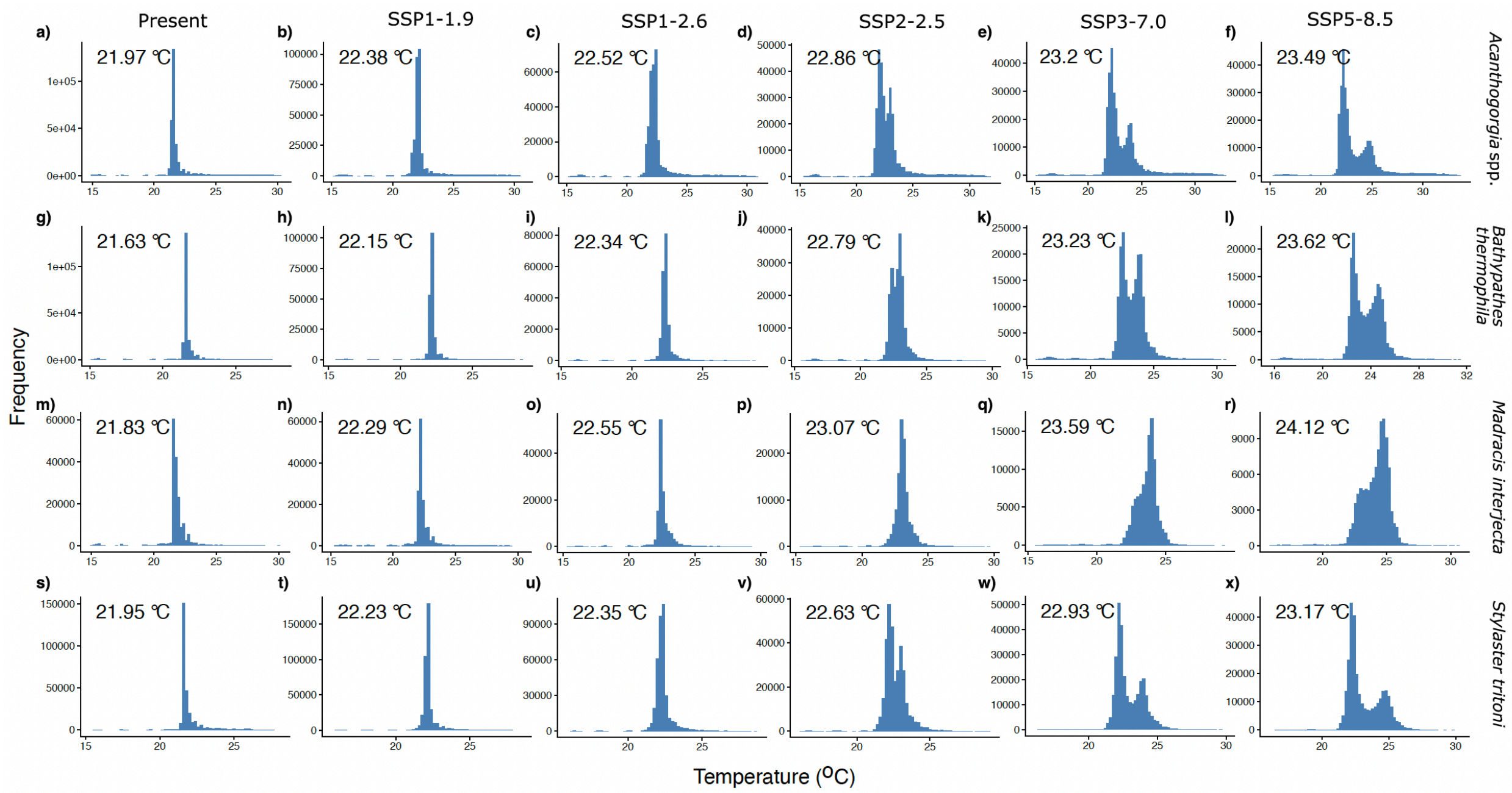

Supplementary Figure 8: Histograms of the temperature ranges of the suitable habitat for four focal taxa under present and future climate scenarios. *Acanthogorgia* spp. is shown in a)-f), *Bathypathes thermophila* is shown in g)-l), *Madracis interjecta* is shown in m)-r), and *Stylaster tritoni* is shown in s)-x). For each species, the depth distribution of the habitat is shown under present climate conditions (first column), Shared Socioeconomic Pathway (SSP) 1-1.9 (second column), SSP1-2.6 (third column), SSP2-4.5 (fourth column), SSP3-7.0 (fifth column) and SSP5-8.5 (sixth column) for the year 2090-2100. The average temperature is labelled on each plot.

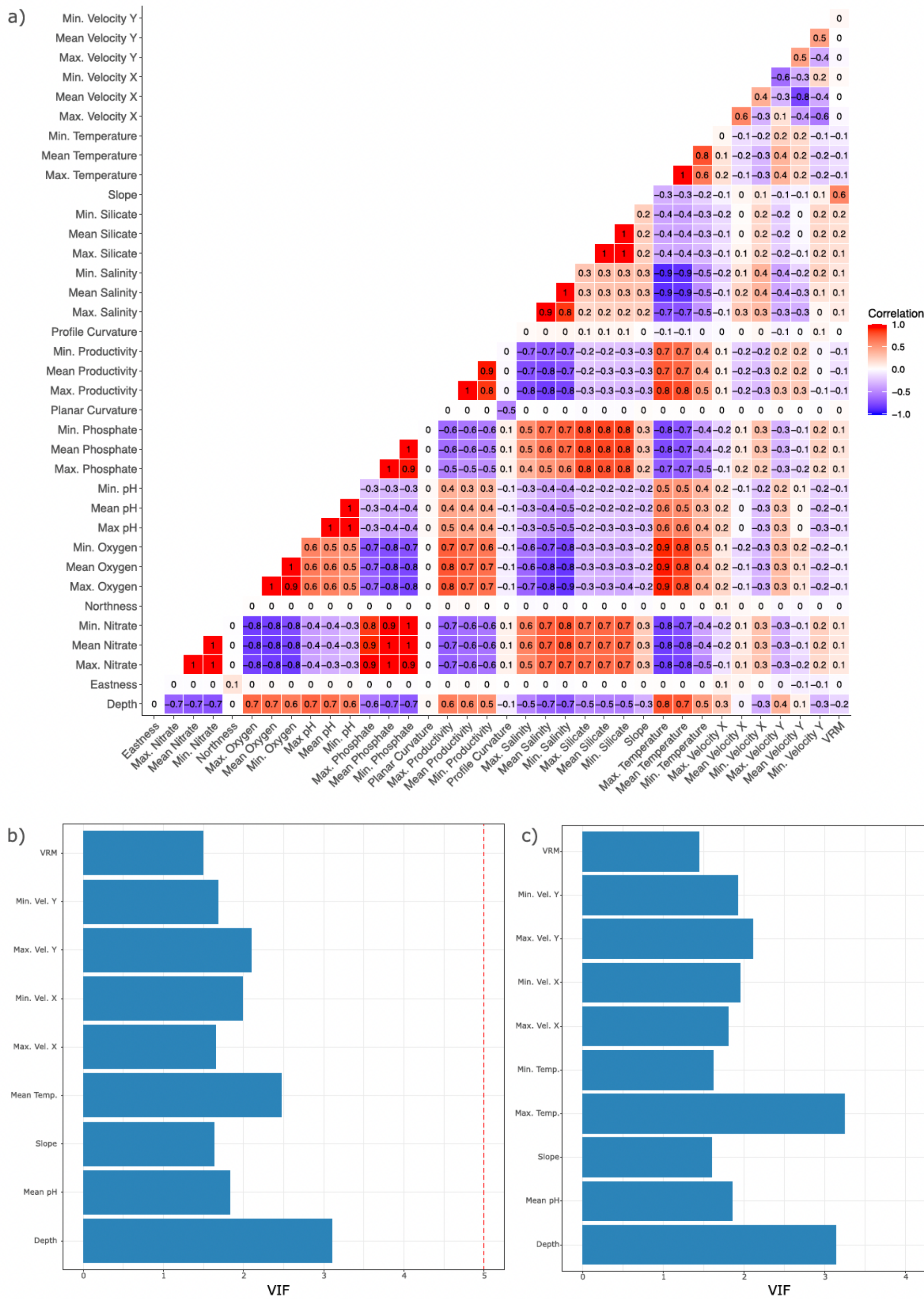

Supplementary Figure 9: Correlations and multicollinearilty between variables in the Red Sea. a) A correlation matrix between all considered variables across the Red Sea. Darker colours indicate stronger correlations, with positive correlations in red and negative correlations in blue. b) and c) show the Variance Inflation Factor (VIF) results for variables ultimately used as input for the models, with a common threshold of 5 indicated by a red dashed line. Mean temperature (Temp.) is included in b), as in the *Bathypathes thermophila* model, while minimum (Min.) and maximum (Max.) temperature are included in c). Vel. refers to velocity, and VRM is Vector Ruggedness Measure.

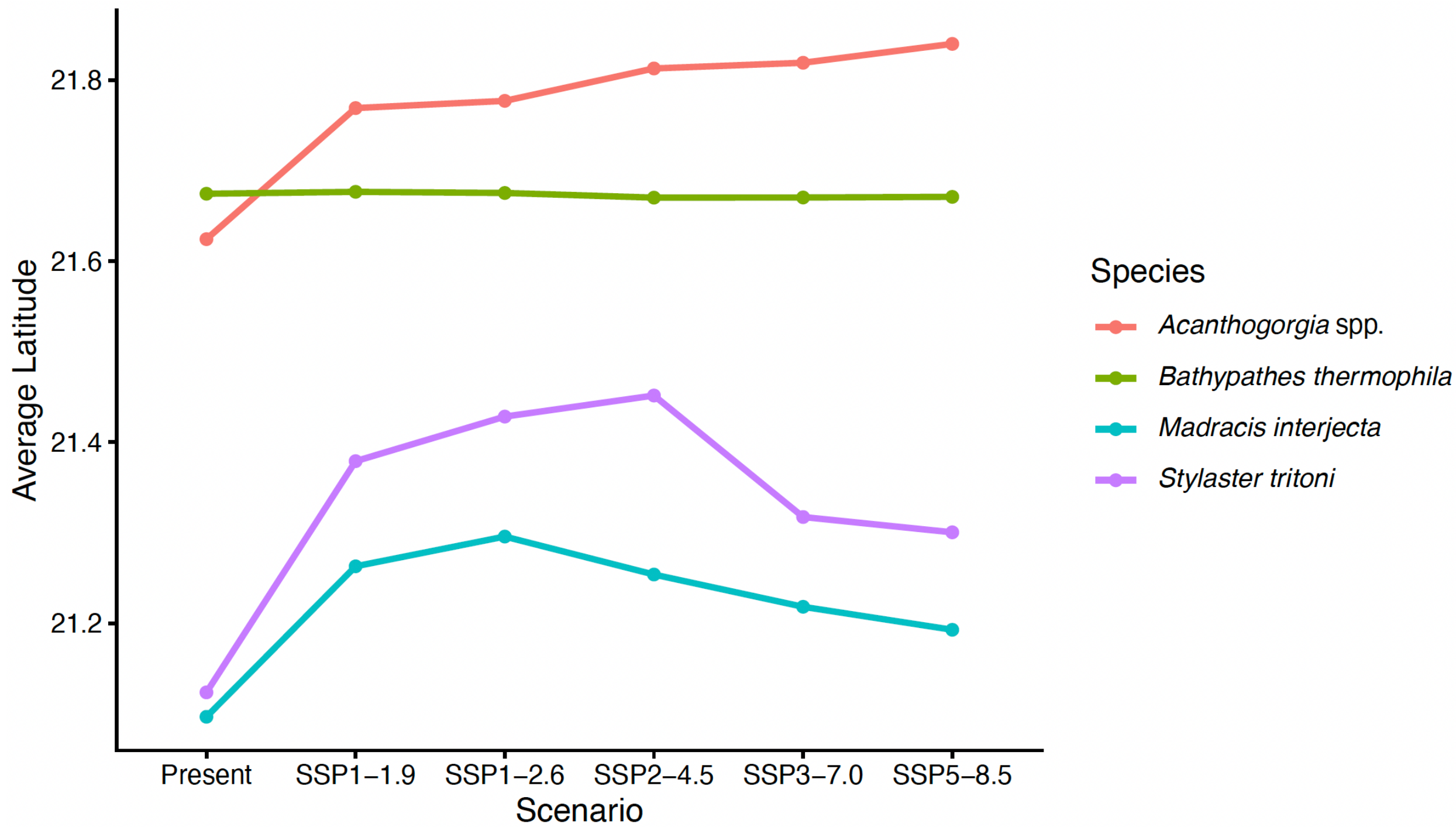

Supplementary Figure 10: Average latitudes for each focal taxa (*Acanthogorgia* spp. in red, *Bathypathes thermophila* in green, *Madracis interjecta* in blue and *Stylaster tritoni* in purple). Latitude is shown under present climate conditions and five shared socioeconomic pathways (SSPs) for the years 2090-2100 (SSP1-1.9, SSP1-2.6, SSP2-4.5, SSP3-7.0 and SSP5-8.5).

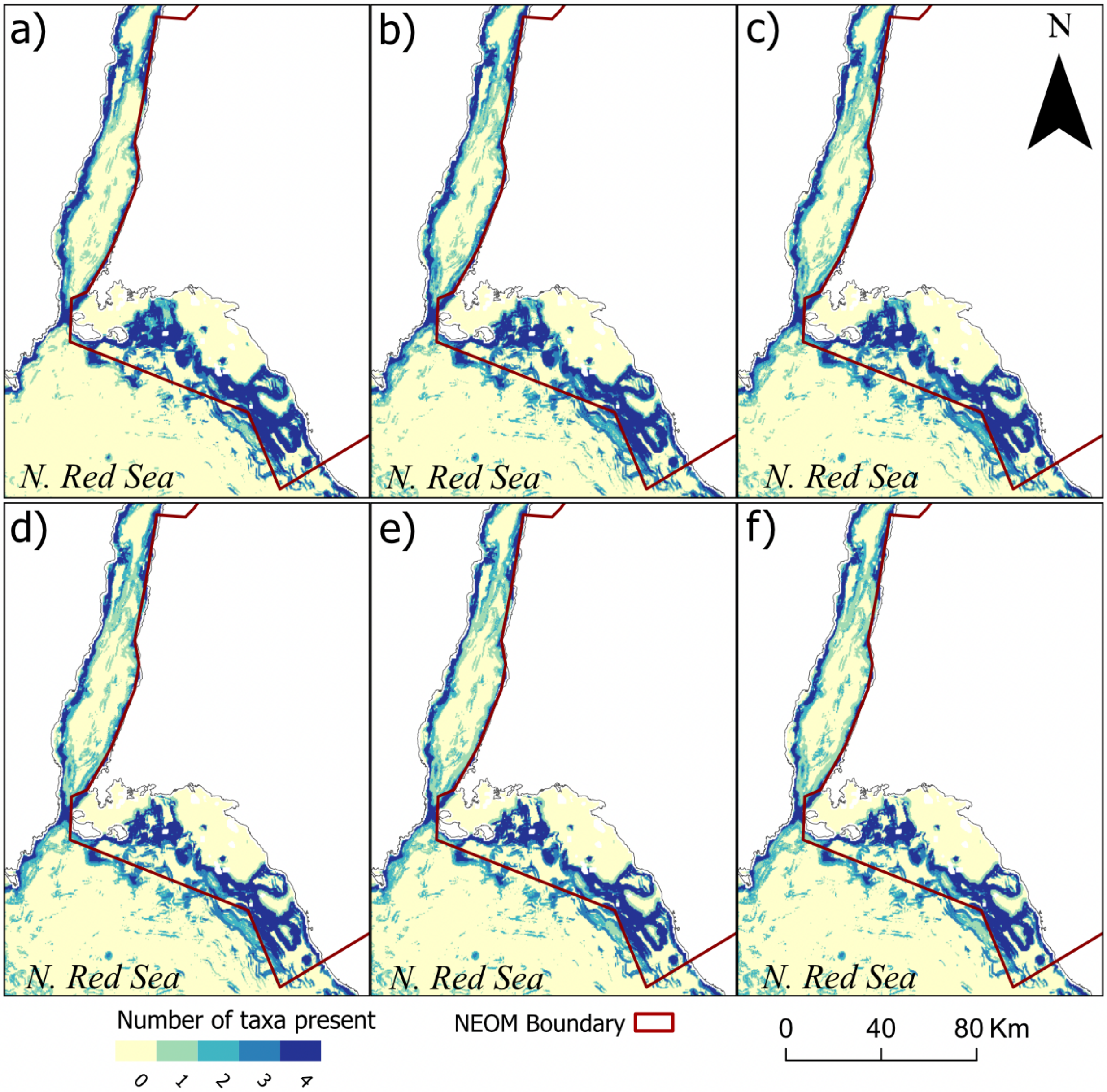

Supplementary Figure 11: The number of taxa for which the areas in and around NEOM in the Northern Red Sea and Gulf of Aqaba are estimated to be suitable for, where yellow indicates that the habitat is unsuitable for all four study taxa (*Acanthogorgia* spp., *Bathypathes thermophila*, *Madracis interjecta* and *Stylaster tritoni*), and dark blue indicating habitat that is suitable for all four taxa. Results under current climate conditions are shown in a), and b) to f) show results for the years 2090-2100 under different Shared Socioeconomic Pathways (SSPs); b) SSP1-1.9, c) SSP1-2.6, d) SSP2-4.5, e) SSP3-7.0 and f) SSP5-8.5. The NEOM boundary is shown as a red line.

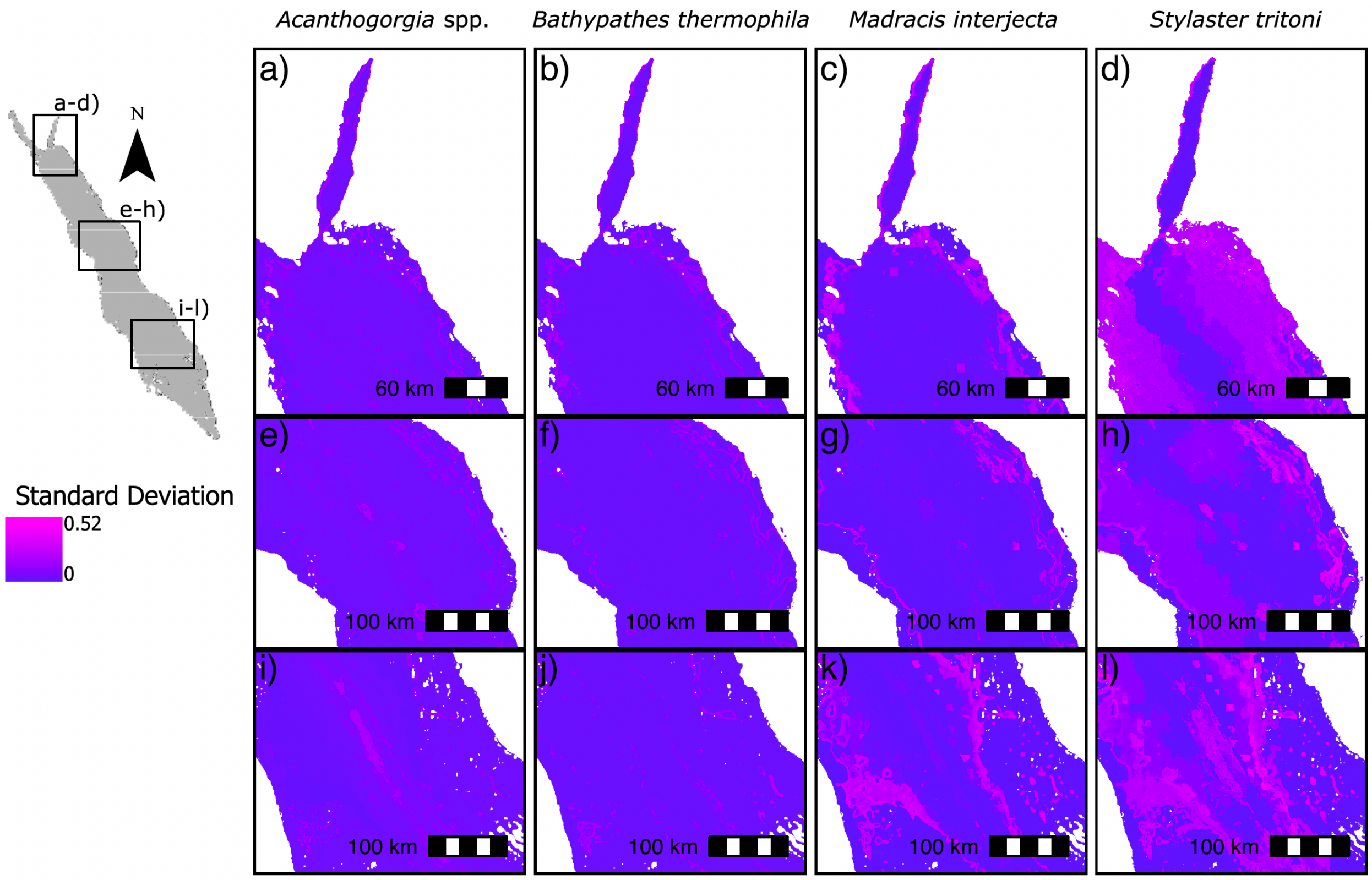

Supplementary Figure 12: Standard deviation between the ten runs of the Boosted Regression Tree models. The scale goes from blue (lower standard deviation) to purple (higher standard deviation) for areas in the northern Red Sea (a-d), the central Red Sea (e-h) and the southern Red Sea (i-l). *Acanthogorgia* spp. is shown in a), e) and i); *Bathypathes thermophila* is shown in b), f) and j); *Madracis interjecta* is shown in c), f), and j); and *Stylaster tritoni* is shown in d), h) and l).

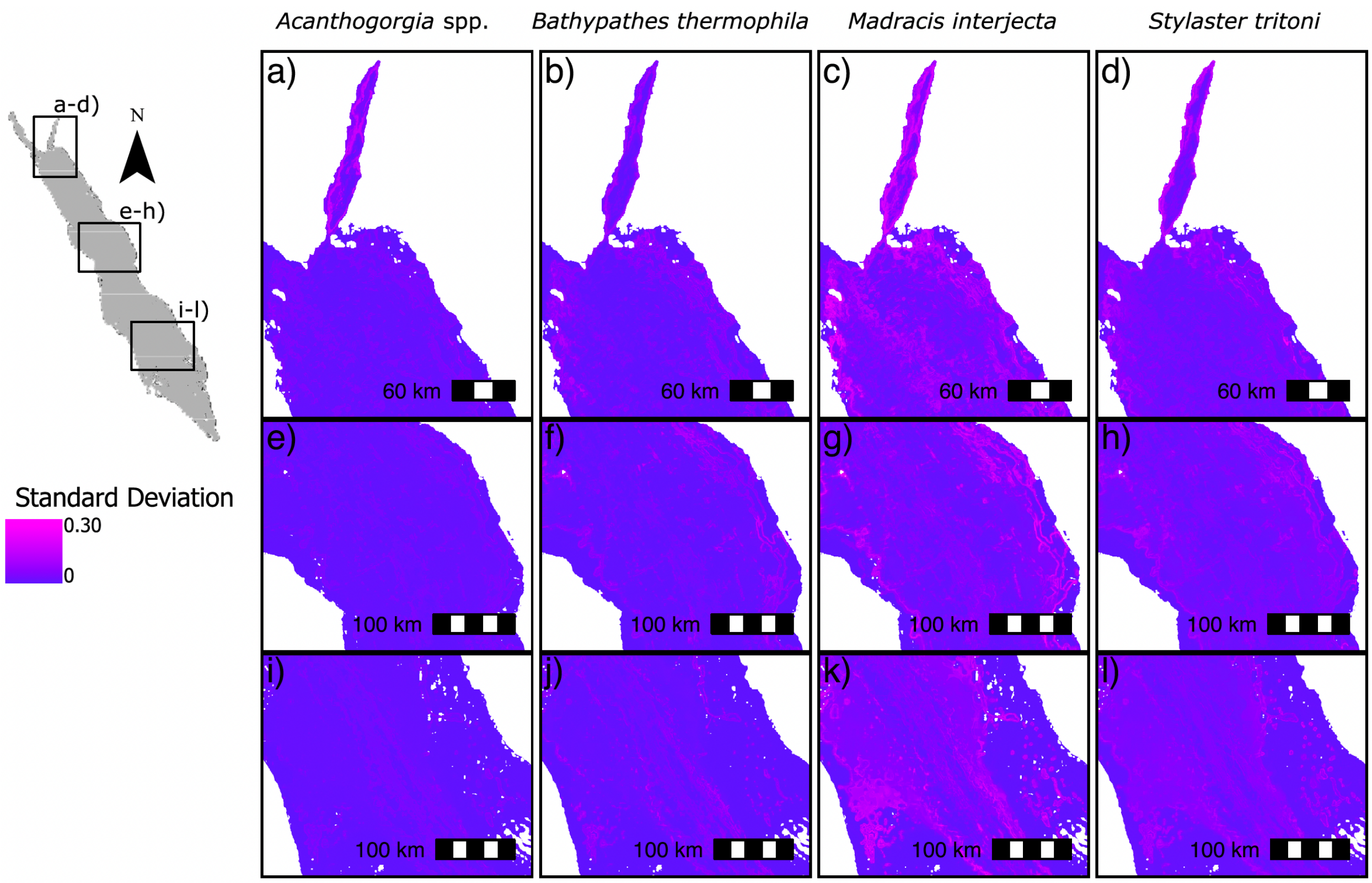

Supplementary Figure 13: Standard deviation between the ten runs of the MaxEnt models. The scale goes from blue (lower standard deviation) to purple (higher standard deviation) for areas in the northern Red Sea (a-d), the central Red Sea (e-h) and the southern Red Sea (i-l). *Acanthogorgia* spp. is shown in a), e) and i); *Bathypathes thermophila* is shown in b), f) and j); *Madracis interjecta* is shown in c), f), and j); and *Stylaster tritoni* is shown in d), h) and l).

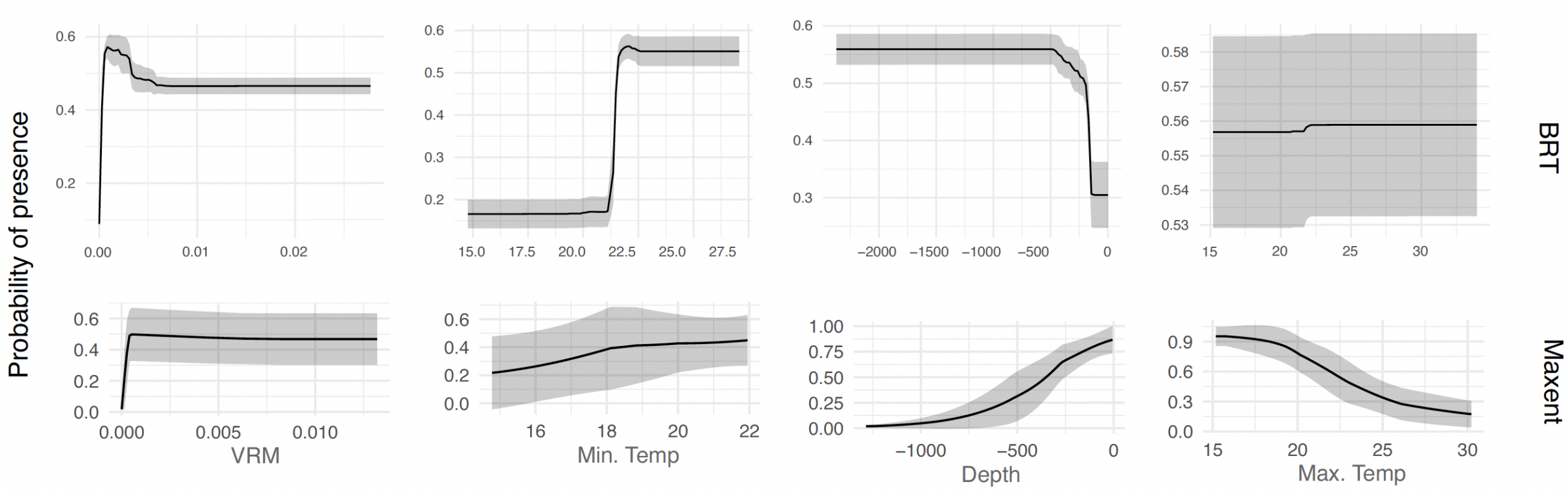

Supplementary Figure 14: Response curves for *Acanthogorgia* spp. for both Boosted Regression Tree (BRT) and Maxent models. Plots are shown for all variables appearing in Figure 1.

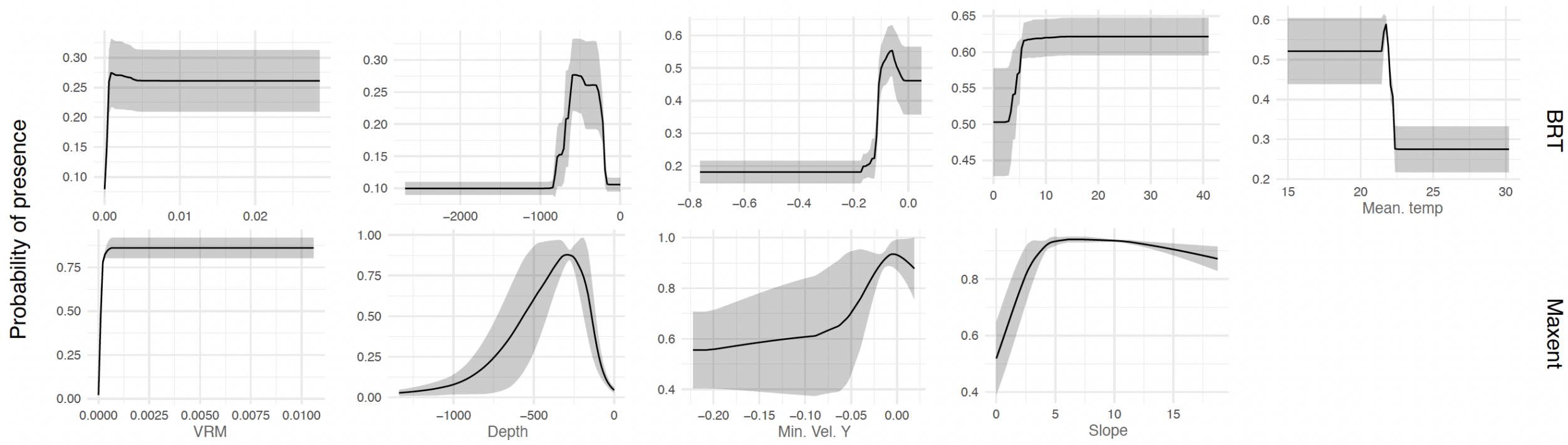

Supplementary Figure 15: Response curves for *Bathypathes thermophila* for both Boosted Regression Tree (BRT) and Maxent models. Plots are shown for all variables appearing in Figure 1.

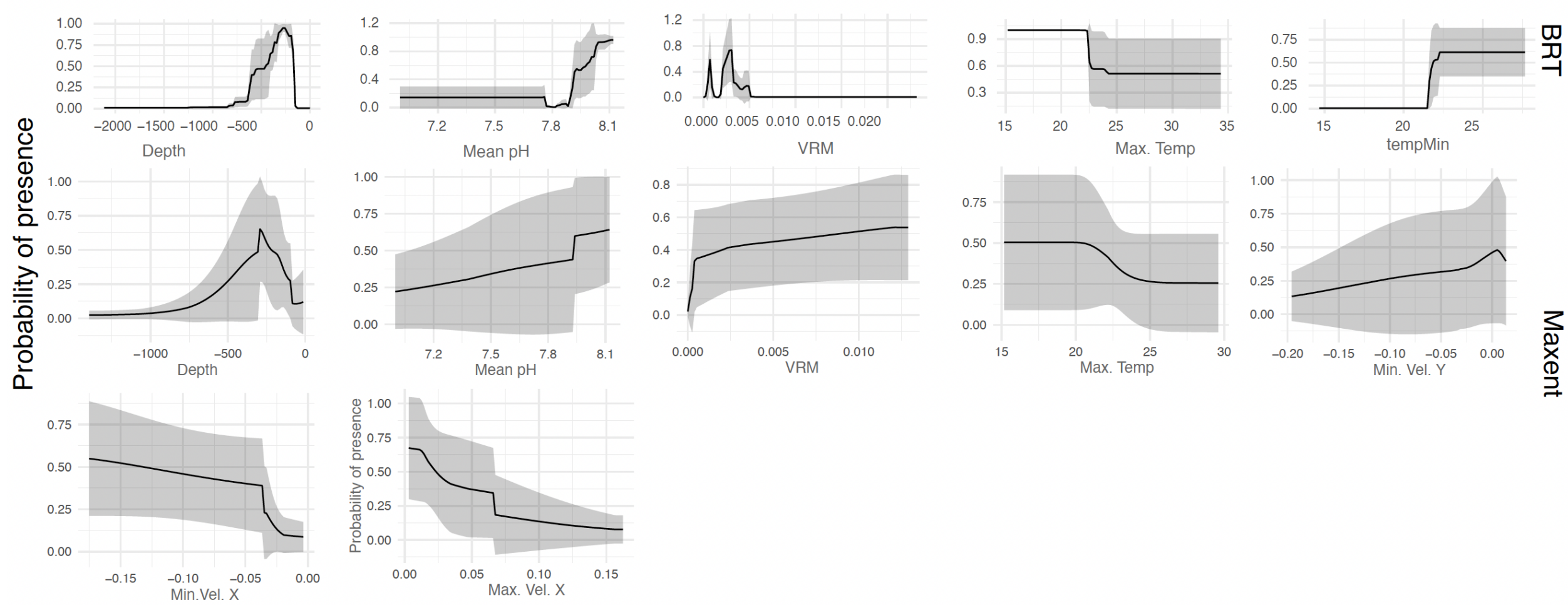

Supplementary Figure 16: Response curves for *Madracis interjecta* for both Boosted Regression Tree (BRT) and Maxent models. Plots are shown for all variables appearing in Figure 1.

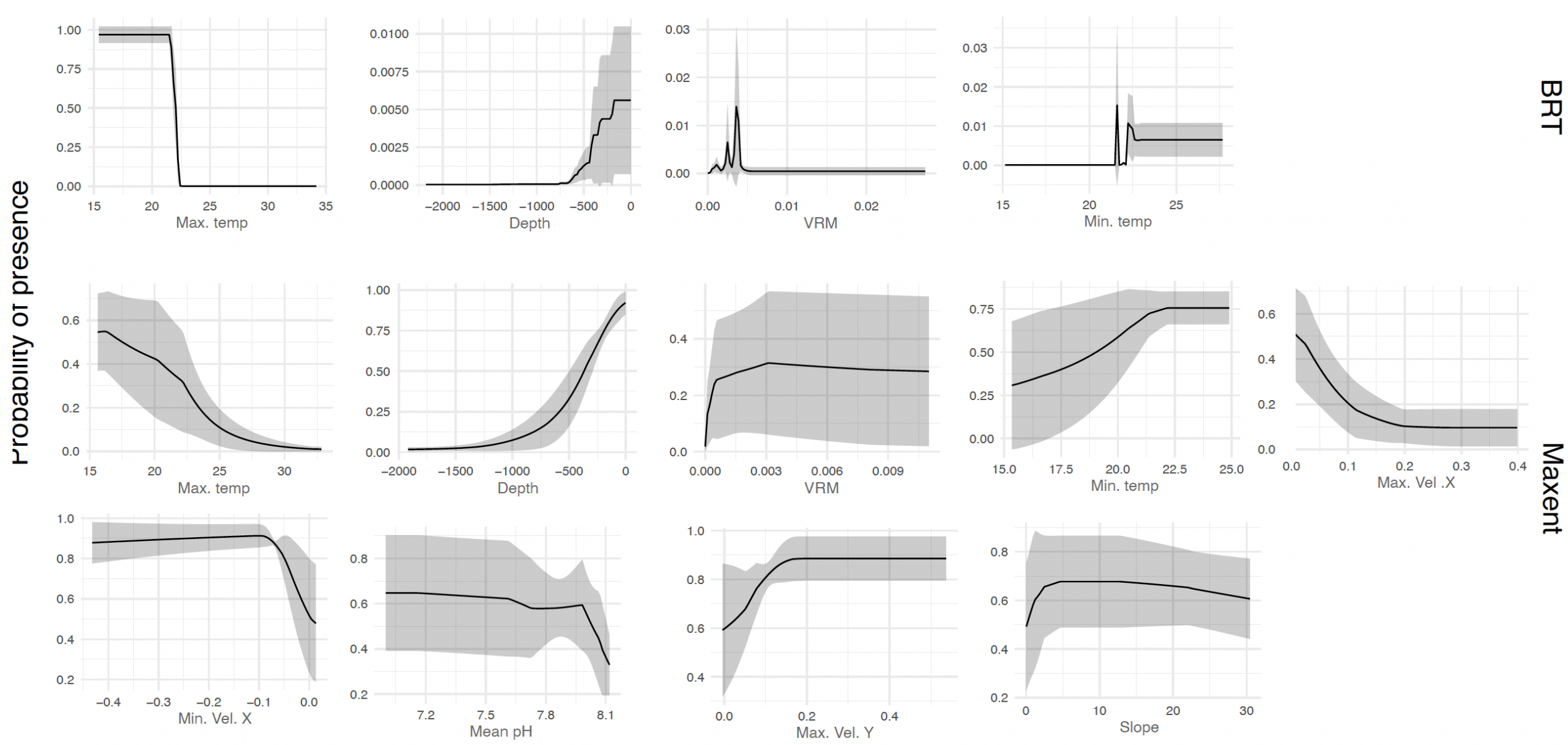

Supplementary Figure 17: Response curves for *Stylaster tritoni* for both Boosted Regression Tree (BRT) and Maxent models. Plots are shown for all variables appearing in Figure 1.

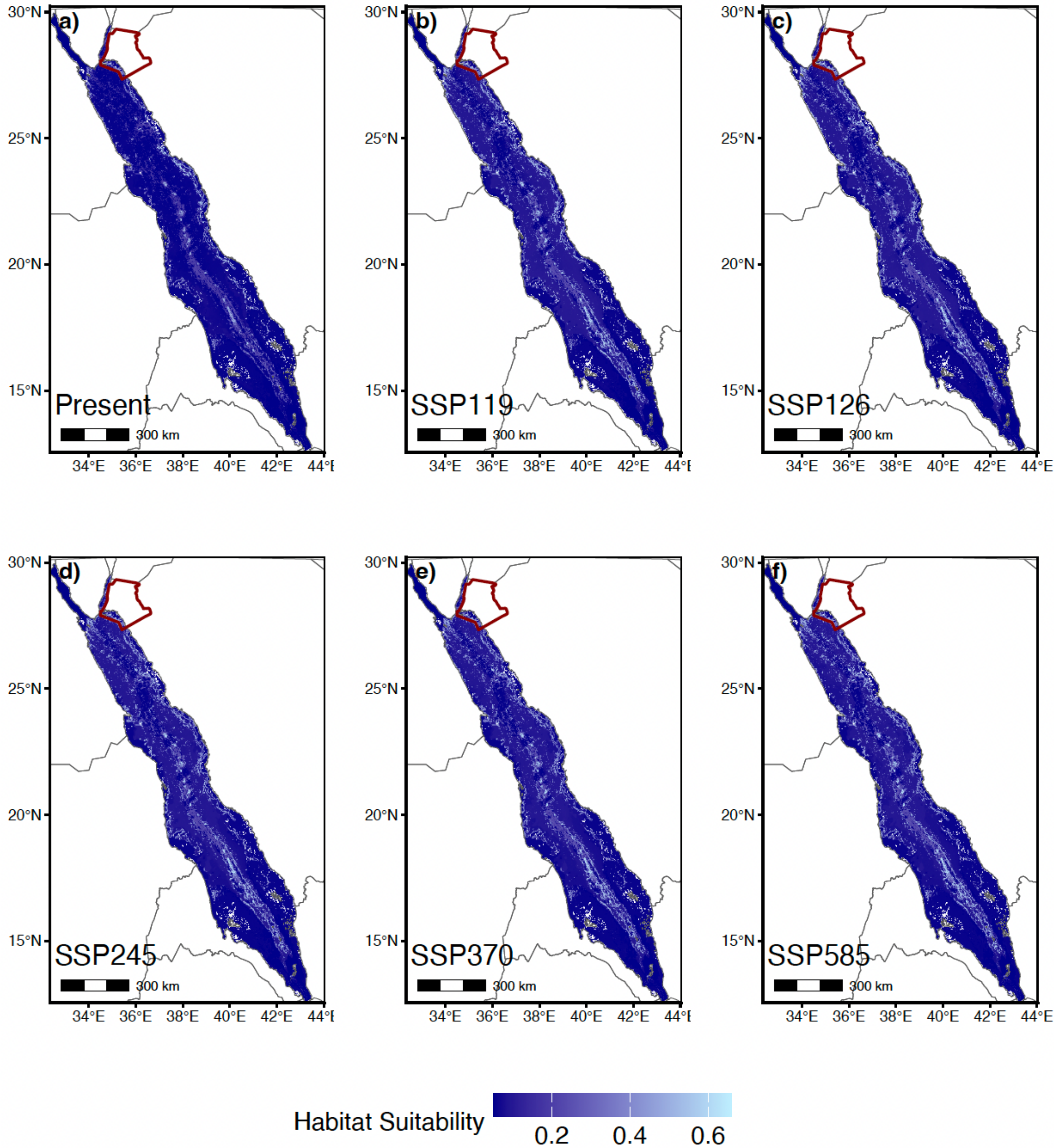

Supplementary Figure 18: Models for all scenarios for *Acanthogorgia* spp. generated with Boosted Regression Trees.

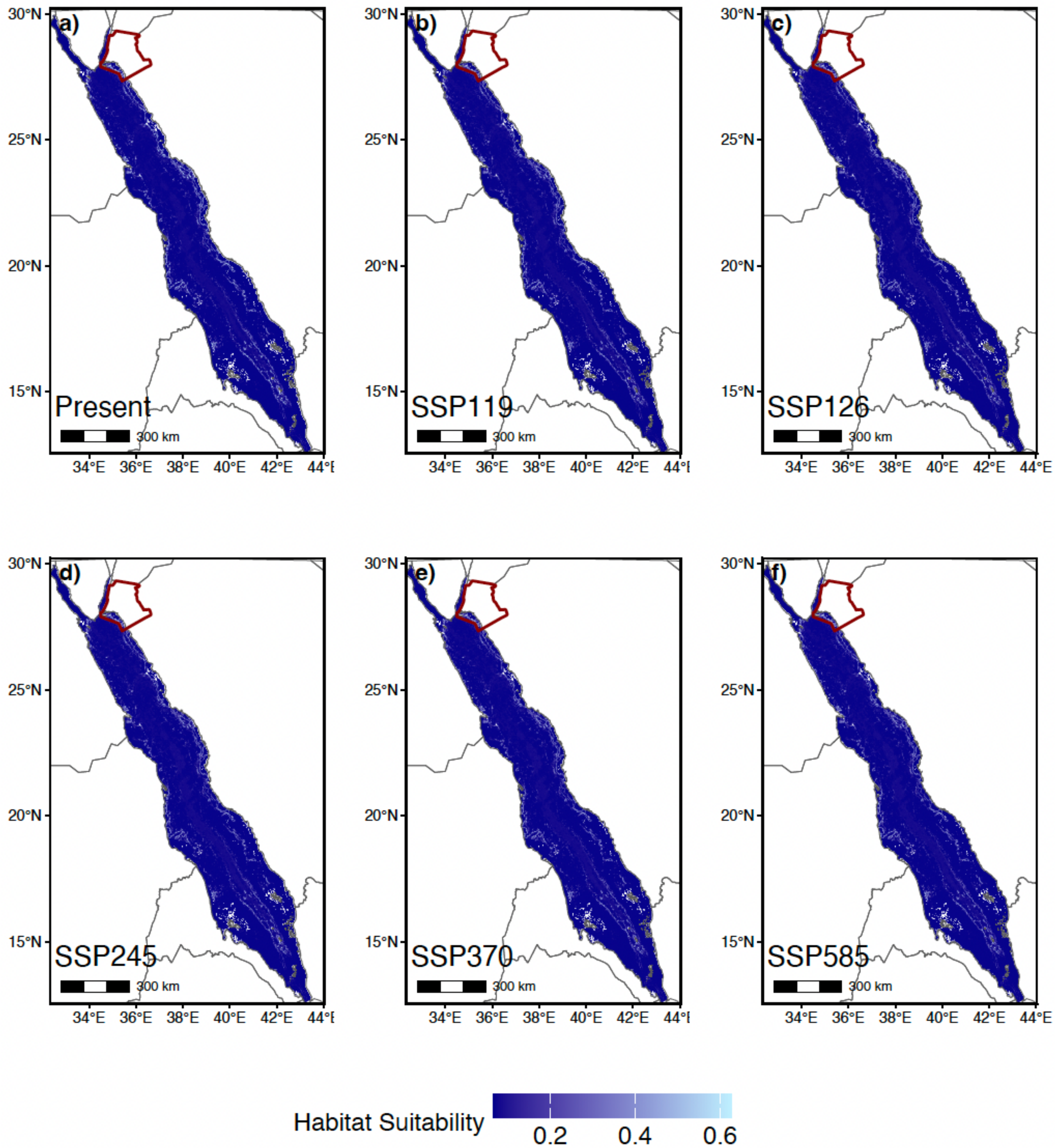

Supplementary Figure 19: Models for all scenarios for *Bathypathes thermophila* generated with Boosted Regression Trees.

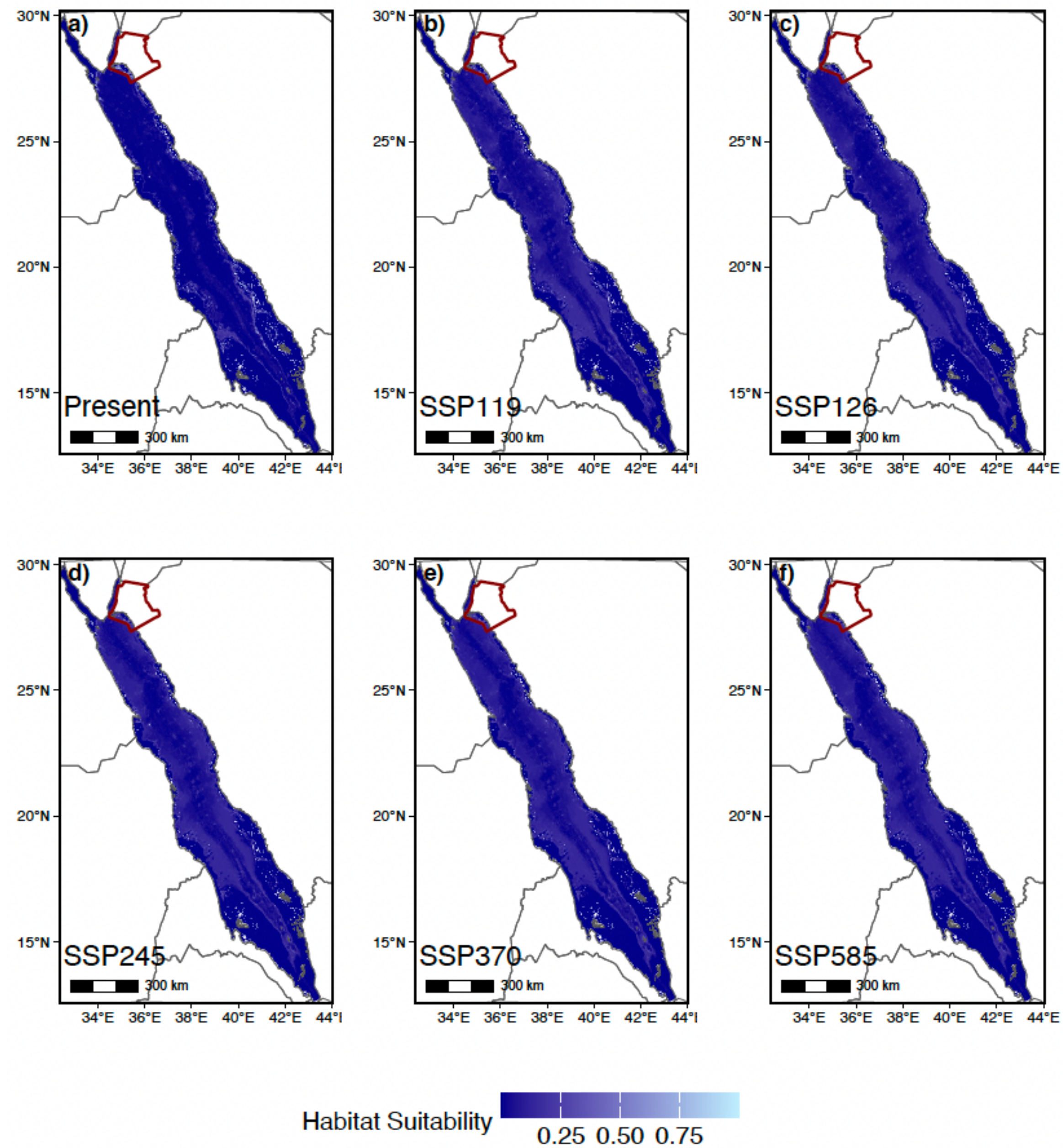

Supplementary Figure 20: Models for all scenarios for *Madracis interjecta* generated with Boosted Regression Trees.

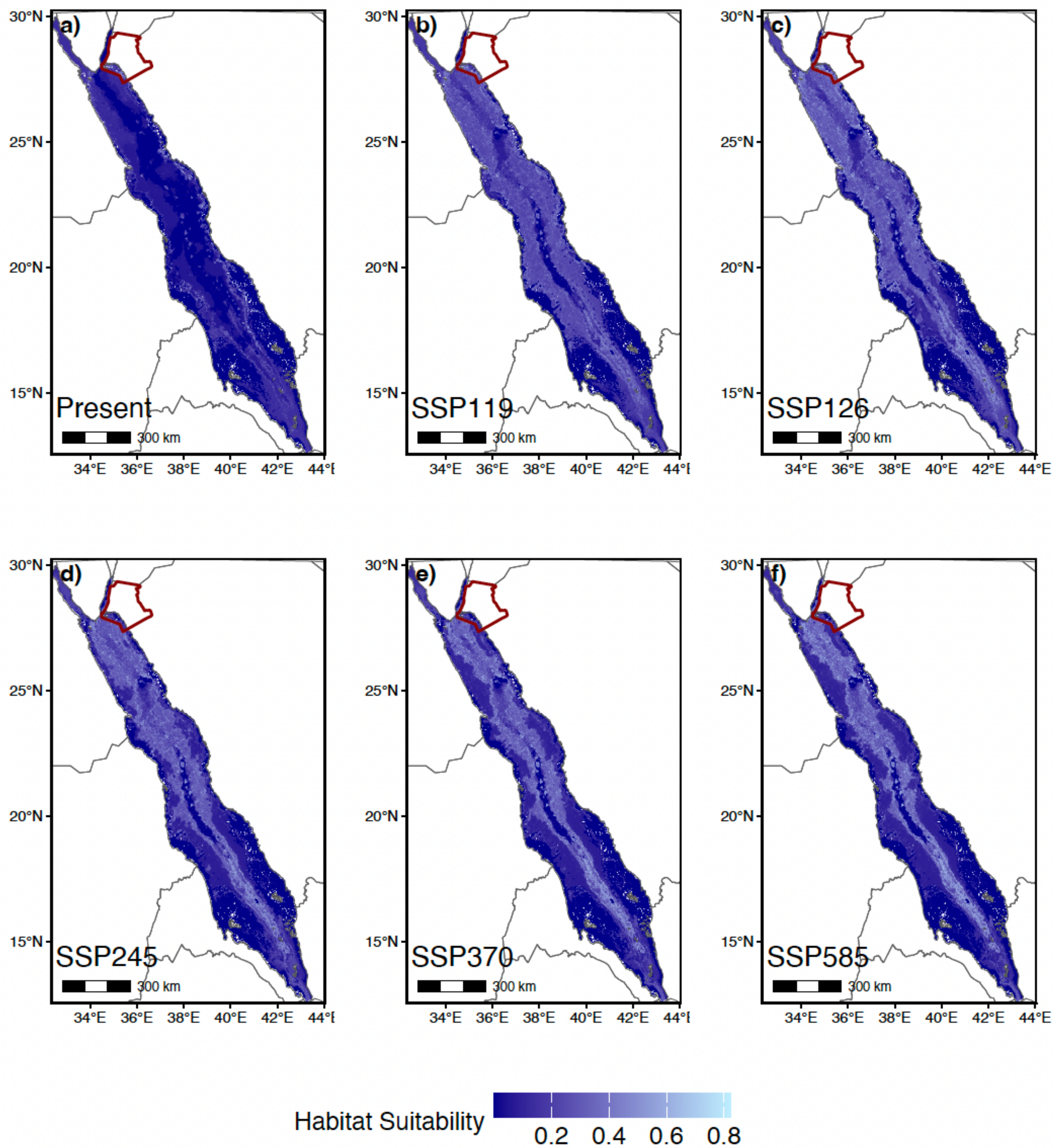

Supplementary Figure 21: Models for all scenarios for *Stylaster tritoni* generated with Boosted Regression Trees.

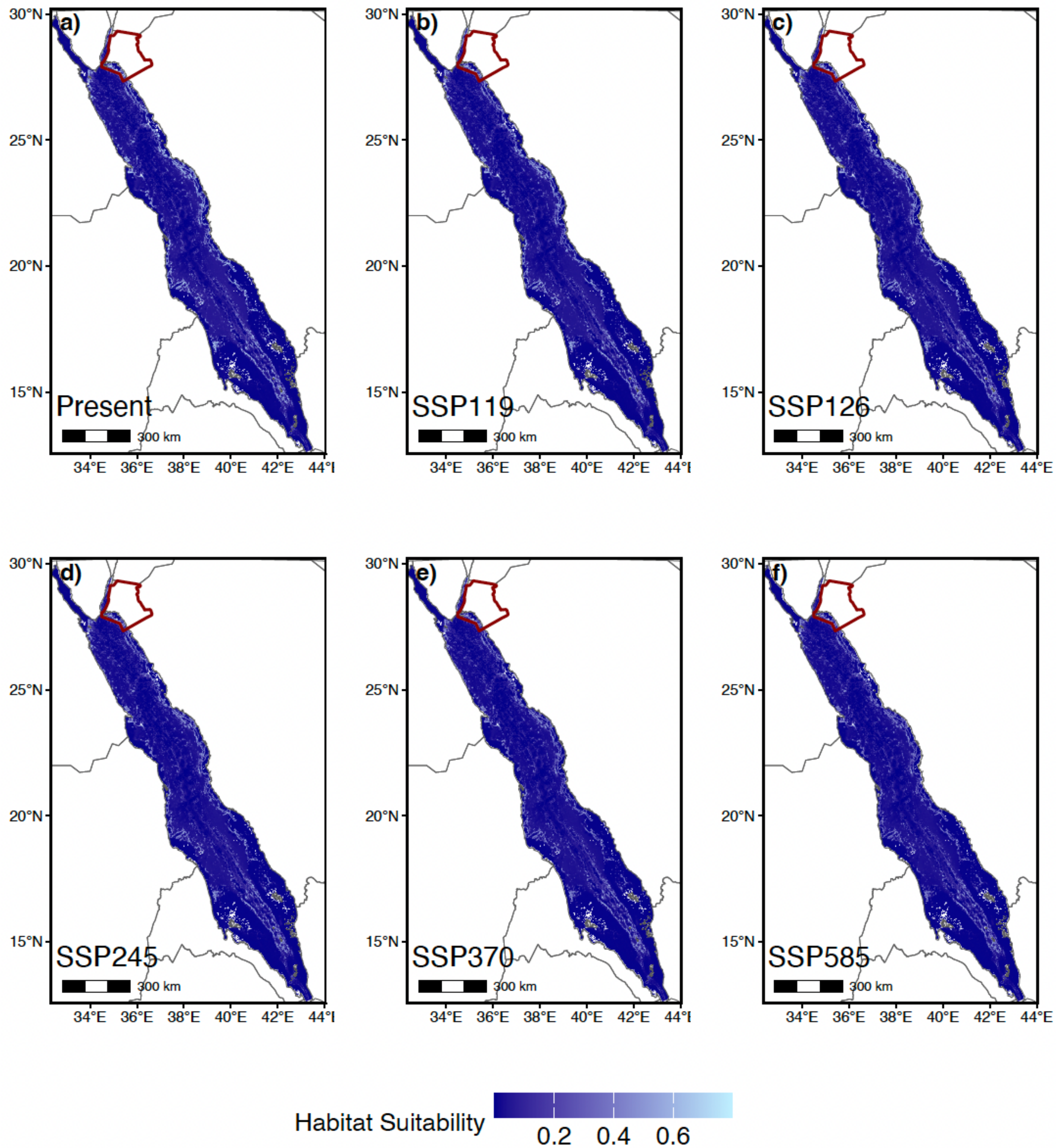

Supplementary Figure 22: Models for all scenarios for *Acanthogorgia* spp. generated with Maxent.

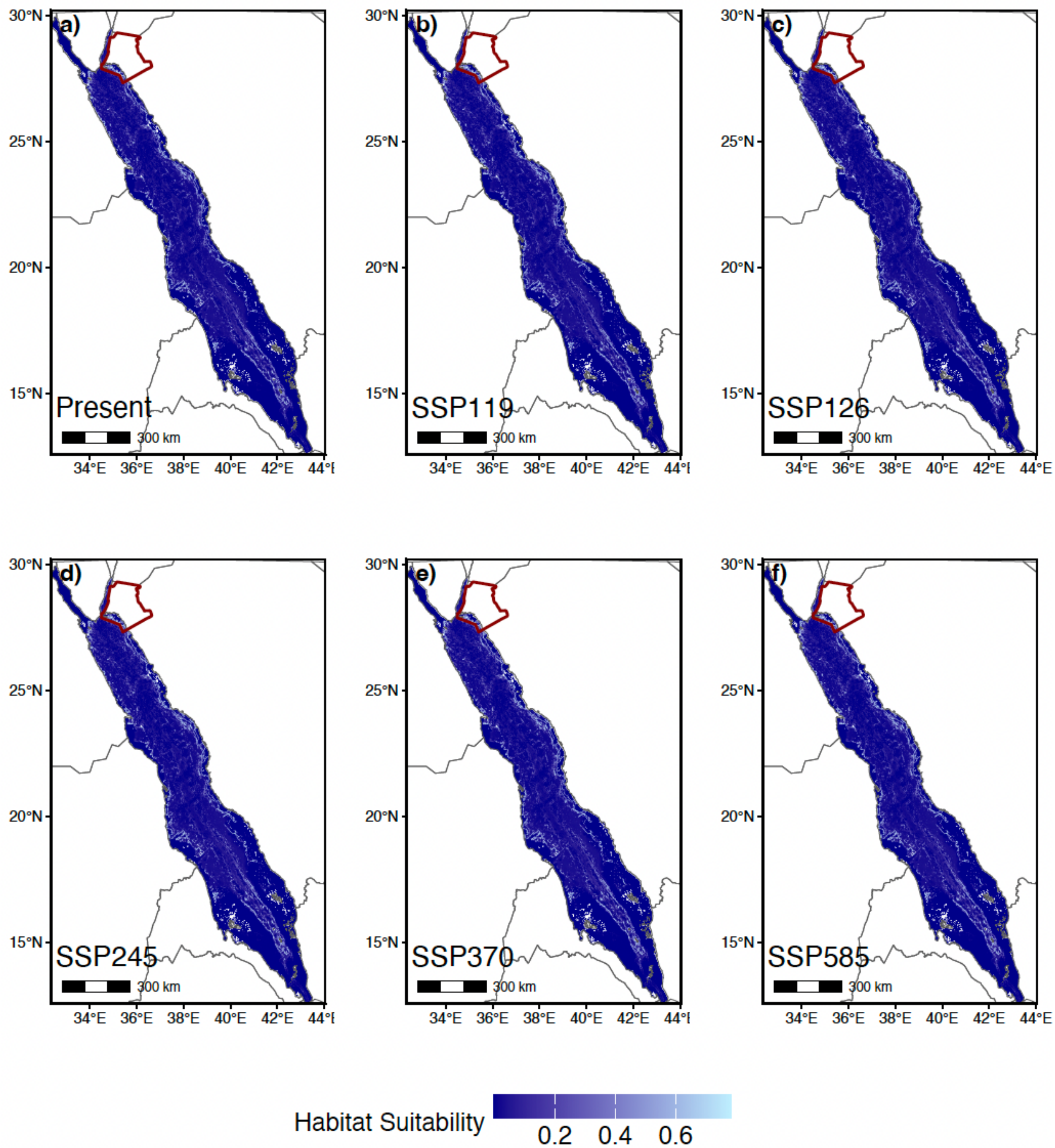

Supplementary Figure 23: Models for all scenarios for *Bathypathes thermophila* generated with Maxent.

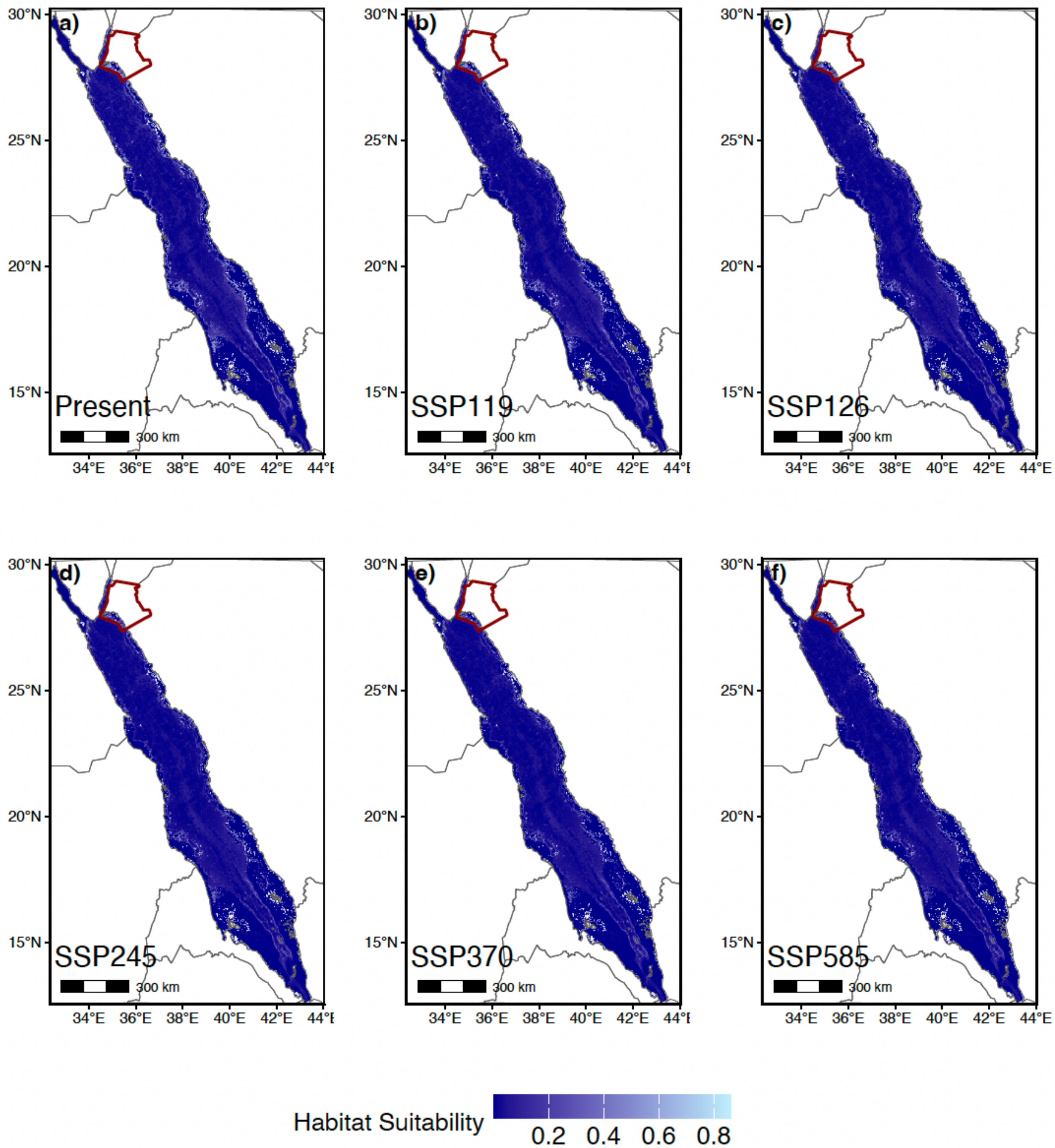

Supplementary Figure 24: Models for all scenarios for *Madracis interjecta* generated with Maxent.

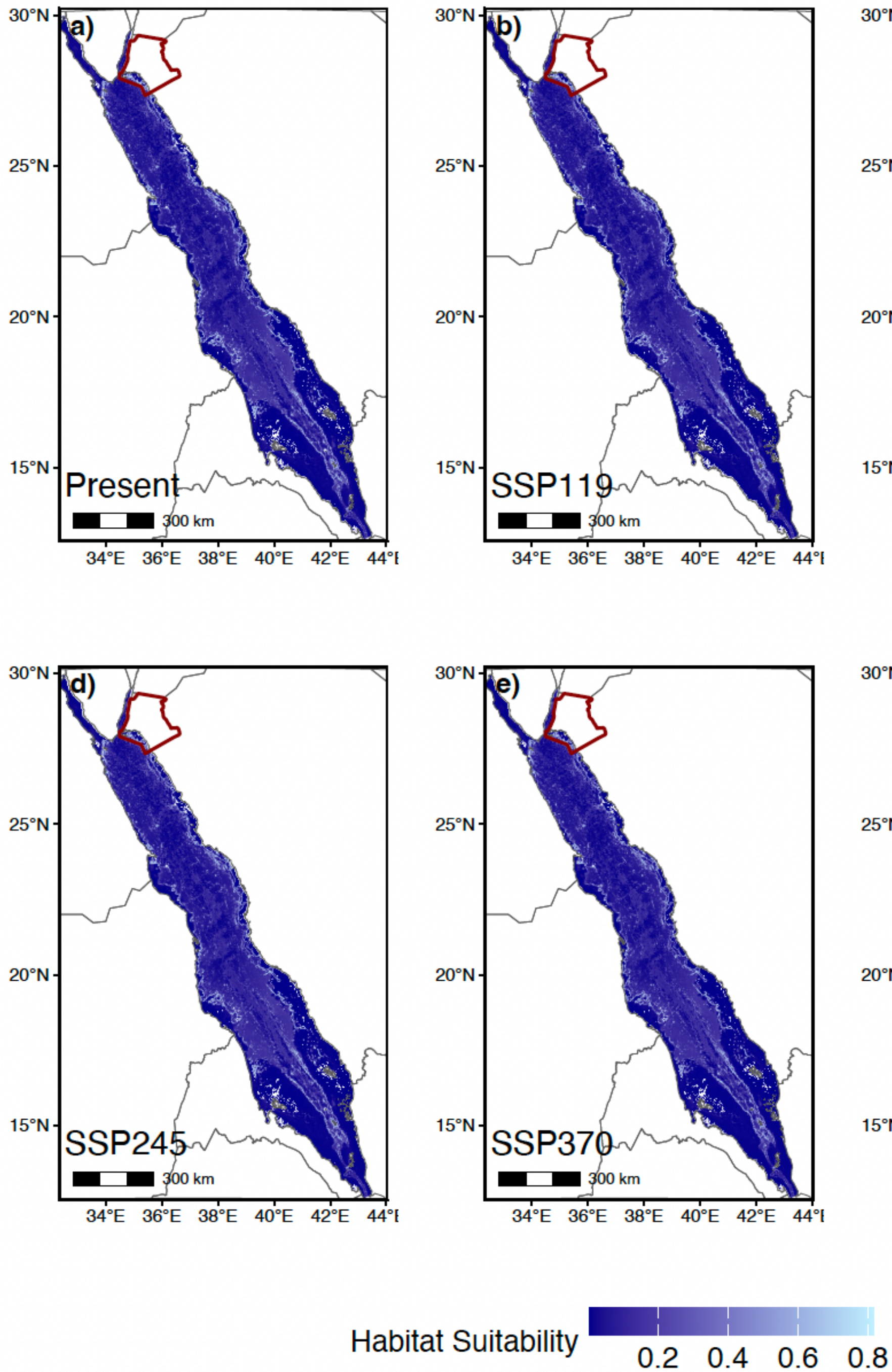

Supplementary Figure 25: Models for all scenarios for *Stylaster tritoni* generated with Maxent.

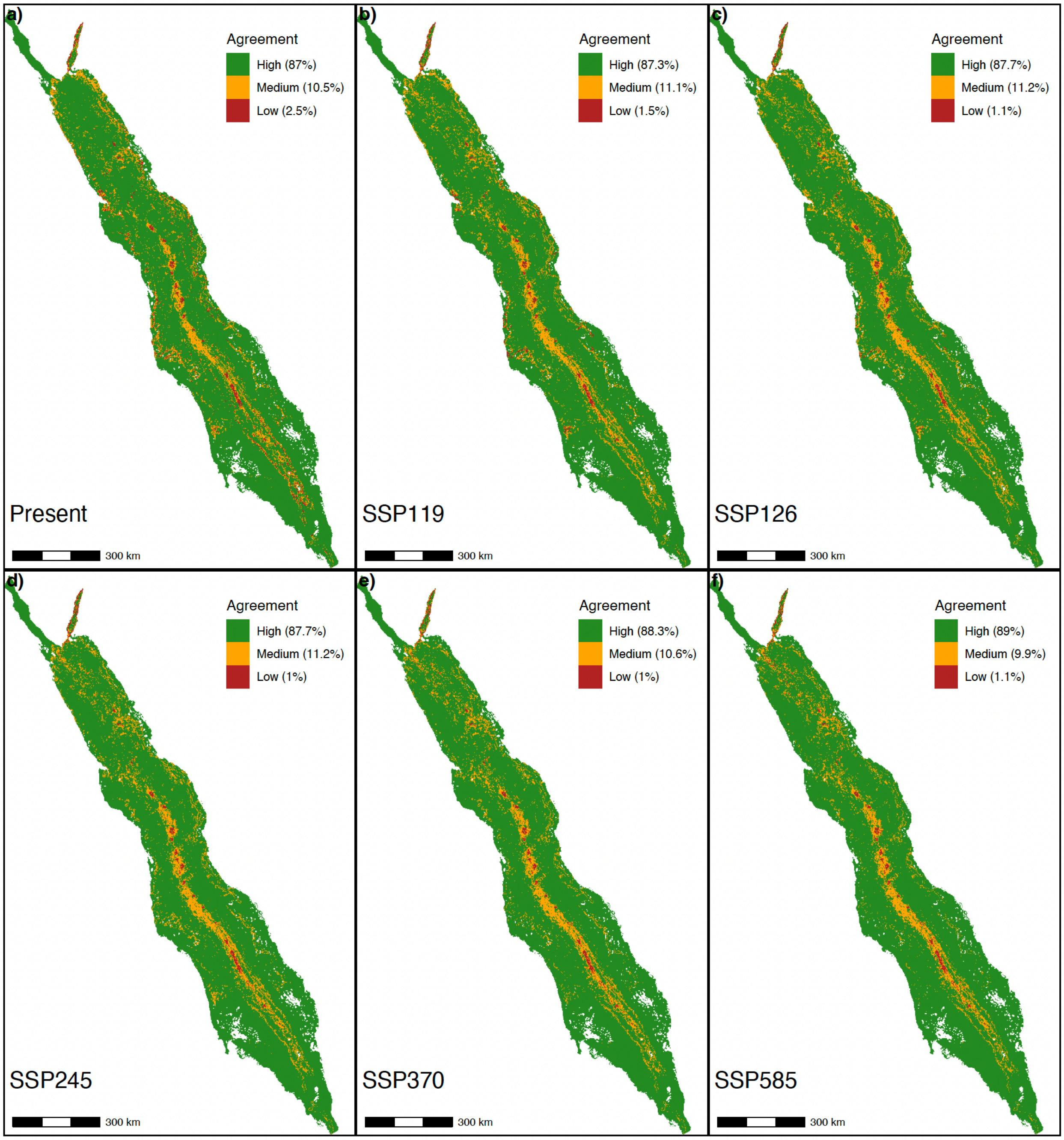

Supplementary Figure 26: Model agreement between Boosted Regression Tree and Maxent models for *Acanthogorgia* spp. The proportion of area assigned to each category is provided in brackets.

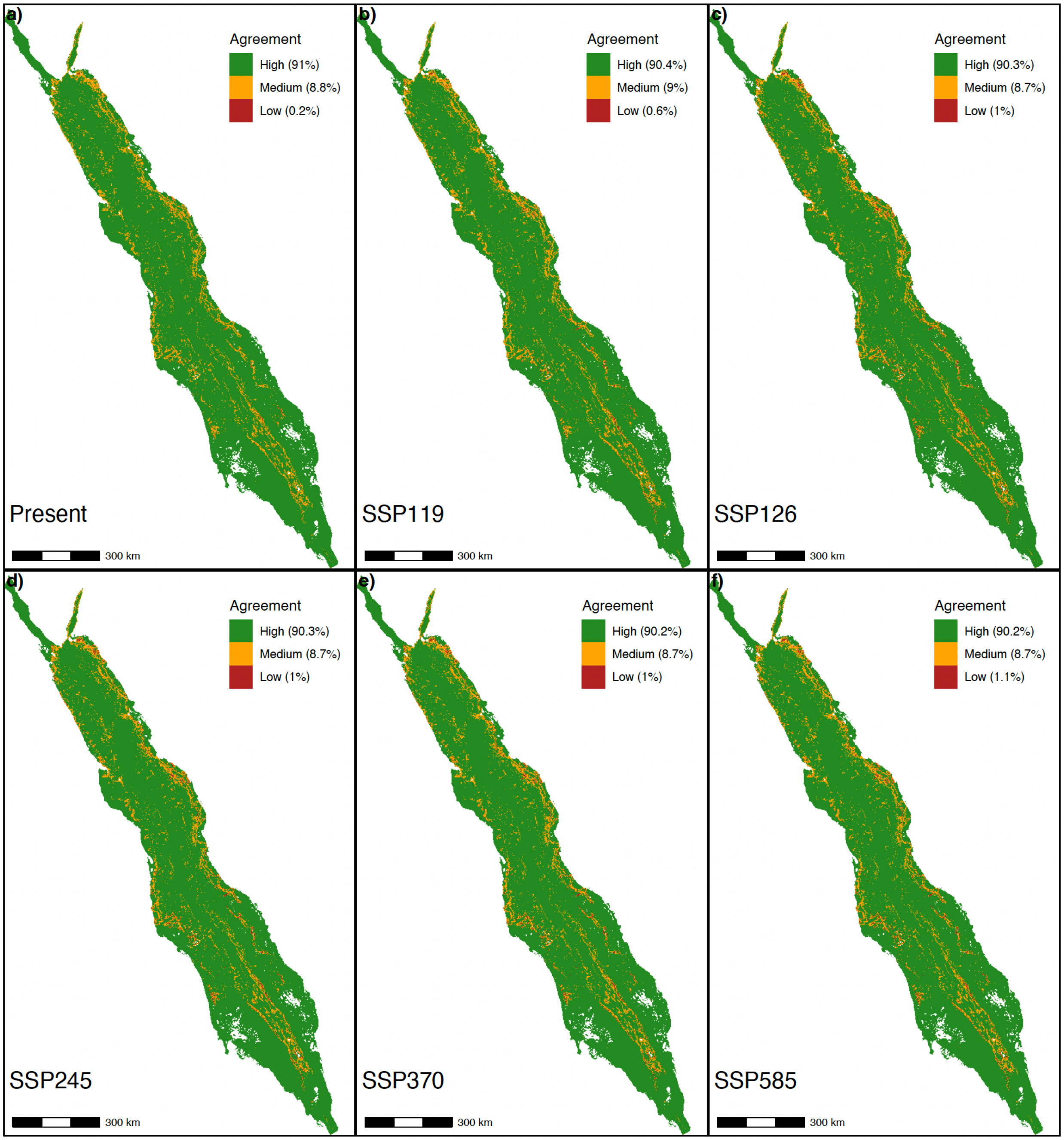

Supplementary Figure 27: Model agreement between Boosted Regression Tree and Maxent models for *Bathypathes thermophila*. The proportion of area assigned to each category is provided in brackets.

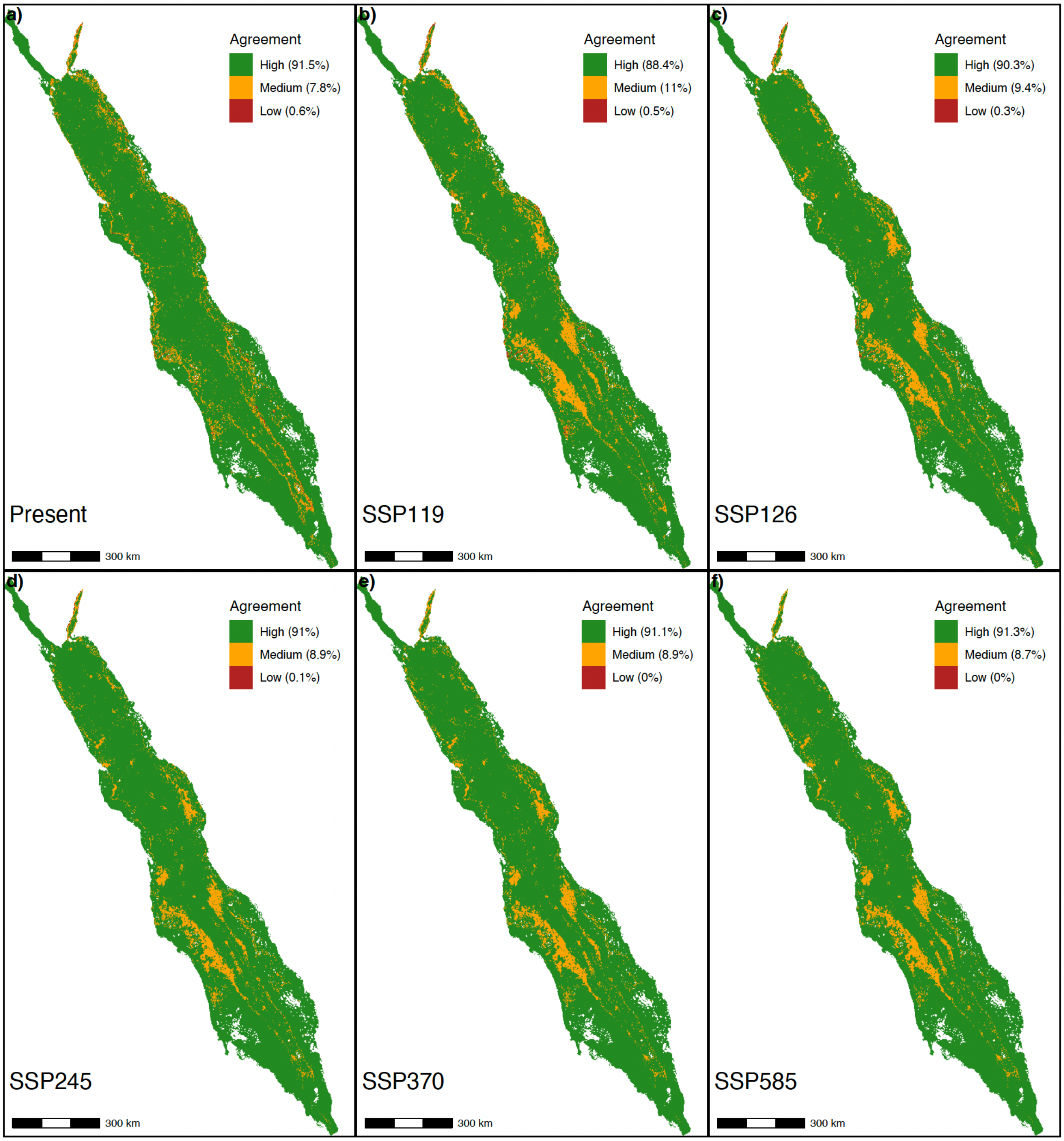

Supplementary Figure 28: Model agreement between Boosted Regression Tree and Maxent models for *Madracis interjecta*. The proportion of area assigned to each category is provided in brackets.

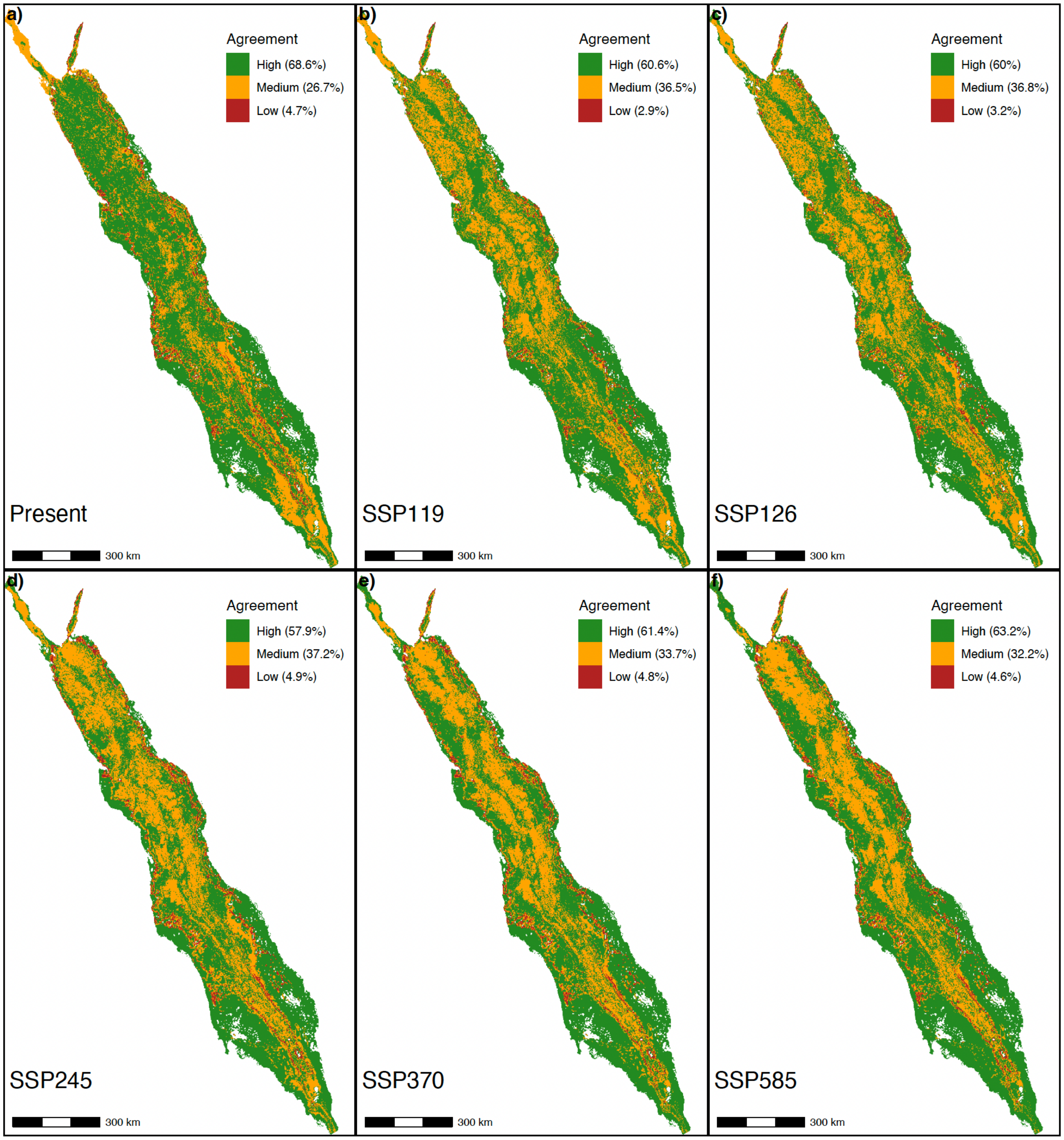

Supplementary Figure 29: Model agreement between Boosted Regression Tree and Maxent models for *Stylaster tritoni*. The proportion of area assigned to each category is provided in brackets.

Supplementary Table 1: Predictor variables used in the models, explanations of their importance and references. VRM is Vector Ruggedness Measure.

Variable	Importance	References
Depth	Can be a proxy for light, particularly in the mesophotic zone.	Pyle & Copus, 2019
Mean pH	pH influences the calcium carbonate building capacity of corals.	Venn et al., 2013
Slope	Increased slope is often correlated to hard substrate, required by corals for attachment.	Young, 2009
Max. temp<br>*Mean temp*<br>*Min. temp*	Temperature may control both upper and lower bathymetric limits of deep-sea corals. Maximum and minimum values can identify outliers in usual trends.	Buhl-Mortensen et al., 2015
Max. velocity X<br>*Min. velocity X*<br>*Max. velocity Y*<br>*Min. velocity Y*	Adequate flow is required for food availability to corals. Both water speeds that are too fast and too slow can limit food availability.	Moreno Navas et al., 2014
VRM	Higher VRM increases variability among seafloor and leads to increased likelihood of hard substrate.	Wilson et al., 2007

Supplementary Table 2: The locations of each observation of the four coral taxa, *Acanthogorgia* spp., *Bathypathes thermophila*, *Madracis interjecta* and *Stylaster tritoni* identified from video imagery in the mesophotic and deep eastern Red Sea. See separate electronic material for table.

Supplementary Table 3: The average interaction depth and shrinkage parameters for each Boosted Regression Tree (BRT) model.

Taxa	Interaction Depth	Shrinkage Parameter
Acanthogorgia spp.	2	0.0064
Bathypathes thermophila	3.5	0.0014
Madracis interjecta	1.8	0.0570
Stylaster tritoni	3.2	0.0425

References

Buhl-Mortensen, L., Olafsdottir, S. H., Buhl-Mortensen, P., Burgos, J. M., & Ragnarsson, S. A. (2015). Distribution of nine cold-water coral species (Scleractinia and Gorgonacea) in the cold temperate North Atlantic: Effects of bathymetry and hydrography. *Hydrobiologia*, *759*(1), 39-61. https://doi.org/10.1007/s10750-014-2116-x

Moreno Navas, J., Miller, P. L., Henry, L.-A., Hennige, S. J., Roberts, J. M., & Hewitt, J. (2014). Ecohydrodynamics of cold-water coral reefs: A case study of the Mingulay Reef Complex (Western Scotland). *PLOS One*, *9*(5), e98218. https://doi.org/10.1371/journal.pone.0098218

Pyle, R. L., & Copus, J. M. (2019). Mesophotic coral ecosystems: Introduction and overview. In *Mesophotic coral ecosystems* (pp. 3-27).

Venn, A. A, Tambutté, E., Holcomb, M., Laurent, J., Allemand, D. & Tambutté, S. (2013) Impact of seawater acidification on pH at the tissue–skeleton interface and calcification in reef corals, *Proceedings of the National Academy of Science.* 110 (5) 1634-1639, https://doi.org/10.1073/pnas.1216153110

Wilson, M. F. J., O'Connell, B., Brown, C., Guinan, J. C. & Grehan, A. J. (2007) Multiscale terrain analysis of multibeam bathymetry data for habitat mapping on the continental slope. *Marine Geodesy* 30, 3–35

Young, C. M. (2009). Communities on Deep-Sea Hard Bottoms. In M. Wahl (Ed.), *Marine Hard Bottom Communities: Patterns, Dynamics, Diversity, and Change* , 206, 39-60. Springer.
